# Iron-Catalyzed Alkylation–Reduction of *N*‑Methyl Nitrones via LMCT Activation

**DOI:** 10.1021/acs.orglett.5c04761

**Published:** 2025-12-04

**Authors:** Renan de O. Gonçalves, Jeimy A. C. Vélez, Natalí P. Debia, Pedro H. R Oliveira, Allya Larroza, Diego Alves, Márcio W. Paixão

**Affiliations:** † Laboratory for Sustainable Organic Synthesis and Catalysis, Chemistry Department, Federal University of São Carlos, UFSCar, São Carlos, São Paulo 13565-905, Brazil; ‡ Chemistry Institute, Federal University of Rio de Janeiro, UFRJ, Rio de Janeiro, Rio de Janeiro 21941-902, Brazil; § Laboratório de Síntese Orgânica Limpa, LASOL, CCQFA, 37902 Universidade Federal de Pelotas, UFPel, P.O. Box 354, 96010-900 Pelotas, Rio Grande do Sul, Brazil

## Abstract

*N*-Monomethyl secondary amines are privileged structural motifs that frequently occur in pharmaceuticals and bioactive molecules. However, their synthesis often requires harsh conditions or suffers from poor selectivity. Here, we report a redox-neutral, iron-catalyzed strategy that enables the efficient formation of these motifs via a tandem alkylation–reduction of (*Z*)-*N*-methyl nitrones. The transformation proceeds through an Fe­(III)-mediated ligand-to-metal charge transfer (LMCT) process that generates alkyl radicals from readily available carboxylic acids under visible light irradiation, followed by Fe­(II)-promoted N–O bond cleavage of the resulting hydroxylamines. This dual catalytic reactivity provides a straightforward and sustainable route to *N*-monomethyl amines under mild conditions, without the need for exogenous reductants, ligands, or precious metals. The method exhibits a broad substrate scope, excellent functional group tolerance, and high selectivity, highlighting its potential as a practical platform for late-stage amine diversification.

## Graphical abstract

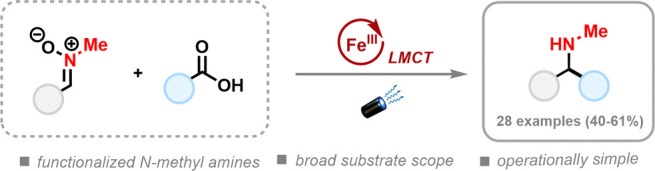

N-monomethyl secondary amines are valuable structural motifs in organic synthesis,^1^ widely presented in pharmaceuticals, natural products, and agrochemicals. They appear in several blockbuster drugs, including fluoxetine, sertraline, l-epinephrine, betahistine, and duloxetine (Figure 1A).^2^ The introduction of an *N*-methyl group can profoundly influence the biological profile of these compounds, enhancing lipophilicity, bioavailability, and metabolic stability, frequently leading to superior drug-like properties when compared to their nonmethylated counterparts.^3^ Given their widespread importance, the development of efficient, selective, and sustainable methods for accessing *N*-monomethyl secondary amines from simple precursors remains a key challenge in synthetic organic chemistry.^4^ Traditional approaches for *N*-methylation typically rely on the use of methylation agents such as methyl halides, dimethyl sulfate, or diazomethane – methods that often suffer from poor selectivity and the formation of undesired mixtures of mono- and dimethylated amines.^5^ Alternatively, reductive amination strategies employing formaldehyde or carbon dioxide have offered more sustainable options but still commonly require harsh reaction conditions or the use of stoichiometric reducing agents.^6^ More recently, transition-metal-catalyzed reductions of nitroarenes with methanol have emerged as promising dual strategies via in situ methyl transfer.^7^ Despite these advances, the need for operationally simple, selective, and environmentally benign methods for the synthesis of *N*-monomethyl amines persists.

**Figure 1:**
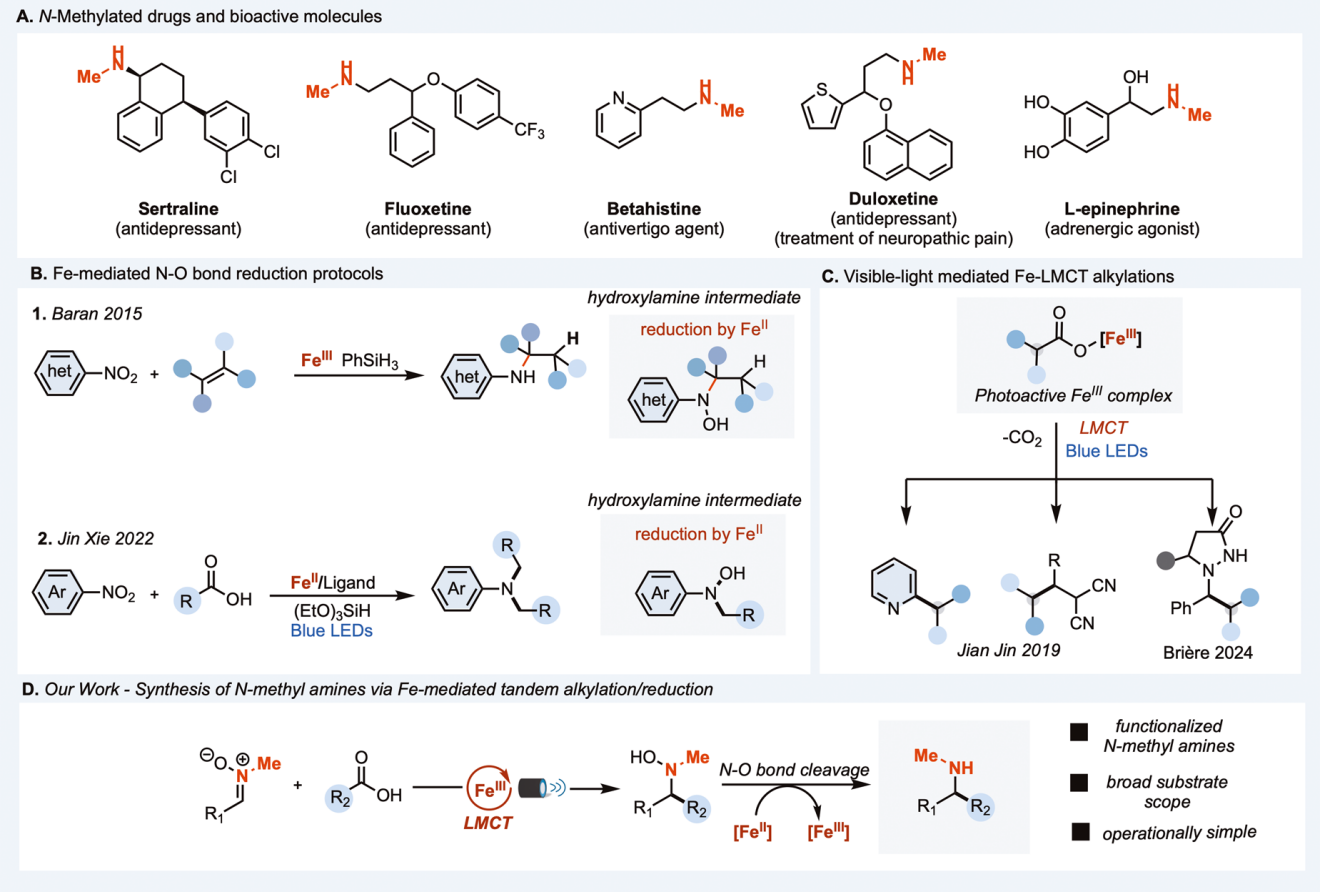
(A) *N*-methylamines as bioactive compounds. (B) Synthetic approaches involving Fe­(II)-mediated reduction of hydroxylamine intermediates. (C) Fe-LMCT alkylation reactions. (D) Catalytic Fe­(III)/Fe­(II)-mediated alkylation/reduction of *N*-Me nitrones via LMCT.

In this context, *N*-methyl nitrones have garnered attention as bench-stable and readily accessible substrates, extensively employed as radical acceptors in visible-light-mediated transformations, that typically afford hydroxylamine derivatives.^8^ We envisioned that such nitrones could serve as versatile platforms for photochemical radical functionalization followed by reductive conversion into secondary amines. In 2016, Baran and co-workers elegantly demonstrated that catalytic Fe­(II) can mediate N–O bond cleavage of hydroxylamine intermediates during the hydroamination of nitroarenes (Figure 1B).^9^ Building on this concept, Xie and co-workers recently reported an Fe­(III)/Fe­(II)-catalyzed transformation involving ligand-to-metal charge transfer (LMCT)-driven decarboxylative alkylation of nitroarenes, followed by Fe­(II)-promoted reduction of the resulting hydroxylamine intermediates to finally generated tertiary amines (Figure 1B).^10^ These findings, alongside other studies on Fe­(II)-promoted N–O bond cleavage, underscore the potential of iron catalysis for the mild and selective reduction of nitrogen–oxygen bonds.^11^

Concurrently, the photochemistry of Fe­(III)–carboxylate complexes has emerged as a powerful and sustainable platform for radical generation via LMCT under visible-light irradiation.^12^ This approach involves the coordination of carboxylic acids to Fe­(III), forming a photoactive complex that, upon excitation, undergo inner-sphere charge transfer leading to homolytic Fe–O bond cleavage and extrusion of CO_2_ to generate alkyl radicals.^12^ By overcoming the challenges associated with the ultrafast deactivation of Fe-based excited states and eliminating the need for costly photocatalysts with finely tuned redox properties, Fe-LMCT provides an attractive and sustainable alternative for photochemical transformations.^13^

Following the pioneering work of Sugimori in 1986, who employed stoichiometric Fe­(III) salts for radical alkylation of heteroarenes, numerous catalytic Fe-based LMCT systems have since been developed.^14^ Jin and co-workers reported Fe-catalyzed Minisci-type reactions using external oxidants, and later expanded this reactivity to redox-neutral alkylation of olefins and azodicarboxylates through careful ligand selection.^15^ More recently, Brière’s group demonstrated efficient ligand-free Fe-LMCT-catalyzed alkylation of azomethine imines, highlighting the growing versatility and operational simplicity of this platform (Figure 1C).^16^

Building on these insights, we envisioned a unified strategy that integrates Fe­(III)-mediated LMCT-driven radical generation with Fe­(II)-promoted N–O bond cleavage within a single catalytic platform (Figure 1D). We hypothesized that this dual reactivity could be orchestrated under redox-neutral conditions by employing *N*-methyl nitrones as electrophilic radical acceptors. Upon alkyl radical addition, these nitrones would generate hydroxylamine intermediates amenable to Fe­(II)-mediated reduction. The interplay between the LMCT-initiated radical generation and subsequent N–O cleavage offers a mechanistically straightforward and operationally simple route to *N*-monomethyl secondary amines from readily available precursors.

To probe our hypothesis for the Fe-mediated alkylation/reduction, we began by evaluating key reaction parameters and product distribution, using *N*-methyl nitrone **1** and the carboxylic acid **2a** as model substrates. To our delight, in the presence of Fe_2_(SO_4_)_3_·6H_2_O (10 mol %) and Cs_2_CO_3_ (20 mol %) in DCM under blue LED irradiation (456 nm), the desired transformation proceeded smoothly, affording amine **4** in 61% isolated yield, with no detectable formation of the hydroxylamine intermediate **3** (see the Supporting Information (SI) for the complete optimization).

With the optimal conditions in hand, we next explored the scope and limitations of the decarboxylative tandem alkylation/reduction with respect to the nitrone coupling partner (Figure 2a). The methodology was found to be broadly applicable across a range of substrates. Nitrones featuring electron-withdrawing groups, including *p*-fluoro (**5**, 52%) and *p*-trifluoromethyl (**6**, 43%), were well tolerated.

**Figure 2:**
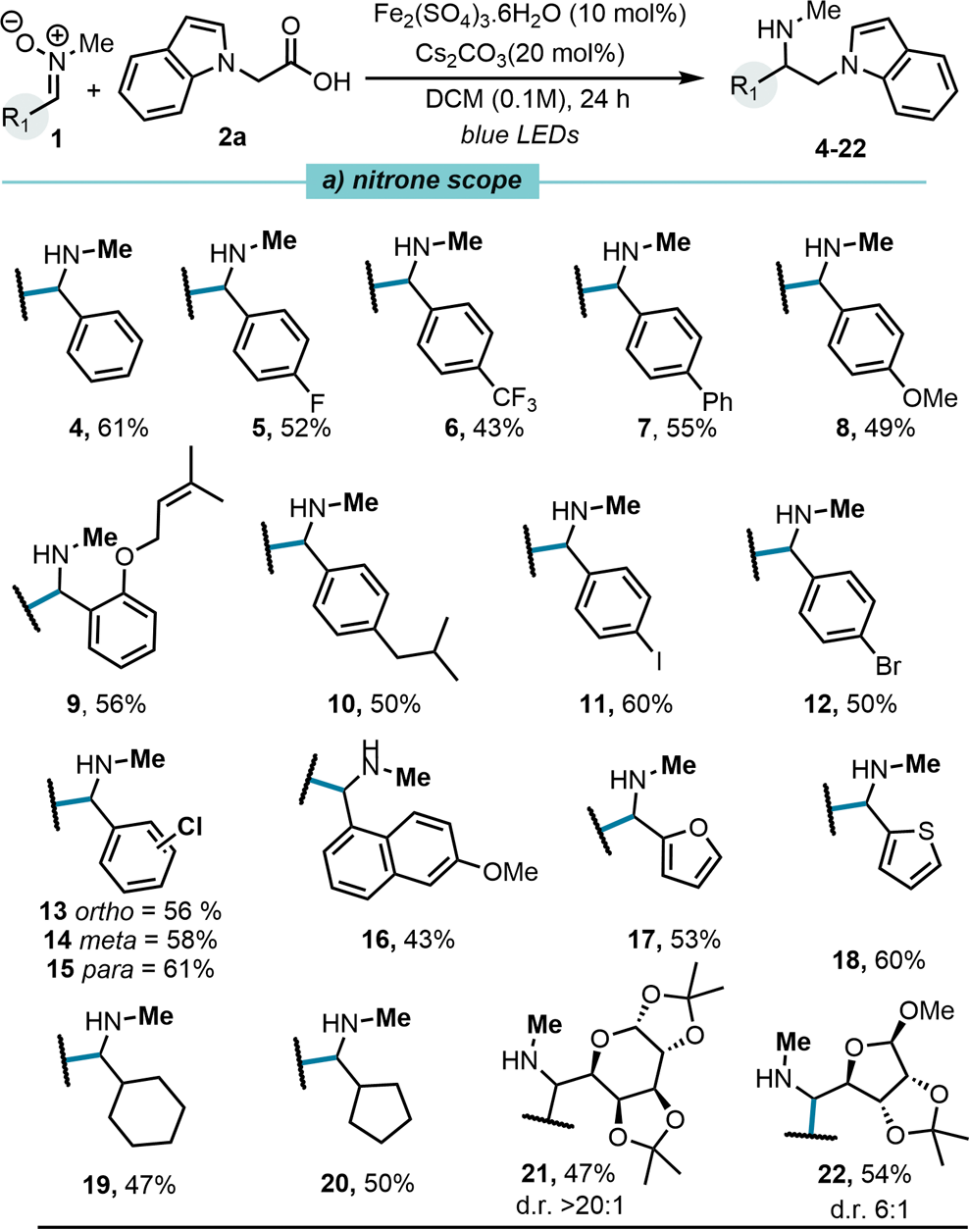
Nitrone (0.15 mmol), alkyl radical precursor (0.225 mmol, 1.5 equiv), Fe­(III) catalyst (10 mol %) and Cs_2_CO_3_ (20 mol %) in DCM (0.1 M) were subjected to blue LED irradiation (34 W Kessil lamp, λ_*max*_ = 456 nm) for 24 h.

Aryl-substituted nitrones, including *p*-phenyl (**7**, 55%), also delivered the corresponding amines in good yields. Electron-rich nitrones, bearing a *p*-methoxy (8, 49%), an *o*-allyl substituent (**9**, 56%), or an isobutyl group (**10**, 50%) were compatible under standard conditions. Halogenated nitrones containing *p*-I (**11**, 60%) and *p*-Br (**12**, 50%) substituents—valuable handles for further cross-coupling protocols—reacted smoothly. Nitrones with *ortho*-, *meta*-, and *para*-chloro groups (**13**–**15**) provided the corresponding products in yields ranging from 56–61%. A more sterically hindered substrate bearing a *p*-methoxy-naphthyl group (**16,** 43%) was also suitable under the established protocol. Heterocyclic scaffolds frequently encountered in medicinal chemistry, such as furan (**17**, 53%) and thiophene (**18**, 60%), were efficiently transformed. In addition, alkyl-substituted nitrones, including cyclohexyl (**19**, 47%) and cyclopentyl (**20**, 50%) derivatives, were well tolerated, underscoring the method’s compatibility with nonaromatic systems. The methodology also proved effective for the functionalization of glycosyl nitrones derived from protected d-galactose (**21**, 47%) and d-ribose (**22**, 54%). Notably, both transformations proceeded with good diastereoselectivity (d.r. > 20:1 and 6:1 respectively), underscoring the potential of this strategy for the synthesis of stereodefined amines from chiral nitrones.

With the nitrone scope established, we then explored the compatibility of a range of carboxylic acids under the optimized reaction conditions (Figure 3). In contrast to Ir-catalyzed systems—which often rely on excess of acid and strict exclusion of oxygen and moisture^17^—the Fe-catalyzed approach benefits from a more efficient unimolecular decarboxylation mechanism. This feature allows for milder and more practical reaction conditions.^18^ Notably, the reaction was most efficient with activated carboxylic acids, particularly those featuring benzylic or α-heteroatom substituents. These results are consistent with the formation of stabilized carbon-centered radicals, which facilitate the alkylation step.

**Figure 3:**
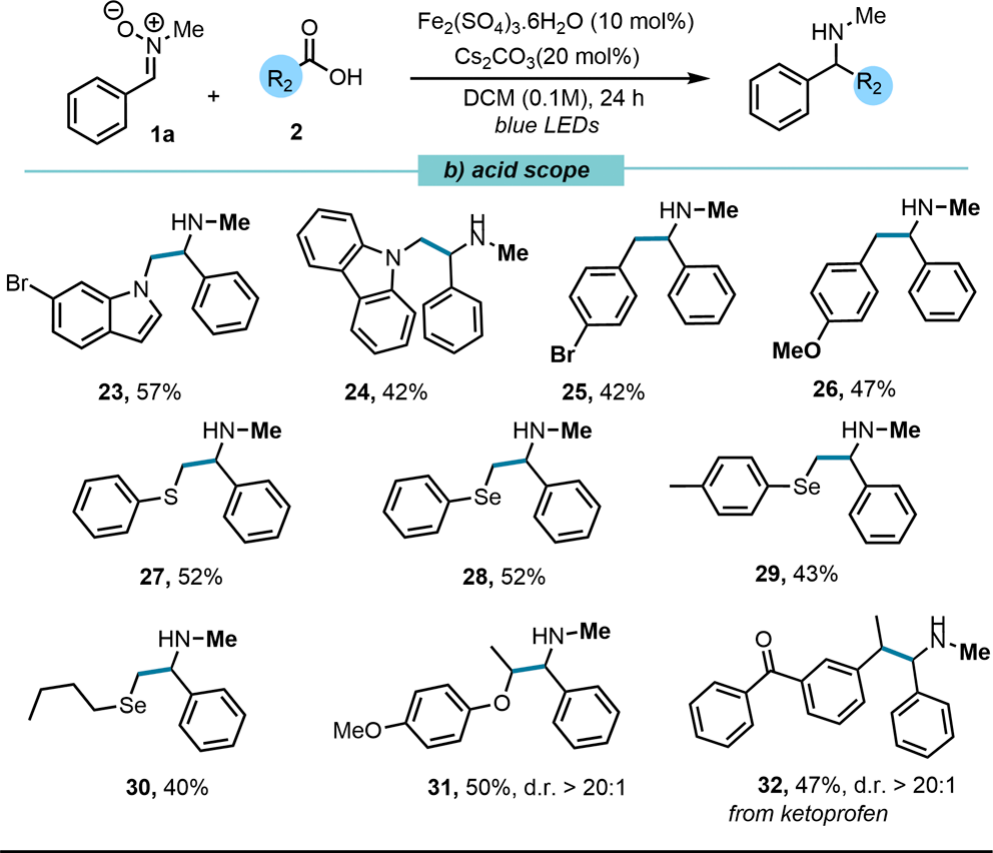
Nitrone (0.15 mmol), alkyl radical precursor (0.225 mmol, 1.5 equiv), Fe­(III) catalyst (10 mol %) and Cs_2_CO_3_ (20 mol %) in DCM (0.1 M) were subjected to blue LED irradiation (34 W Kessil lamp, λ_*max*_ = 456 nm) for 24 h.

Further exploring the α-amino radical addition manifold, we evaluated carboxylic acids derived from a bromo-substituted indole and carbazole. Both substrates delivered the corresponding amines (**23**, 57%; **24**, 42%) in modest yields, highlighting the method’s applicability to nitrogen-containing systems. Benzylic radicals also proved competent under the reaction conditions. Carboxylic acids, e.g., *p*-bromo and *p*-methoxy phenylacetic acids, afforded the corresponding products in moderate yields (**25**, 42%; **26**, 47%). Sulfur- and selenium-based motifs are of particular interest in medicinal chemistry due to their distinctive electronic and biological properties.^19^ Notably, our methodology enabled the generation of α-chalcogen-centered radicals. Carboxylic acids bearing sulfur as well as alkyl and aryl selenium groups furnished the corresponding amines (**27**–**30**) in moderate yields, underscoring the utility of this protocol for accessing novel chalcogen-containing scaffolds. In addition, an α-alkoxy radical was compatible with the transformation, affording amine **31** in 50% yield, and demonstrating tolerance toward oxygenated substrates. Finally, the late-stage functionalization of ketoprofen, a widely used NSAID, furnished amine **32** in 47%, highlighting the method’s utility in modifying drug-like molecules (Figure 3).

To gain insight into the mechanism of our iron-catalyzed transformation, we carried out a series of control experiments and spectroscopic studies. The addition of 2,2,6,6-tetramethylpiperidinyloxyl (TEMPO) completely suppressed product formation, and the corresponding TEMPO–indole adduct was detected by HRMS analysis, thereby confirming the involvement of alkyl radicals in the reaction manifold (see the SI for full details). Moreover, UV–vis analysis revealed a pronounced bathochromic shift upon mixing the iron salt with the carboxylate, with the appearance new absorption band around 427 nm. This observation is consistent with the formation of a photoactive Fe­(III)-carboxylate complex, which likely engages in a ligand-to-metal charge transfer (LMCT) event during the visible-light irradiation. These findings are in alignment with mechanistic proposals by Brière^16^ and Guérinot,^20^ who have similarly reported LMCT-triggered alkyl radical generation in ligand-free iron systems.

We next sought to investigate the role of the hydroxylamine intermediate in the reoxidation of Fe­(II) to Fe­(III). Cyclic voltammetry experiments revealed a half-wave potential for FeCl_3_ (E_0_[Fe­(II)/Fe­(III)] = −0.11 V vs Ag/AgCl in acetonitrile), compatible with the reduction potential of the putative *N*-alkylhydroxylamine intermediate (E_0_[R–N­(Me)­OH/R–NHMe] = 0.19 V vs Ag/AgCl in acetonitrile), thereby supporting a thermodynamically favorable redox interplay (see the SI for full electrochemical data).

To gain further insight into the source of the catalytically active Fe­(II) species, we evaluated whether additional reductive processes could operate in situ. Control experiments using increased acid loading (2.0 equiv) led to diminished yields (37%), likely due to competitive coordination of Fe­(III) and reduced availability of the active complex. Conversely, when the isolated hydroxylamine **3** was subjected to the reaction conditions in the presence of Fe_2_(SO_4_)_3_·6H_2_O and blue light, conversion to the corresponding amine was observed (see the SI for full details). This result indicates that Fe­(III) can promote hydroxylamine reduction under photochemical conditions, suggesting that Fe­(III) species may undergo in situ disproportionation or photoinduced reduction to Fe­(II), thereby sustaining the catalytic cycle without requiring additional consumption of carboxylic acid. Nevertheless, the photoinduced reduction of hydroxylamine by a Fe­(III) species cannot be ruled out.

Taken together, these results, along with literature precedents, support a mechanistic framework involving the formation of a trivalent Fe­(III)–carboxylate complex upon initial deprotonation of the carboxylic acid. This complex undergoes photoexcitation followed by a ligand-to-metal charge transfer (LMCT) event, generating an Fe­(II) species and a carboxyl radical. After decarboxylation, the resulting nucleophilic alkyl radical adds to the *N*-methyl nitrone **1**, forming a radical intermediate that is reduced and protonated to give the hydroxylamine **3**. The latter is converted into the corresponding functionalized *N*-monomethyl amine **4**, concomitant with the reoxidation of Fe­(II) to Fe­(III), thereby closing the catalytic cycle (Figure 4).

**Figure 4:**
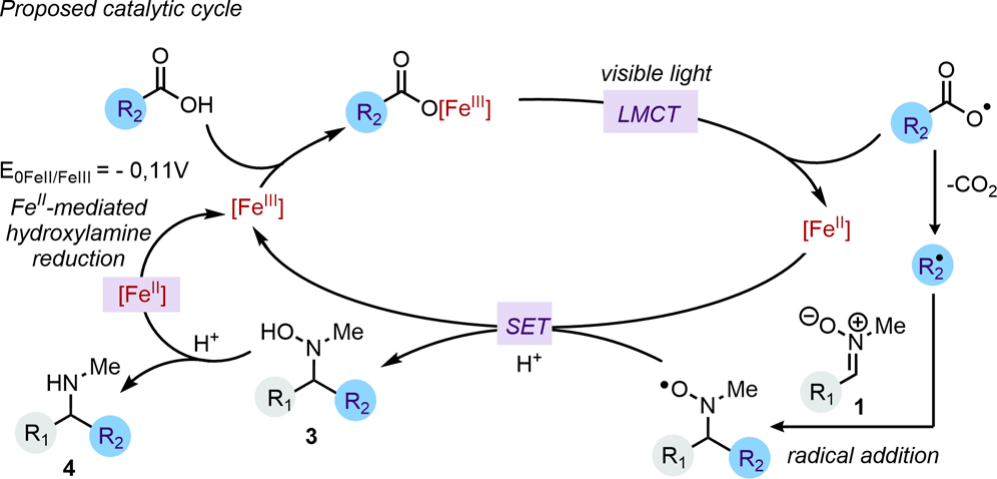
Proposed catalytic cycle for the LMCT alkylation/reduction.

In summary, we have developed a mild and efficient iron-catalyzed decarboxylative alkylation/reduction protocol for the synthesis of *N*-monomethyl amines from readily accessible *N*-methyl nitrones and carboxylic acids. The reaction proceeds under visible light irradiation without the need for external ligands or additives, leveraging a ligand-to-metal charge transfer (LMCT) event to generate alkyl radicals. The broad substrate scope, accommodating aryl, heteroaryl, and glycosyl nitrones, as well as benzylic, α-heteroatom, and chalcogen-containing carboxylic acids, demonstrates the versatility of the transformation. Mechanistic investigations, including UV–vis and electrochemical analyses, support a catalytic cycle involving Fe­(III)/Fe­(II) redox processes and reduction of a hydroxylamine intermediate. The operational simplicity, functional group tolerance, and mechanistic insight showcased here highlight the potential of this iron-based platform as a sustainable strategy for the synthesis of this valuable class of amines.

## Supplementary Material



## Data Availability

The data underlying this study are available in the published article and its Supporting Information.

## Notes

Additional experimental details, NMR spectra, and supplementary figures (PDF)
