# Supplementary material for: Iron-Catalyzed Alkylation–Reduction of N‑Methyl Nitrones via LMCT Activation
Source: Org Lett. 2025 Dec 4;27(50):14118–23. doi: 10.1021/acs.orglett.5c04761 (PMC12723735; doi:10.1021/acs.orglett.5c04761)
Supplement: Supplementary file 1 [file ol5c04761_si_001.pdf]

Supporting Information

Iron-Catalyzed Alkylation–Reduction of *N*-Methyl Nitrones via LMCT Activation

Renan de O. Gonçalves,[a] Jeimy A. C. Vélez,[a] Natalí P. Debia,[a] Pedro H. R Oliveira,[b,*] Allya Larroza,[c] Diego Alves [c] and Márcio W. Paixão[a,*]

[a]Laboratory for Sustainable Organic Synthesis and Catalysis – Chemistry Department – Federal University of São Carlos – UFSCar, São Carlos, São Paulo, 13565-905, Brazil. E-mail: mwpaixao@ufscar.br

[b]Chemistry Institute – Federal University of Rio de Janeiro – UFRJ, Rio de Janeiro, Rio de Janeiro, 21941-902, Brazil.

[c]Laboratório de Síntese Orgânica Limpa – LASOL, CCQFA, Universidade Federal de Pelotas – UFPel, P. O. Box 354, 96010-900, Pelotas, RS, Brazil

Summary

1. General considerations S3

2. Synthesis of *N*-methyl nitrones S3

3. General procedure for the synthesis of carboxylic acid derivatives S5

4. Optimization studies S6

5. General procedure S7

6. Scale-up experiment S8

7. Mechanistic studies S9

   7.1 Investigation into the *in situ* formation of *N*-methylhydroxylamines S9

   7.2 Trapping experiment S10

   7.3 Mass spectrum of the crude reaction mixture S11

   7.4 Cyclic voltammetry measurements S12

   7.5 UV/Vis absorption spectroscopy S13

   7.6 Investigation of the reduction of *N*-methylhydroxylamines into *N*-methylamines S15

8 Compound characterization data S16

   8.1 Starting materials S16

   8.2 Scope for the nitrones S17

   8.3 Scope for the carboxylic acids S24

9 NMR spectra S28

1. General considerations

All reactions were prepared using standard Schlenk techniques and performed under a nitrogen atmosphere. HPLC-grade solvents were used in the photocatalyzed reactions. Unless otherwise noted, all reagents were purchased from commercial sources and used without further purification. All sensitive to air or moisture reactions were carried out in flame-dried glassware under a nitrogen atmosphere. Photocatalysts were purchased or synthesized unless otherwise noted. A 34 W Kessil H150 blue LED (λ_{max} = 456 nm) was used as the visible light source. Reactions were monitored by layer chromatography using Merck 60 F254 silica gel aluminum sheets, hexane/EtOAc as mobile phase and visualized by UV lamp, permanganate or thymol spots. Flash column chromatography was performed using silica gel 60 (230-400 mesh) and hexane/EtOAc as eluent systems. ^{1}H, ^{19}F and ^{13}C NMR spectra were recorded on Brucker NMR spectrometers (400 for ^{1}H, 100 for ^{13}C and 376 MHz for ^{19}F). Chemical shifts (δ) for the ^{1}H and ^{13}C spectra are given in ppm, residual solvent signals were used as a reference for the ^{1}H and ^{13}C NMR spectra, non-deuterated chloroform ($CDCl_3$): δ H = 7.26 ppm, δ C = 77.16 ppm). The values of the coupling constants (J) are given in Hertz. The multiplicities are described as: s = singlet, d = doublet, t = triplet, q = quartet, dd = doublet of doublets, dt = doublet of triplets, dq = doublet of quartets, m = multiplet. High-resolution mass spectra (HRMS) were recorded at HESI Quadrupole-Orbitrap (Q extractive focus, Thermo Scientific).

2. Synthesis of *N*-methyl nitrones

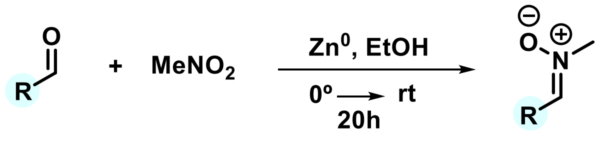

To a solution of aldehyde (1.0 mmol), nitromethane (4.0 mmol), and zinc powder (ZnO, 6.0 mmol) in 95% absolute ethanol (0.19 M), previously cooled in an ice bath, glacial acetic acid (7.0 mmol) was added dropwise. After complete addition, the reaction mixture was kept under stirring for 20 h, allowing it to gradually return to room temperature. At the end of the reaction, the suspension was filtered and washed with ethyl acetate. The filtrate was evaporated under reduced pressure, and the crude residue obtained was purified by column chromatography using ethyl acetate/methanol (9.5:0.5) as the mobile phase, furnishing the products.

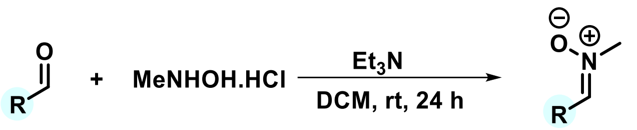

In a double-necked flask, previously flamed and cooled under nitrogen atmosphere, was added dichloromethane (0.2 M), anhydrous magnesium sulfate (10.39 mmol, 3.50 equiv.), *N*-Methylhydroxylamine hydrochloride (3.26 mmol, 1.10 equiv.), Et_3N (6.53 mmol, 2.20 equiv.) and the aldehyde (2.97 mmol, 1.00 equiv). The reaction solution was stirred vigorously for 24 hours at room temperature. The mixture was filtered and the DCM was evaporated under pressure. The crude was purified by flash cromatography. [1]

The reported compounds (**1a** – **19s**) were prepared and characterized according to the literature.

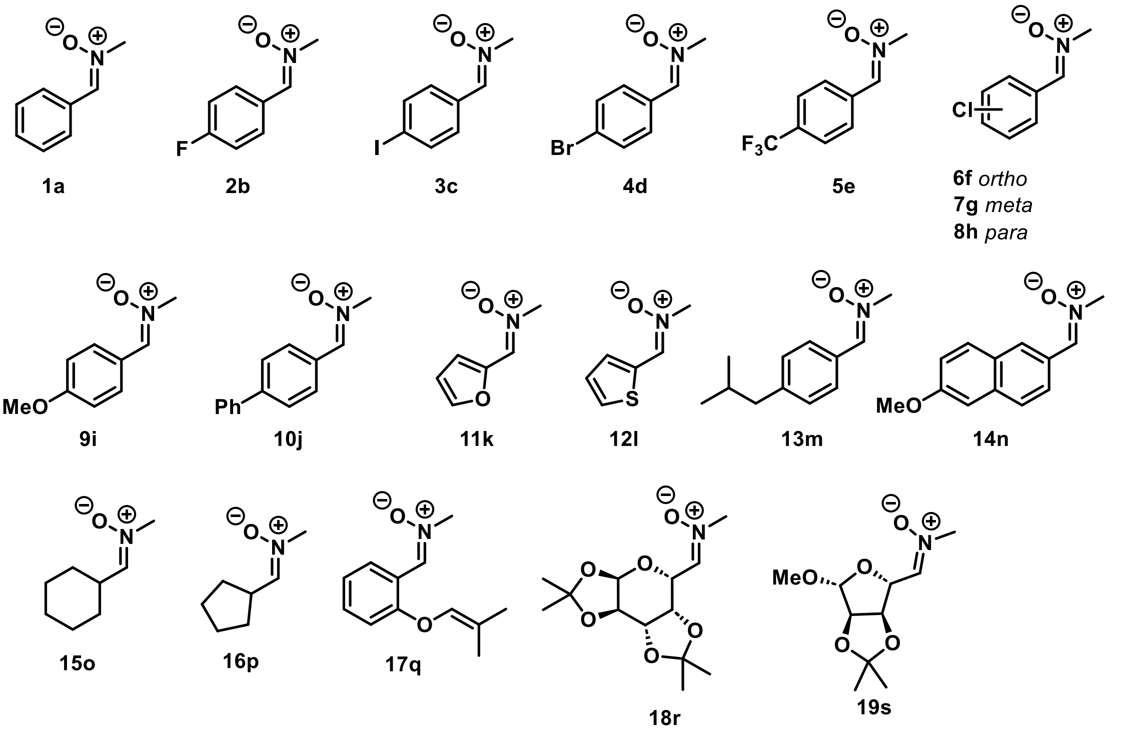

Scheme S1. Synthetized nitrones as starting materials.

[1] Aschwanden, P.; Kværnø, L.; Geisser, R. W.; Kleinbeck, F.; Carreira, E. M. *Org. Lett.* **2005**, 7, 25, 5741–5742.

Spectroscopic data of nitrones

1a, 8h [2]; 2b, 17q, 15o [3]; 3c, 4d, 5e, 6f, 11k, 16p [4]; 9i [5]; 7g, 12l, 14n [6].

3. General procedure for the synthesis of carboxylic acid derivatives

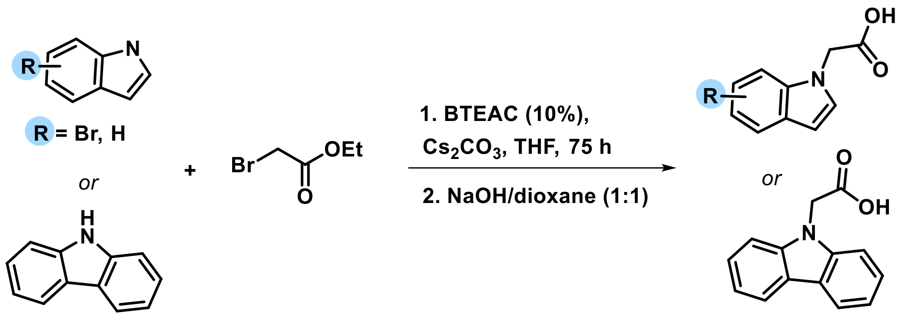

Acetic acid derivatives were synthesized using a modified procedure.[7] To a solution of the 1*H*-indole derivative or carbazole (1 equiv.) in anhydrous THF (40 mL) were added ethyl bromoacetate (1.5 equiv.), cesium carbonate (Cs_2CO_3, 1.5 equiv.), and benzyltriethylammonium chloride (BTEAC) (10 mol%). The reaction mixture was stirred vigorously at room temperature for 75 h. After this time, the reaction was filtered and the solvent removed under reduced pressure, affording the corresponding ester, which was used in the subsequent step without further purification. The crude material was then dissolved in a mixture of 1,4-dioxane (20 mL) and aqueous NaOH solution (1 M, 20 mL), and the mixture was stirred at room temperature overnight. After the reaction time, the mixture was carefully acidified with aqueous HCl solution (10%, to pH ≈ 3), and the organic phase was extracted with ethyl acetate (3 × 40 mL). The organic phases were combined, dried, and concentrated under reduced pressure. The crude residue obtained was purified by silica gel column chromatography, yielding the corresponding carboxylic acid.

The reported compounds (**2a**–**3c**) were prepared and characterized according to literature procedures.

---

[2] Pagoti, S.; Dutta, D.; Dash. *Adv. Synth. Catal.* **2013**, *355*, 3532–3538.
[3] Cordier, M.; Archambeau, A. *Org. Lett.* **2018**, *20*, 2265–2268.
[4] Li, T.; Liu, S.; Sun, Y.; Deng, S.; Tan, W.; Jiao, Y.; Zhang, Y.; Shi, F. *Angew. Chemie Int. Ed.* **2021**, *60*, 2355–2363.
[5] Andrade, M. M.; Barros, M. T.; Pinto, R. C. *Tetrahedron* **2008**, *64*, 10521–10530;
[6] He, N.; Yang, Y.; Qiao, Z.; Zhu, F.; Lin, J.; Song, X.; Jin, Y. *Tetrahedron Letters* **2023**, *128*, 154712.
[7] Leleu, L.; Martzel, T.; Fall, A.; Sanselme, M.; Levacher, V.; Oudeyer, S.; Brière, J. F. *Chem. Commun.* **2022**, *58*, 6100-6103. Brogan, J. T.; Stoops, S. L.; Lindsley, C. W. *ACS Chem. Neurosci.* **2012**, *3*, 658–664.

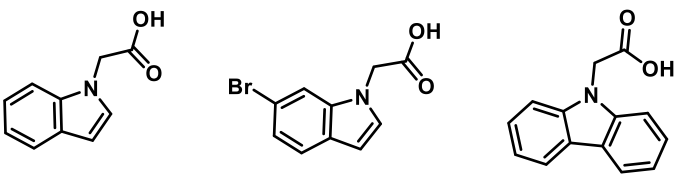

Scheme S2. Carboxylic Acid derivatives synthesized and used in this work.

Spectroscopic data of acetic acid derivatives

2a, 2b [6]; 3c [8].

4. Optimization studies

To probe our hypothesis for the Fe-mediated alkylation/reduction, we began by evaluating key reaction parameters and product distribution, using *N*-methyl nitrone **1a** and the carboxylic acid **2a** as model substrates. To our delight, in the presence of $Fe_2(SO_4)_3{\cdot}6H_2O$ (10 mol%) and Cs_2CO_3 (20 mol%) in DCM under blue LED irradiation (456 nm), the desired transformation proceeded smoothly, affording amine **4** in 61% isolated yield, with no detectable formation of the hydroxylamine intermediate **3** (entry 1, Table 1S). Control experiments revealed that the reaction in DMSO proceed more slowly, with time-dependent accumulation of intermediate **3,** followed by its conversion to **4**. After 48 hours, only the amine product was observed (entries 2–4, Table 1S), supporting our proposed Fe(III)–LMCT/Fe(II) alkylation/reduction pathway. Among the solvents evaluated, DCM proved to be optimal. In contrast, MeCN, DMF, and EtOH afforded exclusively the amine **4**, albeit in lower yields (entries 5–7, Table 1S). We further examined a series of Fe(III) complexes: $FeCl_3{\cdot}6H_2O$ performed similarly to $Fe_2(SO_4)_3{\cdot}6H_2O$, while the remaining complexes delivered lower efficiencies (entries 8–10, Table 1S). Slightly lower yields were observed when using reduced amounts of Cs_2CO_3, substitution by Na_2CO_3, or employing Et_3N as an organic base (entries 12–14, table 1S). Reversing the stoichiometry or increasing the acid loading (**2a**) to 2.0 equiv resulted in diminished yields (entries 15–16, Table 1S). The addition of a protic additive such as AcOH (0.5 equiv) completely inhibited product formation, likely due to competitive coordination to the Fe(III) species (entry 17, Table 1S). Performing the reaction under anhydrous conditions or without the freeze–pump–thaw degassing

[8] Grabowski, D.; Alef, S.; Becker, S.; Müller, U. Schnakenburg, G.; Höger, S. *Org. Chem. Front.* **2022**, *9*, 294-298.

procedure still afforded the amine **4** in slightly lower yield (43% for both), demonstrating that the process can tolerate both moisture and air to some extent (entries 18–19, Table 1S). Finally, no product formation was observed in the absence of base, iron catalyst, or light, confirming that all components are essential for the reaction to proceed (entries 20–21, Table 1S).

Table 1S. Optimization of the reaction conditions

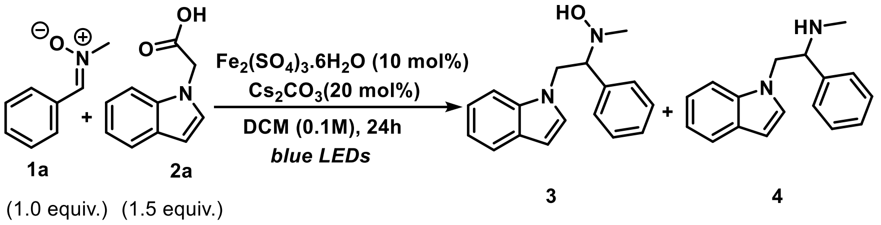

Entry	Conditions	Yield % **3/4**[b]
**1**	**None**	**61**
2[a]	3h	40/ n.d
3[a]	24h	13/ 41
4[a]	48h	n.d/ 53
5	DMF	29
6	MeCN	27
7	EtOH	29
8	$Fe(NH_3)(SO_4)_2.6H_2O$	45
9	$Fe(NO_3)_3$	35
10	$FeCl_3.6H_2O$	61
11	$Fe_2(SO_4)_3.6H_2O$ (15 mol%)	54
12	Cs_2CO_3 (10 mol%)	54
13	Na_2CO_3	53
14	Et_3N	54
15	Reversed stoichiometry	43
16	**2a** (2 equiv.)	37
17	AcOH (0.5 equiv.)	trace
18	Anhydrous solvent	43
19	Without freeze-pump-thaw	43
20	absence base	n.d
21	absence of Fe and light	n.d

Yields refer to isolated compounds. Conditions unless stated otherwise: (**1** 1 equiv, 0.15 mmol) **2** (1.5 equiv., 0.225 mmol) in 0.1 M of DCM as solvent under irradiation with a 456 nm blue LED light at room temperature for 24 hours. a) Reactions in entries 2–4 performed in DMSO. b) Single yield values indicate exclusive formation of **4**; **3** was not detected.

5. General procedure

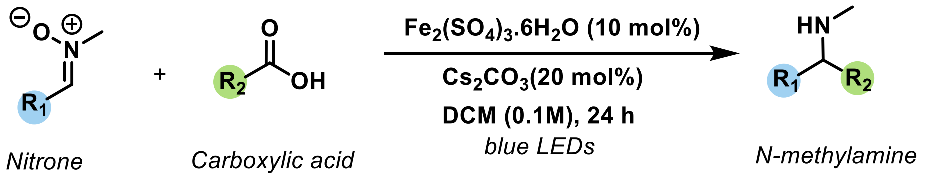

A dry borosilicate glass reaction tube equipped with a magnetic stir bar was charged with nitrone (0.15 mmol, 1.0 equiv), the corresponding carboxylic acid (0.225 mmol, 1.5 equiv), the Fe(III) catalyst (10 mol%), and Cs_2CO_3 (20 mol%). Dichloromethane (1.5 mL, 0.1 M) was added, and the tube was sealed with a PTFE/silicone septum and connected to a vacuum line. The solution was degassed three times using the freeze–pump–thaw method and stirred under 34 W Kessil H150 blue LED irradiation (λ_{max} = 456 nm) positioned 4 cm from the reaction vessel at room temperature for 24 hours. After completion of the reaction, the solvent was removed under reduced pressure, and the crude product was purified by column chromatography using a hexane/ethyl acetate (6:4) mixture to afford the target compounds.

6. Scale-up experiment

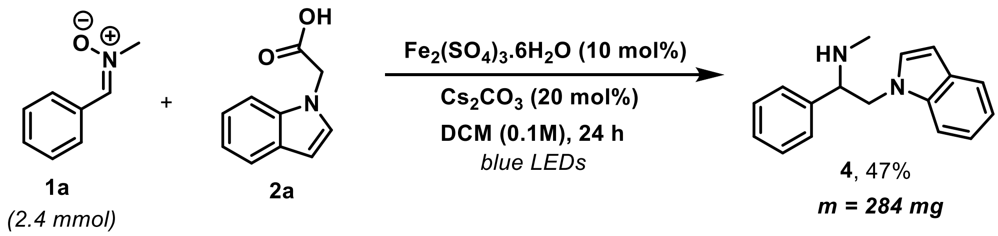

A dry borosilicate glass reaction tube equipped with a magnetic stir bar was charged with nitrone (2.4 mmol, 1.0 equiv), the 2-(1*H*-indol-1-yl)acetic acid (3.6 mmol, 1.5 equiv), the Fe(III) catalyst (0,240 mmol, 10 mol%), and Cs_2CO_3 (0,48 mmol, 20 mol%). DMSO (24 mL, 0.1 M) was added, and the tube was sealed with a PTFE/silicone septum and connected to a vacuum line. The solution was degassed three times using the freeze–pump–thaw method and stirred under irradiation with two 34 W Kessil H150 blue LEDs

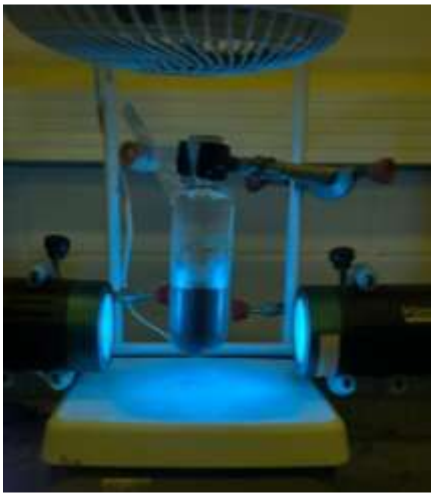

(λ_{max} = 456 nm) positioned 4 cm from the reaction vessel at room temperature for 24 hours. After completion of the reaction, ethyl acetate was added to the reaction mixture, which was washed three times with water and then with brine. The organic layer was dried over anhydrous sodium sulfate, filtered, and concentrated under reduced pressure. The crude product was purified by column chromatography using a mixture of hexane/ethyl acetate (6:4) as the eluent, affording the target compounds in 47% yield.

7. Mechanistic studies

7.1 Investigation into the *in situ* formation of *N*-methylhydroxylamines

With the aim of understanding the formation of the reaction intermediates, a time-course study of the transformation was carried out using DMSO as the solvent. The choice of DMSO is due to its ability to slow down the reaction progress compared to DCM, allowing for a more detailed analysis of the formation and consumption of the species over time. The obtained results are summarized in **Scheme S3.** The methodology followed the general procedure, using DMSO as the solvent instead of dichloromethane.

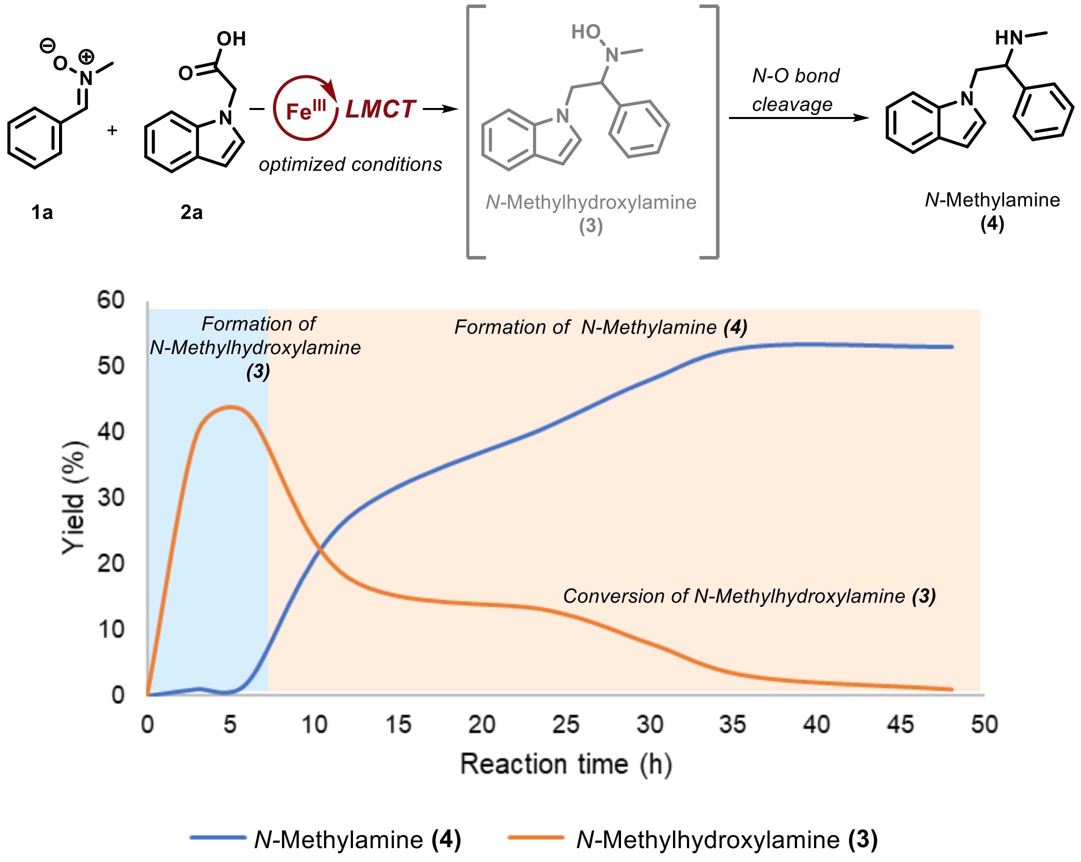

Scheme S3. Time-course analysis of the *in situ* formation of *N*-methylhydroxylamine (**3**) and its conversion into *N*-methylamine (**4**).

7.2 Trapping experiment

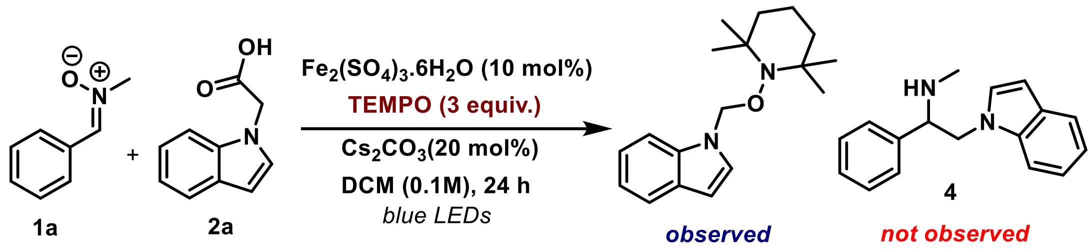

The radical-trapping experiment was carried out by adding TEMPO (2,2,6,6-tetramethylpiperidin-1-yl)oxyl, 3.0 equiv.) to the standard reaction mixture under the optimized conditions. In a borosilicate glass Schlenk tube equipped with a magnetic stir bar, nitrone (**1a**, 0.15 mmol), the corresponding carboxylic acid (**2a**, 0.225 mmol, 1.5 equiv.), the Fe(III) catalyst (10 mol%, 0.015 mmol) and Cs_2CO_3 (20 mol%, 0.030 mmol) were added. DCM was then introduced to reach a final concentration of 0.10 M (1.5 mL for 0.15 mmol of substrate). The solution was degassed three times using the freeze-pump-thaw method and irradiated with a blue Kessil H150 LED lamp (34 W, $\lambda_{max} = 456$ nm) under stirring. After 24 h, the solvent was removed under reduced pressure, and the crude mixture was analyzed by MS. In the presence of TEMPO, no desired product was detected, supporting the involvement of a radical pathway.

An aliquot was taken from the crude reaction mixture and prepared in 1% HCOOH/MeOH, then analyzed by mass spectrometry using an ACQUITY UPC2-MS system via direct infusion. The full-scan MS experiment indicated that the peak at *m/z* 287.19 corresponds to the coupling of the radical scavenger with the alkyl radical derived from the carboxylic acid, as shown in figure S1.

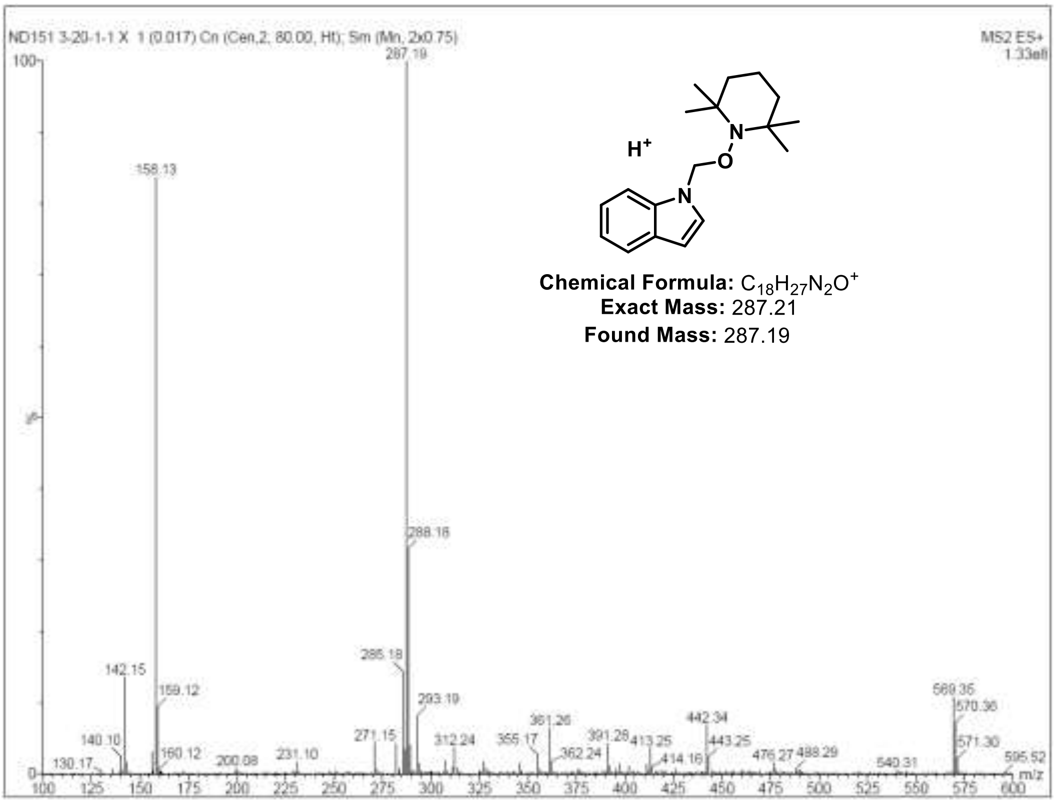

Figure S1. Full-scan MS experiment via direct infusion of the crude reaction mixture. The exact mass of the compound is reported as the $[M+H]^+$ adduct.

7.3 Mass spectrum of the crude reaction mixture

The reaction was performed following the general procedure. An aliquot was taken from the crude reaction mixture and prepared in 1% HCOOH/MeOH, then analyzed by mass spectrometry using an ACQUITY UPC2-MS system via direct infusion. The full-scan mass spectrometry analysis revealed a peak at *m/z* 251.13 corresponding to product **4**, and another at *m/z* 136.04 attributed to the unreacted nitrone, as shown in figure S2.

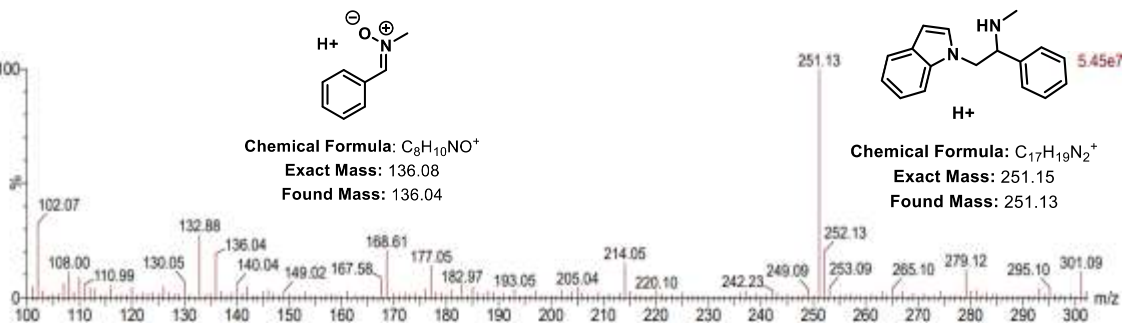

Figure S2. Full-scan MS experiment via direct infusion of the crude reaction mixture. The exact mass of the compound is reported as the [M+H]+ adduct.

7.4 Cyclic voltammetry measurements

The electrochemical measurement by cyclic voltammetry was performed in a potentiostat/galvanostat model Autolab PGSTAT-204 (EcoChemie, Netherlands) controlled by the NOVA 2.1.2 software. A conventional three-electrode system was employed, with a platinum plate as the auxiliary electrode. The reference electrode was Ag/AgCl/KCl (3.0 mol L^{-1}), and the glassy carbon electrode (GCE) was used as the working electrode. The GCE surface was polished with 1.0 and 0.5 µm alumina slurry and washed with deionised water after all measurements. A solution of Bu_4NPF_6 (0.10 mol L^{-1}) in degassed MeCN was used with electrolyte, one containing hydroxylamine (**3**) (Figure S4) and the other $FeCl_3$ (0.10 mmol·L^{-1}) (Figure S3). A potential window was scanned from −2.0 to +2.0 V at a potential scan rate of 50 mV s^{-1}, and three cycles were obtained during the potential sweep to ensure measurement accuracy. All voltammetric measurements were performed at room temperature. Cyclic voltammograms were plotted with peak current (I_p) *versus* peak potential (E_p) based on the Ag/AgCl reference electrode (+0.210 V at 25°C).

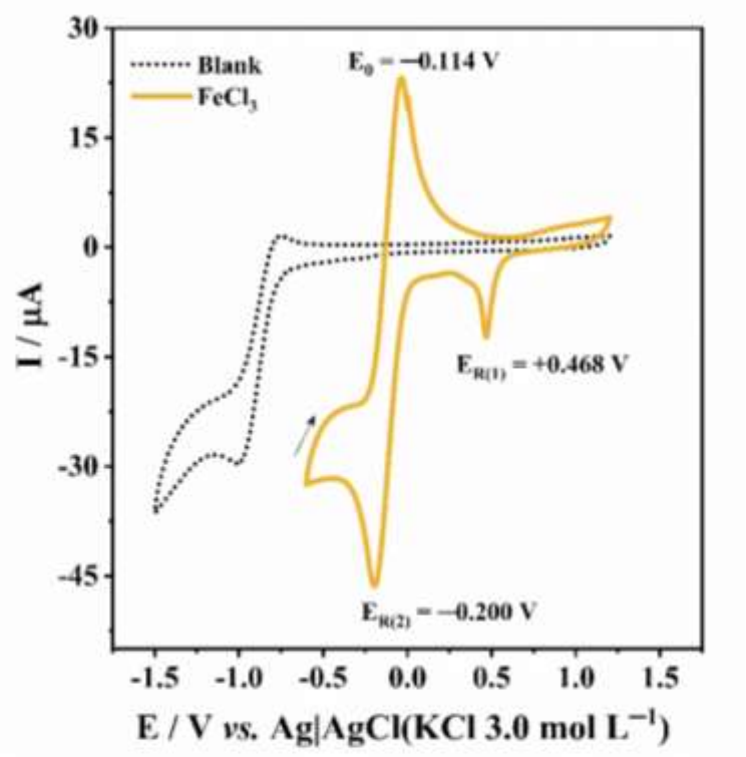

Figure S3. Cyclic voltammetry measurements for $FeCl_3$. The symbol (→) shows the direction of the cyclic voltammetry (cathodic → anodic).

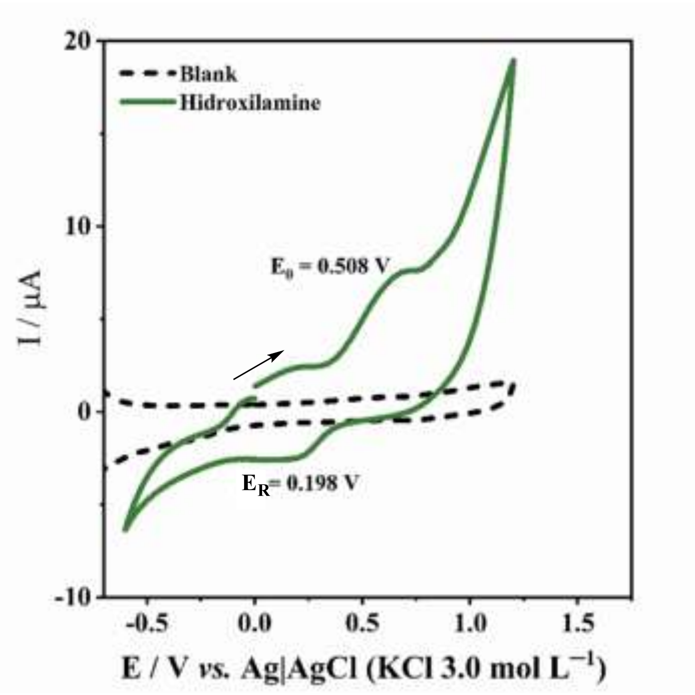

Figure S4. Cyclic voltammetry measurements for hydroxylamine (**3**). The symbol (→) shows the direction of the cyclic voltammetry (cathodic → anodic).

7.5 UV/Vis absorption spectroscopy

Experimental details: UV/Vis absorption spectra were recorded using a 1.0 cm quartz cuvette on a Shimadzu UV-2550 spectrophotometer, operated with a resolution of up to 0.1 nm over a spectral range of 190–500 nm. The absorption spectra of the individual reaction components and their mixtures were measured in DCM solution (0.1 mM).

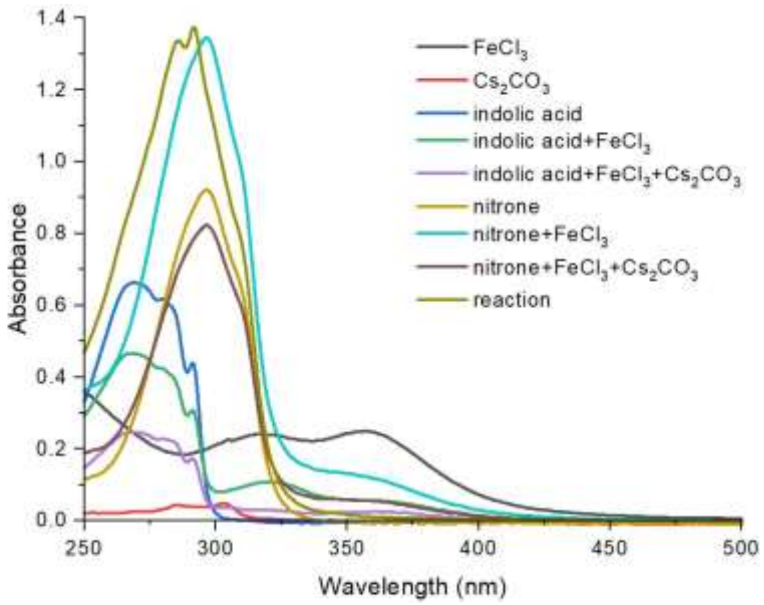

Figure S5: UV–Vis spectra of the individual components and their combination.

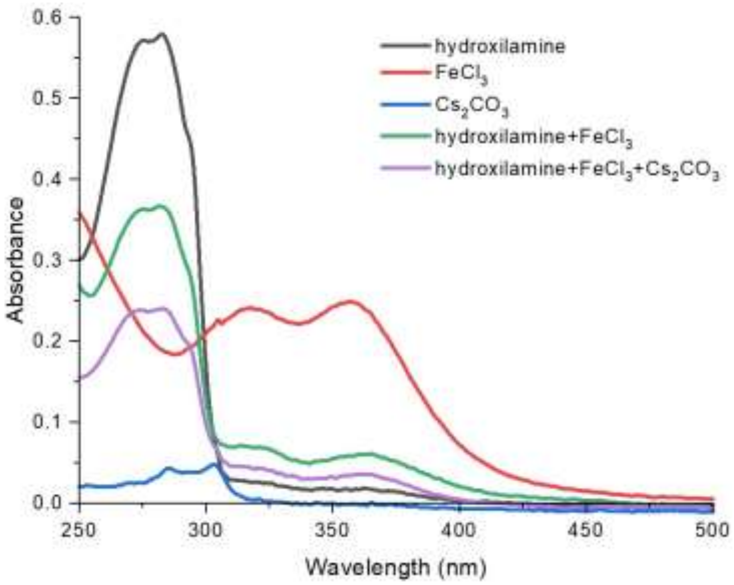

Figure S6: UV–Vis spectra of the individual components and their combination.

Visual evidence: A color change was observed in the solutions containing carboxylic acid 2a + $FeCl_3$ (e) and hydroxylamine 3 + $FeCl_3$ (h) at a concentration of 0.1 M. In addition, the nitrone + $FeCl_3$ solution (d) also showed a color change. This can be attributed to the formation of a coordination complex between the reagents.

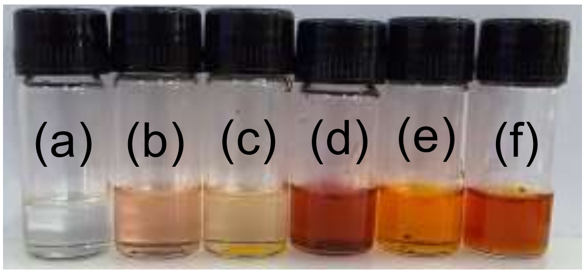

Figure S7. Visual evidence of the formations of coordination complex between **2a** and $FeCl_3$. nitrone **1a** (a), carboxylic acid **2a** (b), $FeCl_3$ (c), nitrone **1a** + $FeCl_3$ (d), carboxylic acid **2a** + $FeCl_3$ (e), and the complete reaction mixture (f) in dichloromethane.

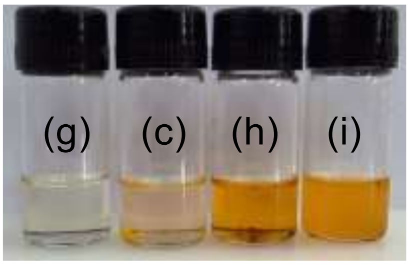

Figure S8. Visual evidence of the formations of coordination complex between **3** and $FeCl_3$. hydroxylamine **3** (g), $FeCl_3$ (c), hydroxylamine **3** + $FeCl_3$ (h), and hydroxylamine **3** + $FeCl_3$+ Na_2CO_3 (e) in dichloromethane.

7.6 Investigation of the reduction of *N*-methylhydroxylamines into *N*-methylamines

To investigate which reaction conditions influence the reduction of *N*-methylhydroxylamine to *N*-methylamine, we initially synthesized compound **3** (Table S2). This compound was then subjected to the reaction conditions using $Fe_2(SO_4)_3{\cdot}6H_2O$ (10 mol%) in dichloromethane (0.1 M) under blue light irradiation (λ_{max} = 456 nm) positioned 4 cm from the reaction vessel at room temperature for 24 hours. Under these conditions, product **4** was obtained in 59% yield (entry, Table S2). The addition of 20 mol% and 1 equivalent of Cs_2CO_3 (entry 2, Table S2) resulted only in trace amounts of product 4, suggesting that the presence of base inhibits the transformation. Based on the UV–Vis spectra, 390 nm was selected as the most suitable wavelength for absorption of the catalytic system. However, even under these conditions (entry 3, Table S2), in the presence of 20 mol% of Cs_2CO_3, only traces of **4** were observed. To evaluate the impact of a proton source, 30 mol% of acetic acid was added to the reaction, which led to a decrease in yield to 30% (entry 4, Table S2). Moreover, a control experiment performed in the absence of light did not afford any 4 (entry 5, Table S2), reinforcing the photocatalytic nature of the transformation.

Table S2. Investigation of the reaction conditions for the reduction of *N*-methylhydroxylamine (**3**) to *N*-methylamine (**4**).

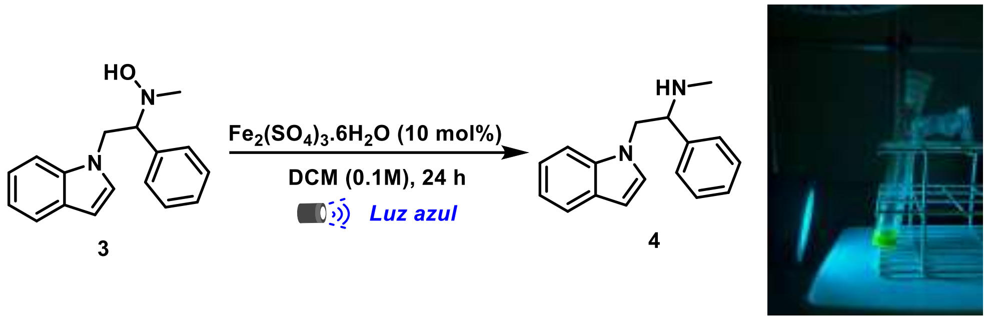

Entry	Conditions	Yield 4 %
1	none	59
2	Cs_2CO_3 (20 mol%)	trace
3	390 nm, Cs_2CO_3 (20 mol%)	trace
4	30% AcOH	30
5	absence of light	n.d

8 Compound characterization data

8.1 Starting materials

(*Z*)-*N*-methyl-1-((3a*R*,5*R*,5a*S*,8a*S*,8b*R*)-2,2,7,7-tetramethyltetrahydro-5*H*-bis([1,3]dioxolo)[4,5-*b*:4',5'-*d*]pyran-5-yl)methanimine oxide (18r)

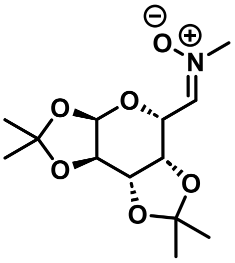

(263 mg, 46% yield), yellowish viscous oil, purified by flash column chromatography (20−50% EtOAc in hexane). **^{1}H NMR (400 MHz, CDCl$_3$)** δ 6.74 (d, J = 5.2 Hz, 1H), 5.51 (d, J = 5.0 Hz, 1H), 5.02 (d, J = 5.2 Hz, 1H), 4.68 (dd, J = 7.9, 1.8 Hz, 1H), 4.62 (dd, J = 7.9, 2.4 Hz, 1H), 4.31 (dd, J = 5.0, 2.4 Hz, 1H), 3.69 (s, 3H), 1.56 (s, 3H), 1.42 (s, 3H), 1.31 (s, 3H), 1.30 (s, 3H) ppm. **^{13}C NMR (101 MHz, CDCl$_3$)** δ 137.2, 109.5, 109.2, 96.2, 70.5, 70.0, 65.2, 52.6, 26.2, 26.0, 25.0, 24.2 ppm. **HRMS** (ESI) $[M + H]^+$ *m/z*: calculated for $C_{13}H_{22}NO_6$ 288.1441, found: 288.1439.

(*Z*)-1-((3a*R*,4*R*,6*R*,6a*R*)-6-methoxy-2,2-dimethyltetrahydrofuro[3,4-*d*][1,3]dioxol-4-yl)-*N*-methylmethanimine oxide (19s)

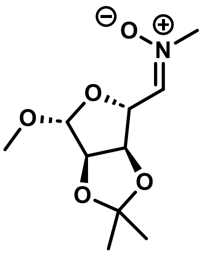

(208 mg, 44% yield), colorless oil, purified by flash column chromatography (20−50% EtOAc in Hexane). **^{1}H NMR (400 MHz, CDCl$_3$)** δ 6.73 (d, J = 5.3 Hz, 1H), 5.24 (d, J = 5.6 Hz, 1H), 5.02 (s, 1H), 4.83 (dd, J = 6.0, 0.9 Hz, 1H), 4.52 (d, J = 5.9 Hz, 1H), 3.70 (s, 3H), 3.39 (s, 3H), 1.49 (s, 3H), 1.31 (s, 3H) ppm. **^{13}C NMR (101 MHz, CDCl$_3$)** δ 139.1, 112.8, 110.5, 84.4, 82.6, 82.4, 55.4, 52.5, 26.4, 25.0 ppm. **HRMS** (ESI) $[M + H]^+$ *m/z*: calculated for $C_{10}H_{18}NO_5$ 232.1179, found: 232.1178.

8.2 Scope for the nitrones

2-(1*H*-indol-1-yl)-*N*-methyl-1-phenylethan-1-amine (4)

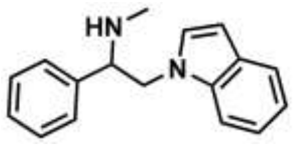

The product **4** was prepared according to general process and was purified by flash chromatography on silica gel (EtOAc/hexane 40/60%) to afford a yellow oil (23 mg, 61% yield). **^{1}H NMR (400 MHz, CDCl$_3$)** δ 7.56 (dt, J = 7.8, 1.1 Hz, 1H), 7.34 (dq, J = 8.2, 0.9 Hz, 1H), 7.32 – 7.20 (m, 5H), 7.14 (ddd, J = 8.2, 7.0, 1.2 Hz, 1H), 7.05 (ddd, J = 8.0, 7.1, 1.0 Hz, 1H), 6.92 (d, J = 3.2 Hz, 1H), 6.39 (dd, J = 3.2, 0.9 Hz, 1H), 4.41 – 4.14 (m, 2H), 3.97 (dd, J = 8.3, 5.2 Hz, 1H), 2.14 (s, 3H), 2.10 (s, 1H) ppm. **^{13}C NMR (101 MHz, CDCl$_3$)** δ 140.3, 136.2, 128.9, 128.8, 128.4, 128.1, 127.5, 121.8, 121.1, 119.7, 109.6, 101.7, 65.2, 53.5, 34.4 ppm. **HRMS** (ESI) $[M + H]^+$ *m/z*: calculated for $C_{17}H_{19}N_2$ 251.1542, found: 251.1541.

1-(4-fluorophenyl)-2-(1*H*-indol-1-yl)-*N*-methylethan-1-amine (5)

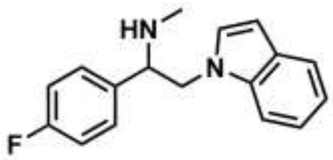

The product **5** was prepared according to general process and was purified by flash chromatography on silica gel (EtOAc/hexane 40/60%) to afford a yellow oil (21 mg, 52% yield). **^{1}H NMR (400 MHz, CDCl$_3$)** δ 7.56 (dt, J = 7.8, 1.0 Hz, 1H), 7.31 (dd, J = 8.2, 0.9 Hz, 1H), 7.25 – 7.20 (m, 2H), 7.15 (ddd, J = 8.2, 7.0, 1.2 Hz, 1H), 7.05 (ddd, J = 8.0, 7.0, 1.0 Hz, 1H), 6.98 – 6.94 (m, 2H), 6.89 (d, J = 3.2 Hz, 1H), 6.39 (dd, J = 3.1, 0.9 Hz, 1H), 4.24 – 4.11 (m, 1H), 3.94 (dd, J = 8.3, 5.3 Hz, 1H), 2.12 (s, 3H), 1.81 (s, 1H) ppm. **^{13}C NMR (101 MHz, CDCl$_3$)** δ 163.7, 161.3 (d, J = 246.0 Hz), 136.2 (d, J = 8.0 Hz), 129.0, 128.9, 128.8, 128.3, 121.8, 121.2 (d, J = 21.2 Hz), 119.7, 115.8, 115.6, 109.5, 101.8, 64.6, 53.6, 34.4 ppm. **^{19}F NMR (376 MHz, CDCl$_3$)** δ -114.42 ppm. **HRMS** (ESI) $[M + H]^+$ *m/z*: calculated for $C_{17}H_{18}FN_2$ 269.1448, found: 269.1447.

2-(1*H*-indol-1-yl)-*N*-methyl-1-(4-(trifluoromethyl)phenyl)ethan-1-amine (6)

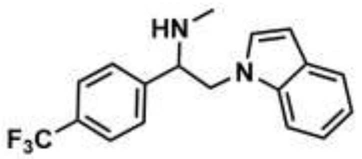

The product **6** was prepared according to general process and was purified by flash chromatography on silica gel (EtOAc/hexane 30/70%) to afford a yellow oil (20 mg, 43% yield). **^{1}H NMR (400 MHz, CDCl$_3$)** δ 7.61 – 7.48 (m, 3H), 7.42 – 7.33 (m, 2H), 7.29 (dd, *J* = 8.2, 0.9 Hz, 1H), 7.15 (ddd, *J* = 8.3, 7.0, 1.1 Hz, 1H), 7.05 (ddd, *J* = 8.0, 7.0, 1.0 Hz, 1H), 6.89 (d, *J* = 3.1 Hz, 1H), 6.41 (dd, *J* = 3.1, 0.9 Hz, 1H), 4.24 – 4.10 (m, 2H), 4.02 (dd, *J* = 8.0, 5.4 Hz, 1H), 2.12 (s, 3H), 1.73 (s, 1H) ppm. **^{13}C NMR (101 MHz, CDCl$_3$)** *δ* 144.8, 136.2, 130.4, 130.1 (d, *J* = 32.5 Hz), 128.8, 128.2, 127.9, 125.8, 125.8, 125.8, 125.7, 125.5 (q, *J* = 3.7 Hz), 122.8, 121.9 (d, *J* = 272.1 Hz), 121.2, 119.8, 109.4, 102.0, 65.1, 53.4, 34.5 ppm. **^{19}F NMR (376 MHz, CDCl$_3$)** *δ* -62.54 ppm. **HRMS** (ESI) $[M + H]^+$ *m/z*: calculated for $C_{18}H_{18}F_3N_2$ 319.1416, found: 319.1414.

1-([1,1'-biphenyl]-4-yl)-2-(1*H*-indol-1-yl)-*N*-methylethan-1-amine (7)

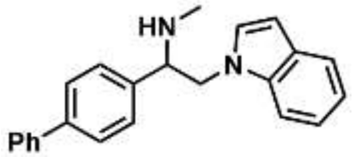

The product **7** was prepared according to general process and was purified by flash chromatography on silica gel (EtOAc/hexane 40/60%) to afford a yellow oil (27 mg, 55% yield). **^{1}H NMR (400 MHz, CDCl$_3$)** *δ* 7.62 – 7.47 (m, 5H), 7.36 (q, *J* = 7.3 Hz, 5H), 7.28 (t, *J* = 7.1 Hz, 1H), 7.16 (dd, *J* = 9.0, 6.1 Hz, 1H), 7.05 (t, *J* = 7.5 Hz, 1H), 6.96 (d, *J* = 3.2 Hz, 1H), 6.41 (d, *J* = 3.1 Hz, 1H), 4.23 (d, *J* = 6.7 Hz, 2H), 4.01 (t, *J* = 6.7 Hz, 1H), 2.16 (s, 3H), 1.96 (s, 1H) ppm. **^{13}C NMR (101 MHz, CDCl$_3$)** *δ* 140.9, 140.8, 139.5, 136.3, 128.9, 128.8, 128.5, 127.9, 127.6, 127.4, 127.2, 121.8, 121.1, 119.7, 109.6, 101.7, 65.0, 53.6, 34.6 ppm. **HRMS** (ESI) $[M + H]^+$ *m/z*: calculated for $C_{23}H_{23}N_2$ 327.1855, found: 327.1854.

2-(1*H*-indol-1-yl)-1-(4-methoxyphenyl)-*N*-methylethan-1-amine (8)

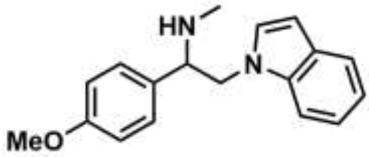

The product **8** was prepared according to general process and was purified by flash chromatography on silica gel (EtOAc/hexane 40/60%) to afford a yellow oil (21 mg, 49% yield). **^{1}H NMR (400 MHz, CDCl$_3$)** *δ* 7.55 (dt, *J* = 7.9, 1.0 Hz, 1H), 7.39 – 7.31 (m, 1H), 7.19 (d, *J* = 8.5 Hz, 2H), 7.15 (ddd, *J* = 8.3, 7.0, 1.2 Hz, 1H), 7.04 (ddd, *J* = 8.0, 7.0, 1.0 Hz, 1H), 6.90 (d, *J* = 3.2 Hz, 1H), 6.82 (d, *J* = 8.7 Hz, 2H), 6.38 (dd, *J* = 3.2, 0.8 Hz, 1H), 4.26 (dd, *J* = 14.3, 8.4 Hz, 1H), 4.18 (dd, *J* = 14.3, 5.2 Hz, 1H), 3.93 (dd, *J* = 8.3, 5.2 Hz, 1H), 3.73 (s, 3H), 2.14 (s, 3H) ppm. **^{13}C NMR (101 MHz, CDCl$_3$)** *δ* 159.5, 136.2,

128.8, 128.6, 128.5, 121.8, 121.1, 119.6, 114.3, 109.6, 101.6, 64.5, 55.4, 53.4, 34.2 ppm. **HRMS** (ESI) $[M + H]^+$ *m/z*: calculated for $C_{18}H_{21}N_2O$ 281.1648, found: 281.1647.

2-(1*H*-indol-1-yl)-*N*-methyl-1-(2-((3-methylbut-2-en-1-yl)oxy)phenyl)ethan-1-amine (9)

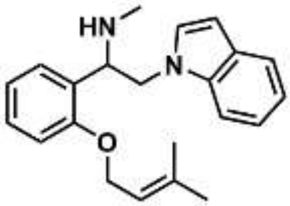

The product **9** was prepared according to general process and was purified by flash chromatography on silica gel (EtOAc/hexane 20/80%) to afford a yellow oil (28 mg, 56% yield). **^{1}H NMR (400 MHz, CDCl$_3$)** δ 7.58 – 7.51 (m, 1H), 7.51 (dq, J = 8.3, 0.9 Hz, 1H), 7.29 (dd, J = 7.5, 1.8 Hz, 1H), 7.20 – 7.15 (m, 1H), 7.12 (ddd, J = 8.2, 7.0, 1.3 Hz, 1H), 7.03 (ddd, J = 8.0, 7.0, 1.1 Hz, 1H), 6.97 (d, J = 3.1 Hz, 1H), 6.91 – 6.84 (m, 2H), 6.38 (dd, J = 3.1, 0.9 Hz, 1H), 5.52 (tdd, J = 5.4, 2.9, 1.4 Hz, 1H), 4.61 – 4.44 (m, 2H), 4.42 – 4.29 (m, 2H), 3.99 (dd, J = 15.0, 9.7 Hz, 1H), 2.10 (s, 3H), 1.80 (d, J = 1.4 Hz, 3H), 1.70 (d, J = 1.4 Hz, 3H) ppm. **^{13}C NMR (101 MHz, CDCl$_3$)** δ 157.0, 138.6, 136.3, 128.8, 128.7, 128.4, 128.3, 127.8, 121.5, 120.9, 120.8, 119.7, 119.5, 111.7, 110.2, 101.2, 65.0, 59.3, 52.1, 34.6, 26.0, 18.4 ppm. **HRMS** (ESI) $[M + H]^+$ *m/z*: calculated for $C_{22}H_{27}N_2O$ 335.2117, found: 335.2115.

2-(1*H*-indol-1-yl)-1-(4-isobutylphenyl)-*N*-methylethan-1-amine (10)

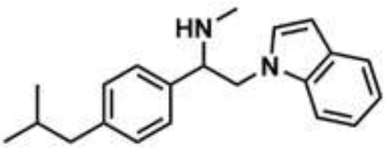

The product **10** was prepared according to general process and was purified by flash chromatography on silica gel (EtOAc/hexane 40/60%) to afford a yellow oil (23 mg, 50% yield). **^{1}H NMR (400 MHz, CDCl$_3$)** δ 7.55 (dt, J = 7.8, 1.0 Hz, 1H), 7.31 (dd, J = 8.3, 1.0 Hz, 1H), 7.21 – 7.10 (m, 3H), 7.08 – 7.00 (m, 3H), 6.93 (d, J = 3.2 Hz, 1H), 6.39 (dd, J = 3.1, 0.8 Hz, 1H), 4.20 (dd, J = 6.8, 4.3 Hz, 2H), 3.92 (dd, J = 8.0, 5.4 Hz, 1H), 2.40 (d, J = 7.2 Hz, 2H), 2.13 (s, 3H), 1.80 (dq, J = 13.5, 6.8 Hz, 2H), 0.83 (d, J = 6.7 Hz, 6H) ppm. **^{13}C NMR (101 MHz, CDCl$_3$)** δ 141.5, 137.6, 136.3, 129.6, 128.7, 128.5, 127.2, 121.7, 121.1, 119.6, 109.6, 101.6, 65.0, 53.6, 45.2, 34.5, 30.3, 22.5, 22.5 ppm. **HRMS** (ESI) $[M + H]^+$ *m/z*: calculated for $C_{21}H_{27}N_2$ 307.2168, found: 307.2167.

2-(1*H*-indol-1-yl)-1-(4-iodophenyl)-*N*-methylethan-1-amine (11)

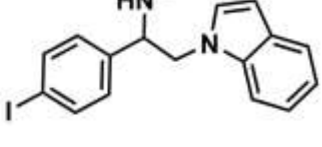

The product **11** was prepared according to general process and was purified by flash chromatography on silica gel (EtOAc/hexane 30/70%) to afford a yellow oil (34 mg, 60%

yield). **^{1}H NMR (400 MHz, $CDCl_3$)** δ 7.65 – 7.59 (m, 2H), 7.56 (dt, J = 7.8, 1.0 Hz, 1H), 7.36 – 7.27 (m, 1H), 7.16 (dt, J = 8.2, 1.1 Hz, 1H), 7.09 – 6.97 (m, 3H), 6.89 (d, J = 3.2 Hz, 1H), 6.40 (dd, J = 3.1, 0.9 Hz, 1H), 4.33 – 4.05 (m, 2H), 3.91 (dd, J = 8.0, 5.5 Hz, 1H), 2.11 (s, 3H), 1.71 (s, 1H) ppm. **^{13}C NMR (101 MHz, $CDCl_3$)** δ 140.3, 137.9, 136.2, 129.5, 128.8, 128.3, 121.9, 121.2, 119.8, 109.5, 101.9, 93.4, 64.9, 53.4, 34.5 ppm. **HRMS** (ESI) $[M + H]^+$ m/z: calculated for $C_{17}H_{18}IN_2$ 377.0509, found: 377.0507.

1-(4-bromophenyl)-2-(1*H*-indol-1-yl)-*N*-methylethan-1-amine (12)

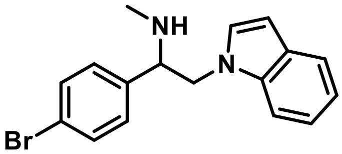

The product **12** was prepared according to general process and was purified by flash chromatography on silica gel (EtOAc/hexane 30/70%) to afford a yellow oil (25 mg, 50% yield). **^{1}H NMR (400 MHz, $CDCl_3$)** δ 7.61 – 7.51 (m, 1H), 7.48 – 7.35 (m, 2H), 7.29 (dd, J = 8.2, 0.9 Hz, 1H), 7.19 – 7.09 (m, 3H), 7.04 (ddd, J = 8.0, 7.1, 1.0 Hz, 1H), 6.87 (d, J = 3.2 Hz, 1H), 6.39 (dd, J = 3.1, 0.9 Hz, 1H), 4.35 – 4.01 (m, 2H), 3.90 (dd, J = 8.1, 5.4 Hz, 1H), 2.09 (s, 3H), 1.83 (s, 1H). **^{13}C NMR (101 MHz, $CDCl_3$)** δ 139.6, 136.2, 131.9, 129.2, 128.8, 128.3, 121.9, 121.7, 121.2, 119.7, 109.5, 101.8, 64.7, 53.4, 34.5 ppm. **HRMS** (ESI) $[M + H]^+$ m/z: calculated for $C_{17}H_{18}BrN_2$ 329.0647, found: 329.0645.

1-(2-chlorophenyl)-2-(1*H*-indol-1-yl)-*N*-methylethan-1-amine (13)

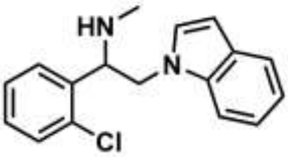

The product **13** was prepared according to general process and was purified by flash chromatography on silica gel (EtOAc/hexane 20/80%) to afford a yellow oil (24 mg, 56% yield). **^{1}H NMR (400 MHz, $CDCl_3$)** δ 7.64 (d, J = 7.8 Hz, 1H), 7.60 – 7.49 (m, 2H), 7.42 (d, J = 7.9 Hz, 1H), 7.30 (t, J = 7.4 Hz, 1H), 7.25 – 7.20 (m, 2H), 7.12 (dd, J = 8.6, 5.4 Hz, 2H), 6.52 (d, J = 3.2 Hz, 1H), 4.61 (dd, J = 9.3, 3.7 Hz, 1H), 4.41 (dd, J = 14.4, 3.8 Hz, 1H), 4.08 (dd, J = 14.4, 9.3 Hz, 1H), 1.25 (s, 4H) ppm. **^{13}C NMR (101 MHz, $CDCl_3$)** δ 137.4, 136.5, 134.0, 129.9, 128.9, 128.8, 128.5, 128.4, 127.5, 121.9, 121.1, 119.7, 109.7, 101.9, 61.3, 51.8, 34.5, 29.8 ppm. **HRMS** (ESI) $[M + H]^+$ m/z: calculated for $C_{17}H_{18}ClN_2$ 285.1153, found: 285.1151.

1-(3-chlorophenyl)-2-(1*H*-indol-1-yl)-*N*-methylethan-1-amine (14)

The product **14** was prepared according to general process and was purified by flash chromatography on silica gel (EtOAc/hexane 30/70%) to afford a yellow oil (25 mg, 58%

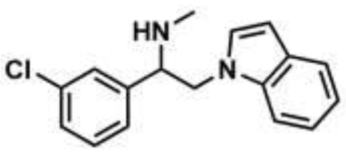

yield). **^{1}H NMR (400 MHz, $CDCl_3$)** δ 7.64 (d, J = 7.9 Hz, 1H), 7.46 – 7.36 (m, 2H), 7.32 – 7.18 (m, 4H), 7.13 (t, J = 7.5 Hz, 1H), 7.00 (d, J = 3.2 Hz, 1H), 6.49 (d, J = 3.2 Hz, 1H), 4.22 (d, J = 6.7 Hz, 2H), 4.00 (t, J = 6.8 Hz, 1H), 2.18 (s, 3H), 1.71 (s, 1H) ppm. **^{13}C NMR (101 MHz, $CDCl_3$)** δ 143.0, 136.2, 134.8, 130.1, 128.8, 128.3, 128.2, 127.5, 125.8, 121.9, 121.2, 119.8, 109.5, 101.9, 65.0, 53.5, 34.6 ppm. **HRMS** (ESI) $[M + H]^+$ *m/z*: calculated for $C_{17}H_{18}ClN_2$ 285.1153, found: 285.1152.

1-(4-chlorophenyl)-2-(1*H*-indol-1-yl)-*N*-methylethan-1-amine (15)

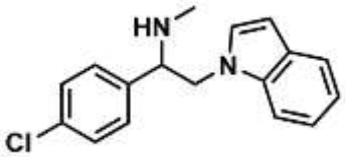

The product **15** was prepared according to general process and was purified by flash chromatography on silica gel (EtOAc/hexane 40/60%) to afford a yellow oil (26 mg, 61% yield). **^{1}H NMR (400 MHz, $CDCl_3$)** δ 7.56 (d, J = 7.8 Hz, 1H), 7.31 (d, J = 8.2 Hz, 1H), 7.27 – 7.13 (m, 5H), 7.09 – 7.03 (m, 1H), 6.89 (d, J = 3.1 Hz, 1H), 6.47 – 6.34 (m, 1H), 4.22 – 4.10 (m, 2H), 3.95 (dd, J = 8.2, 5.3 Hz, 1H), 2.12 (s, 3H), 1.76 (s, 1H) ppm. **^{13}C NMR (101 MHz, $CDCl_3$)** δ 139.0, 136.2, 133.7, 129.0, 128.8, 128.8, 128.3, 121.9, 121.2, 119.8, 109.5, 101.8, 64.7, 53.4, 34.4 ppm. **HRMS** (ESI) $[M + H]^+$ *m/z*: calculated for $C_{17}H_{18}ClN_2$ 285.1153, found: 285.1151.

2-(1*H*-indol-1-yl)-1-(6-methoxynaphthalen-2-yl)-*N*-methylethan-1-amine (16)

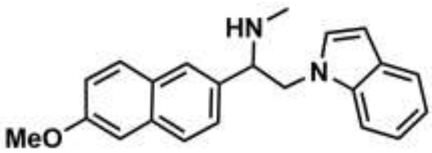

The product **16** was prepared according to general process and was purified by flash chromatography on silica gel (EtOAc/hexane 40/60%) to afford a yellow oil (21 mg, 43% yield). **^{1}H NMR (400 MHz, $CDCl_3$)** δ 7.69 (d, J = 8.5 Hz, 1H), 7.68 – 7.61 (m, 2H), 7.57 (dt, J = 7.9, 1.0 Hz, 1H), 7.41 (ddd, J = 8.5, 2.3, 1.4 Hz, 2H), 7.23 – 7.13 (m, 1H), 7.11 – 7.03 (m, 3H), 6.93 (d, J = 3.1 Hz, 1H), 6.39 (dd, J = 3.2, 0.9 Hz, 1H), 4.38 – 4.20 (m, 2H), 4.09 (dd, J = 8.3, 5.1 Hz, 1H), 3.85 (s, 3H), 2.15 (s, 3H), 1.93 (s, 1H) ppm. **^{13}C NMR (101 MHz, $CDCl_3$)** δ 157.9, 136.3, 135.4, 134.5, 129.4, 129.0, 128.8, 128.5, 127.6, 126.6, 125.6, 121.8, 121.2, 119.7, 119.2, 109.6, 105.8, 101.7, 65.3, 55.4, 53.6, 34.5 ppm. **HRMS** (ESI) $[M + H]^+$ *m/z*: calculated for $C_{22}H_{23}N_2O$ 331.1805, found: 331.1804.

1-(furan-2-yl)-2-(1*H*-indol-1-yl)-*N*-methylethan-1-amine (17)

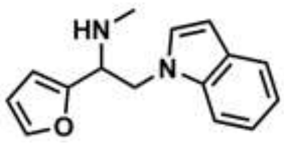

The product **17** was prepared according to general process and was purified by flash chromatography on silica gel (EtOAc/hexane 40/60%) to afford a yellow oil (19 mg, 53% yield). **^{1}H NMR (400 MHz, $CDCl_3$)** δ 7.61 (d, J = 7.8 Hz, 1H), 7.47 – 7.40 (m, 1H), 7.35 (d, J = 8.2 Hz, 1H), 7.20 (t, J = 7.6 Hz, 1H), 7.10 (t, J = 7.4 Hz, 1H), 6.96 (d, J = 3.2 Hz, 1H), 6.45 (d, J = 3.1 Hz, 1H), 6.31 (dd, J = 3.2, 1.8 Hz, 1H), 6.15 (d, J = 3.2 Hz, 1H), 4.43 (dd, J = 6.9, 2.5 Hz, 2H), 4.08 (t, J = 6.7 Hz, 1H), 2.29 (s, 3H), 1.66 (s, 1H) ppm. **^{13}C NMR (101 MHz, $CDCl_3$)** δ 152.9, 142.3, 136.2, 128.7, 128.4, 121.7, 121.1, 119.6, 110.4, 109.4, 108.5, 101.7, 58.4, 50.3, 34.2 ppm. **HRMS** (ESI) $[M + H]^+$ *m/z*: calculated for $C_{15}H_{17}N_2O$ 241.1335, found: 241.1334.

2-(1*H*-indol-1-yl)-*N*-methyl-1-(thiophen-2-yl)ethan-1-amine (18)

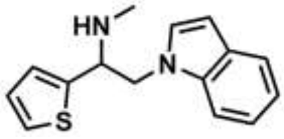

The product **18** was prepared according to general process and was purified by flash chromatography on silica gel (EtOAc/hexane 20/80%) to afford a yellow oil (23 mg, 60% yield). **^{1}H NMR (400 MHz, $CDCl_3$)** δ 7.56 (dt, J = 7.9, 1.0 Hz, 1H), 7.32 (dd, J = 8.3, 1.0 Hz, 1H), 7.24 – 7.11 (m, 2H), 7.05 (ddd, J = 8.0, 7.0, 1.0 Hz, 1H), 6.95 (d, J = 3.2 Hz, 1H), 6.88 (dd, J = 5.0, 3.5 Hz, 1H), 6.83 (dd, J = 3.5, 1.3 Hz, 1H), 6.41 (dd, J = 3.1, 0.9 Hz, 1H), 4.36 – 4.18 (m, 3H), 2.21 (s, 3H), 1.75 (s, 1H) ppm. **^{13}C NMR (101 MHz, $CDCl_3$)** δ 144.9, 136.2, 128.8, 128.4, 126.9, 125.4, 125.0, 121.9, 121.2, 119.7, 109.5, 101.8, 60.9, 54.0, 34.5 ppm. **HRMS** (ESI) $[M + H]^+$ *m/z*: calculated for $C_{15}H_{17}N_2S$ 257.1107, found: 257.1105.

1-cyclohexyl-2-(1*H*-indol-1-yl)-*N*-methylethan-1-amine (19)

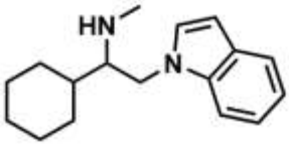

The product **19** was prepared according to general process and was purified by flash chromatography on silica gel (EtOAc/hexane 40/60%) to afford a yellow oil (19 mg, 47% yield). **^{1}H NMR (400 MHz, $CDCl_3$)** δ 7.56 (dt, J = 7.9, 1.0 Hz, 1H), 7.28 (dd, J = 8.2, 1.1 Hz, 1H), 7.13 (ddd, J = 8.3, 7.0, 1.3 Hz, 1H), 7.08 (d, J = 3.1 Hz, 1H), 7.04 (ddd, J = 8.0, 7.0, 1.0 Hz, 1H), 6.43 (dd, J = 3.1, 0.8 Hz, 1H), 4.15 (dd, J = 14.4, 4.9 Hz, 1H), 4.00 (dd, J = 14.4, 8.5 Hz, 1H), 2.90 – 2.68 (m, 1H), 2.20 (s, 3H), 2.05 (s, 1H), 1.94 – 1.59 (m, 5H), 1.50 (td, J = 9.7, 4.3 Hz, 1H), 1.24 – 1.05 (m, 5H) ppm. **^{13}C NMR (101 MHz, $CDCl_3$)** δ 136.3, 128.8, 128.6, 121.6, 121.2, 119.5, 109.5, 101.5, 64.4, 47.7, 39.2, 34.9,

29.0, 28.7, 26.7, 26.6 ppm. **HRMS** (ESI) $[M + H]^+$ *m/z*: calculated for $C_{17}H_{25}N_2$ 257.2012, found: 257.2010.

1-cyclopentyl-2-(1*H*-indol-1-yl)-*N*-methylethan-1-amine (20)

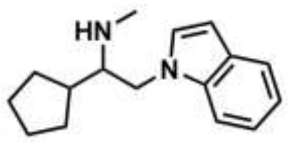

The product **20** was prepared according to general process and was purified by flash chromatography on silica gel (EtOAc/hexane 40/60%) to afford a yellow oil (19 mg, 50% yield). **^{1}H NMR (400 MHz, CDCl$_3$)** δ 7.56 (dt, J = 7.9, 1.0 Hz, 1H), 7.33 – 7.26 (m, 1H), 7.18 – 7.08 (m, 2H), 7.03 (ddd, J = 8.0, 7.0, 1.0 Hz, 1H), 6.43 (dd, J = 3.1, 0.9 Hz, 1H), 4.12 (dd, J = 14.4, 4.8 Hz, 1H), 4.04 (dd, J = 14.4, 7.9 Hz, 1H), 2.87 – 2.70 (m, 1H), 2.21 (s, 3H), 1.95 – 1.83 (m, 1H), 1.82 – 1.68 (m, 2H), 1.65 – 1.53 (m, 2H), 1.53 – 1.44 (m, 2H), 1.38 – 1.24 (m, 2H) ppm. **^{13}C NMR (101 MHz, CDCl$_3$)** δ 136.4, 128.7, 128.6, 121.5, 121.1, 119.4, 109.5, 101.4, 63.8, 48.8, 42.0, 34.3, 29.7, 29.1, 25.8, 25.6 ppm. **HRMS** (ESI) $[M + H]^+$ *m/z*: calculated for $C_{16}H_{23}N_2$ 243.1855, found: 243.1855.

2-(1*H*-indol-1-yl)-*N*-methyl-1-((3a*R*,5*R*,5a*S*,8a*S*,8b*R*)-2,2,7,7-tetramethyltetrahydro-5*H*-bis([1,3]dioxolo)[4,5-*b*:4',5'-*d*]pyran-5-yl)ethan-1-amine (21)

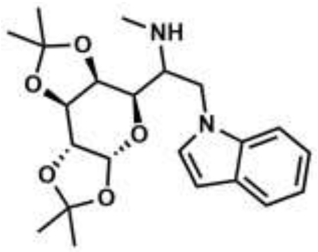

The product **21** was prepared according to general process and was purified by flash chromatography on silica gel (EtOAc/hexane 30/70%) to afford a yellow oil (28 mg, 47% yield). dr = >20:1 based on ^{1}H NMR of the isolated mixture. **^{1}H NMR (400 MHz, CDCl$_3$)** δ 7.54 (dt, J = 7.9, 1.0 Hz, 1H), 7.39 (dd, J = 8.3, 1.0 Hz, 1H), 7.28 (d, J = 3.2 Hz, 1H), 7.13 (ddd, J = 8.3, 7.0, 1.3 Hz, 1H), 7.02 (ddd, J = 7.9, 7.0, 1.0 Hz, 1H), 6.42 (dd, J = 3.2, 0.8 Hz, 1H), 5.56 (d, J = 5.1 Hz, 1H), 4.60 – 4.51 (m, 2H), 4.38 (dd, J = 7.9, 1.9 Hz, 1H), 4.27 (dd, J = 5.1, 2.3 Hz, 1H), 4.18 (dd, J = 14.8, 6.9 Hz, 1H), 3.64 (dd, J = 7.6, 1.9 Hz, 1H), 3.17 (td, J = 7.2, 3.5 Hz, 1H), 2.34 (s, 3H), 1.43 (s, 3H), 1.41 (s, 3H), 1.31 (s, 3H), 1.27 (s, 3H) ppm. **^{13}C NMR (101 MHz, CDCl$_3$)** δ 136.9, 129.4, 128.6, 121.6, 121.0, 119.4, 109.5, 109.3, 108.7, 101.6, 96.8, 71.6, 71.0, 70.7, 67.3, 59.1, 45.0, 33.9, 26.2, 26.0, 25.0, 24.6 ppm. **HRMS** (ESI) $[M + H]^+$ *m/z*: calculated for $C_{22}H_{31}N_2O_5$ 403.2227, found: 403.2225. *The low-intensity signals observed in the spectrum correspond to the minor diastereomer, whose peaks partially overlap with those of the major diastereomer.

2-(1*H*-indol-1-yl)-1-((3a*R*,4*R*,6*R*,6a*R*)-6-methoxy-2,2-dimethyltetrahydrofuro[3,4-*d*][1,3]dioxol-4-yl)-*N*-methylethan-1-amine (22)

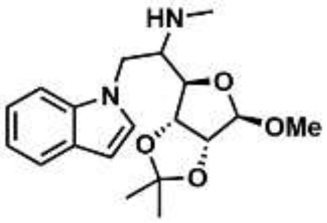

The product **22** was prepared according to general process and was purified by flash chromatography on silica gel (EtOAc/hexane 30/70%) to afford a yellow oil (28 mg, 54% yield). dr = 6:1 based on ^{1}H NMR of the isolated mixture. **^{1}H NMR (400 MHz, CDCl$_3$)** δ 7.55 (ddt, *J* = 7.8, 3.0, 1.0 Hz, 1H), 7.41 – 7.35 (m, 1H), 7.17 – 6.99 (m, 3H), 6.46 (dt, *J* = 3.1, 1.2 Hz, 1H), 5.55 (s, 1H), 4.98 (s, 1H), 4.85 (dd, *J* = 6.0, 1.6 Hz, 1H), 4.53 (d, *J* = 6.1 Hz, 1H), 4.36 (dd, *J* = 14.8, 4.5 Hz, 1H), 4.22 (dd, *J* = 14.8, 5.3 Hz, 1H), 3.84 (dd, *J* = 9.5, 1.6 Hz, 1H), 3.42 (s, 3H), 2.80 (dt, *J* = 9.6, 4.9 Hz, 1H), 2.32 (s, 3H), 1.38 (s, 3H), 1.24 (s, 3H) ppm. **^{13}C NMR (101 MHz, CDCl$_3$)** δ 137.0, 129.1, 128.6, 121.8, 121.0, 119.6, 112.7, 110.1, 109.3, 103.0, 102.0, 87.4, 85.1, 82.1, 70.0, 61.8, 56.1, 45.2, 34.3, 26.6, 25.1 ppm. **HRMS** (ESI) [M + H]$^+$ *m/z*: calculated for $C_{19}H_{27}N_2O_4$ 347.1965, found: 347.1963.

8.3 Scope for the carboxylic acids

2-(6-bromo-1*H*-indol-1-yl)-*N*-methyl-1-phenylethan-1-amine (23)

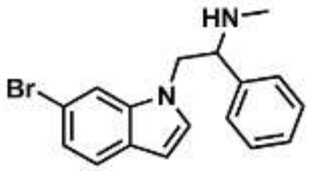

The product **23** was prepared according to general process and was purified by flash chromatography on silica gel (EtOAc/hexane 40/60%) to afford a yellow oil (28 mg, 57% yield).**^{1}H NMR (400 MHz, CDCl$_3$)** δ 7.68 – 7.63 (m, 1H), 7.31 – 7.11 (m, 7H), 6.88 (d, *J* = 3.2 Hz, 1H), 6.31 (dd, *J* = 3.1, 0.8 Hz, 1H), δ 4.21 (dd, *J* = 14.3, 8.0 Hz, 1H), 4.14 (dd, *J* = 14.3, 5.4 Hz, 1H), 3.90 (dd, *J* = 8.0, 5.4 Hz, 1H), 2.15 (s, 3H), 1.93 (s, 1H) ppm. **^{13}C NMR (101 MHz, CDCl$_3$)** δ 140.2, 135.0, 130.4, 129.6, 128.9, 128.1, 127.4, 124.6, 123.5, 112.9, 111.0, 101.2, 65.2, 53.6, 34.5 ppm. **HRMS** (ESI) [M + H]$^+$ *m/z*: calculated for $C_{17}H_{18}BrN_2$ 329.0647, found: 329.0646.

2-(9*H*-carbazol-9-yl)-*N*-methyl-1-phenylethan-1-amine (24)

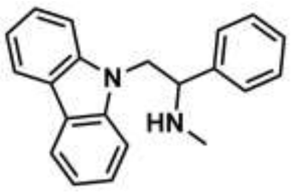

The product **24** was prepared according to general process and was purified by flash chromatography on silica gel (EtOAc/hexane 30/70%) to afford a light-yellow oil (19 mg, 42% yield). **^{1}H NMR (400 MHz, CDCl$_3$)** δ 8.01 (dt, *J* = 7.7, 1.0 Hz, 2H), 7.39 – 7.36 (m, 6H), 7.33 – 7.13 (m, 5H), 4.54 (dd, *J* = 14.8, 8.5 Hz, 1H), 4.36 (dd,

J = 14.8, 5.1 Hz, 1H), 4.16 (dd, J = 8.5, 5.1 Hz, 1H), 2.14 (s, 3H) ppm. **^{13}C NMR (101 MHz, $CDCl_3$)** δ 140.7, 129.0, 128.2, 127.6, 125.9, 123.1, 120.4, 119.3, 109.1, 64.8, 50.4, 34.3 ppm. **HRMS** (ESI) $[M + H]^+$ *m/z*: calculated for $C_{21}H_{21}N_2$ 301.1699, found: 301.1697.

2-(4-bromophenyl)-*N*-methyl-1-phenylethan-1-amine (25)

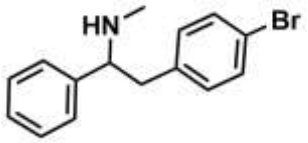

The product **25** was prepared according to general process and was purified by flash chromatography on silica gel (EtOAc/hexane 30/70%) to afford a light-yellow oil (18 mg, 42% yield). **^{1}H NMR (400 MHz, $CDCl_3$)** δ 7.30 – 7.17 (m, 8H), 6.94 – 6.77 (m, 2H), 3.88 – 3.66 (m, 2H), 3.14 (dd, J = 13.4, 6.4 Hz, 1H), 2.97 (dd, J = 13.3, 8.0 Hz, 1H), 2.25 (s, 3H) ppm. **^{13}C NMR (101 MHz, $CDCl_3$)** δ 136.7, 131.5, 131.2, 128.8, 128.2, 127.9, 120.6, 66.5, 42.8, 33.3 ppm. **HRMS** (ESI) $[M + H]^+$ *m/z*: calculated for $C_{15}H_{17}BrN$ 290.0538, found: 290.0537.

2-(4-methoxyphenyl)-*N*-methyl-1-phenylethan-1-amine (26)

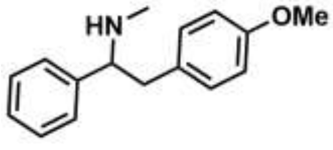

The product **26** was prepared according to general process and was purified by flash chromatography on silica gel (EtOAc/hexane 30/70%) to afford a light-yellow oil (17 mg, 47% yield). **^{1}H NMR (400 MHz, $CDCl_3$)** δ 7.29 – 7.13 (m, 5H), 7.06 – 6.86 (m, 2H), 6.87 – 6.68 (m, 2H), 3.70 (s, 3H), 3.64 (dd, J = 7.6, 6.5 Hz, 1H), 3.19 – 2.77 (m, 2H), 2.25 (s, 1H), 2.16 (s, 3H) ppm. **^{13}C NMR (101 MHz, $CDCl_3$)** δ 158.3, 142.6, 130.6, 130.4, 128.5, 127.6, 127.3, 113.9, 67.1, 55.3, 43.9, 34.4 ppm. **HRMS** (ESI) $[M + H]^+$ *m/z*: calculated for $C_{16}H_{20}NO$ 242.1539, found: 242.1537.

***N*-methyl-1-phenyl-2-(phenylthio)ethan-1-amine (27)**

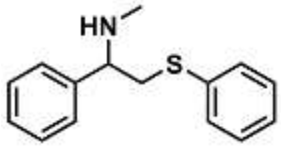

The product **27** was prepared according to general process and was purified by flash chromatography on silica gel (EtOAc/hexane 20/80%) to afford a yellow oil (19 mg, 52% yield). **^{1}H NMR (400 MHz, $CDCl_3$)** δ 7.32 – 7.17 (m, 10H), 3.65 (t, J = 6.9 Hz, 1H), 3.37 (s, 1H), 3.25 (d, J = 6.9 Hz, 2H), 2.25 (s, 3H) ppm. **^{13}C NMR (101 MHz, $CDCl_3$)** δ 135.3, 130.0, 129.9, 129.2, 128.9, 128.2, 128.2, 127.7, 126.6, 63.6, 40.9, 33.7 ppm. **HRMS** (ESI) $[M + H]^+$ *m/z*: calculated for $C_{15}H_{18}NS$ 244.1154, found: 244.1153.

***N*-methyl-1-phenyl-2-(phenylselanyl)ethan-1-amine (28)**

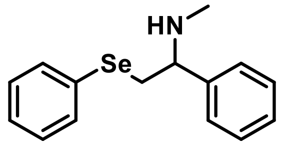

The product **28** was prepared according to general process and was purified by flash chromatography on silica gel (EtOAc/hexane 30/70%) to afford a light-yellow oil (23 mg, 52% yield). **^{1}H NMR (600 MHz, CDCl$_3$)** δ 7.56 – 7.49 (m, 2H), 7.38 – 7.26 (m, 8H), 3.70 (dd, J = 8.6, 5.5 Hz, 1H), 3.31 (dd, J = 12.5, 5.4 Hz, 1H), 3.24 (dd, J = 12.5, 8.6 Hz, 1H), 2.32 (s, 3H), 2.04 (s, 1H) ppm. **^{13}C NMR (151 MHz, CDCl$_3$)** δ 133.0, 129.3, 128.8, 128.0, 127.5, 127.3, 64.4, 35.5, 34.2 ppm. **HRMS** (ESI) $[M + H]^+$ m/z: calculated for $C_{15}H_{18}NSe$ 292.0599, found: 292.0596.

***N*-methyl-1-phenyl-2-(*p*-tolylselanyl)ethan-1-amine (29)**

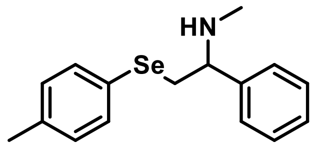

The product **29** was prepared according to general process and was purified by flash chromatography on silica gel (EtOAc/hexane 30/70%) to afford a light-yellow oil (20 mg, 43% yield). **^{1}H NMR (400 MHz, CDCl$_3$)** δ 7.33 (d, J = 7.8 Hz, 2H), 7.30 – 7.15 (m, 5H), 7.01 (d, J = 7.6 Hz, 2H), 3.56 (dd, J = 8.8, 5.2 Hz, 1H), 3.15 (dd, J = 12.4, 5.1 Hz, 1H), 3.06 (dd, J = 12.5, 8.8 Hz, 1H), 2.26 (s, 3H), 2.21 (s, 3H) ppm. **^{13}C NMR (101 MHz, CDCl$_3$)** δ 137.4, 133.5, 130.1, 128.7, 127.9, 127.4, 125.8, 64.3, 36.0, 34.3, 21.2 ppm. **HRMS** (ESI) $[M + H]^+$ m/z: calculated for $C_{16}H_{20}NSe$ 306.0755, found: 306.0753.

2-(butylselanyl)-*N*-methyl-1-phenylethan-1-amine (30)

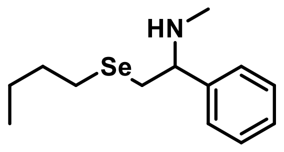

The product **30** was prepared according to general process and was purified by flash chromatography on silica gel (EtOAc/hexane 30/70%) to afford a light-yellow oil (16 mg, 40% yield). **^{1}H NMR (400 MHz, CDCl$_3$)** δ 7.44 – 7.13 (m, 5H), 3.68 (s, 1H), 3.60 (dd, J = 8.5, 5.4 Hz, 1H), 2.85 (dd, J = 12.5, 5.4 Hz, 1H), 2.75 (dd, J = 12.5, 8.4 Hz, 1H), 2.40 (tt, J = 7.4, 3.4 Hz, 2H), 2.24 (s, 3H), 1.62 – 1.42 (m, 2H), 1.27 (h, J = 7.3 Hz, 2H), 0.80 (t, J = 7.3 Hz, 3H) ppm. **^{13}C NMR (101 MHz, CDCl$_3$)** δ 141.9, 128.7, 127.8, 127.4, 64.9, 34.2, 32.7, 31.8, 24.5, 23.0, 13.6 ppm. **HRMS** (ESI) $[M + H]^+$ m/z: calculated for $C_{13}H_{22}NSe$ 272.0912, found: 272.0910.

2-(4-methoxyphenoxy)-*N*-methyl-1-phenylpropan-1-amine (31)

The product **31** was prepared according to general process and was purified by flash chromatography on silica gel (EtOAc/hexane 20/80%) to afford a yellow oil (18 mg, 50%

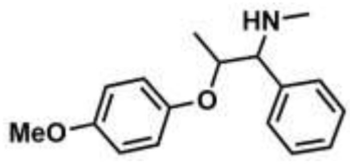

yield). dr = >20:1 based on ^{1}H NMR of the isolated mixture. **^{1}H NMR (400 MHz, CDCl$_3$)** δ 7.36 – 7.26 (m, 5H), 7.24 – 7.20 (m, 1H), 6.92 – 6.80 (m, 2H), 6.79 – 6.72 (m, 2H), 4.44 (qd, J = 6.3, 3.6 Hz, 1H), 3.77 (d, J = 3.5 Hz, 1H), 3.69 (s, 1H), 2.50 (s, 3H), 2.24 (s, 3H), 1.06 (d, J = 6.3 Hz, 3H) ppm. **^{13}C NMR (101 MHz, CDCl$_3$)** δ 154.4, 151.6, 128.5, 128.4, 127.5, 118.1, 114.8, 78.6, 68.0, 55.8, 34.4, 14.8 ppm. **HRMS** (ESI) $[M + H]^+$ *m/z*: calculated for $C_{17}H_{22}NO_2$ 272.1645, found: 272.1642.

(3-(1-(methylamino)-1-phenylpropan-2-yl)phenyl)(phenyl)methanone (32)

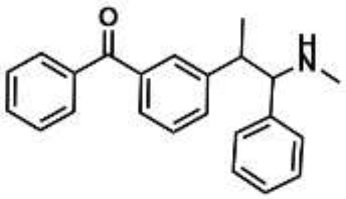

The product **32** was prepared according to general process and was purified by flash chromatography on silica gel (EtOAc/hexane 70/30%) to afford a light-yellow oil (22 mg, 47% yield). **^{1}H NMR (400 MHz, CDCl$_3$)** δ 8.07 – 7.91 (m, 1H), 7.79 – 7.65 (m, 2H), 7.59 – 7.45 (m, 3H), 7.45 – 7.25 (m, 8H), 4.34 (s, 1H), 3.72 (d, J = 9.4 Hz, 1H), 3.27 (dq, J = 9.4, 7.0 Hz, 1H), 2.13 (s, 3H), 0.99 (d, J = 7.0 Hz, 3H) ppm. **^{13}C NMR (101 MHz, CDCl$_3$)** δ 196.8, 144.0, 138.1, 137.6, 132.6, 132.5, 132.0, 130.2, 129.9, 129.2, 129.0, 128.9, 128.8, 128.6, 128.4, 128.3, 128.1, 71.2, 45.7, 33.5, 19.3 ppm. **HRMS** (ESI) $[M + H]^+$ *m/z*: calculated for $C_{23}H_{24}NO$ 330.1852, found: 330.1850.

9 NMR spectra

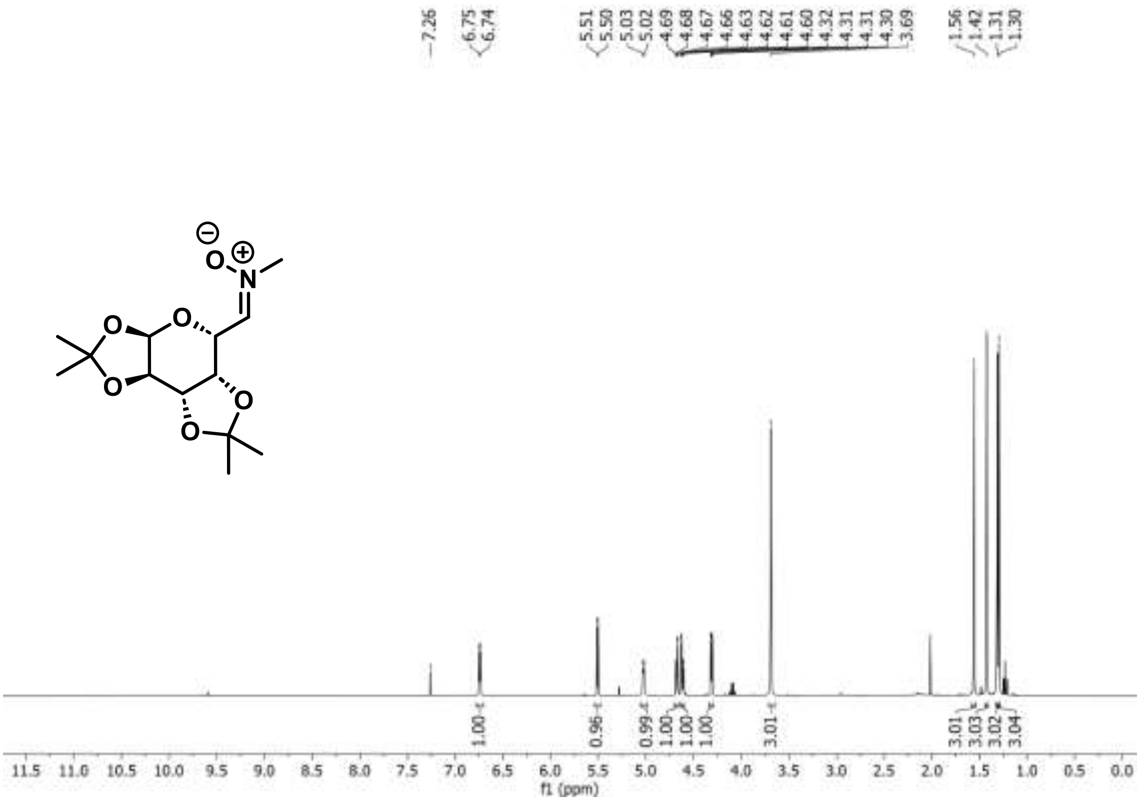

Figure S8. ^{1}H NMR spectrum of **18r** (400 MHz, $CDCl_3$)

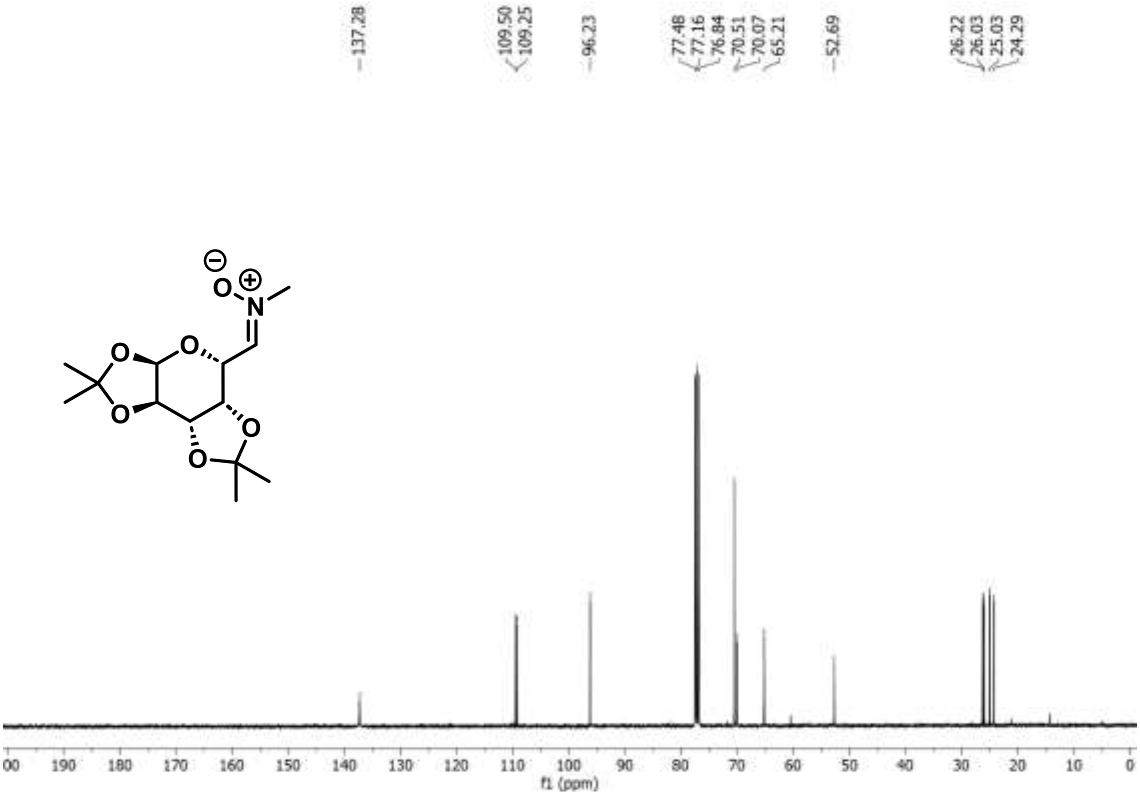

Figure S10. ^{13}C NMR spectrum of **18r** (101 MHz, $CDCl_3$)

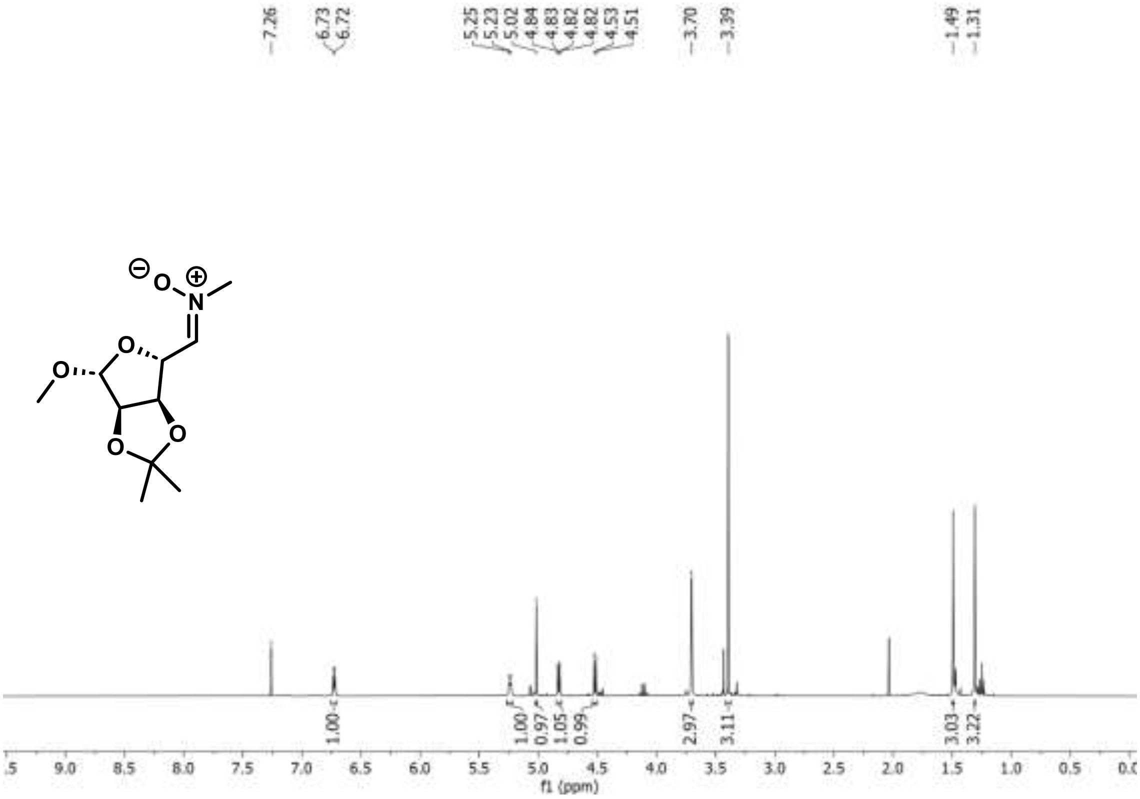

Figure S11. ^{1}H NMR spectrum of **19s** (400 MHz, $CDCl_3$)

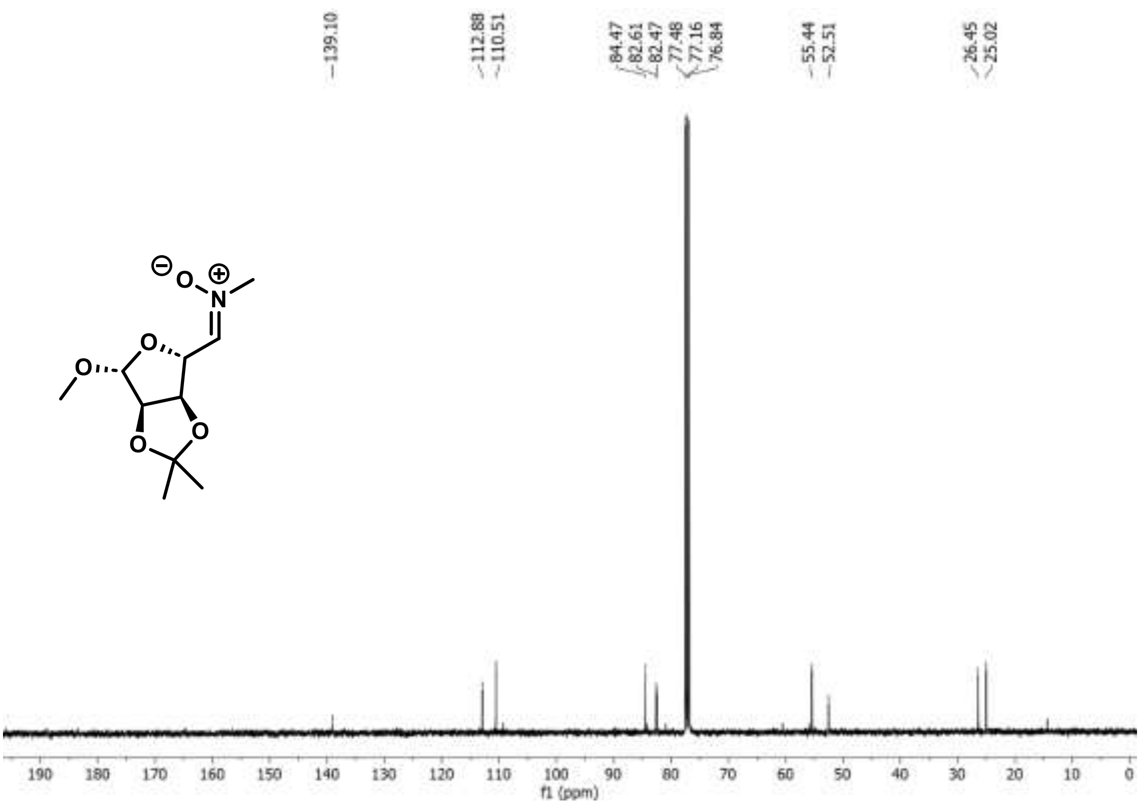

Figure S12. ^{13}C NMR spectrum of **19s** (101 MHz, $CDCl_3$)

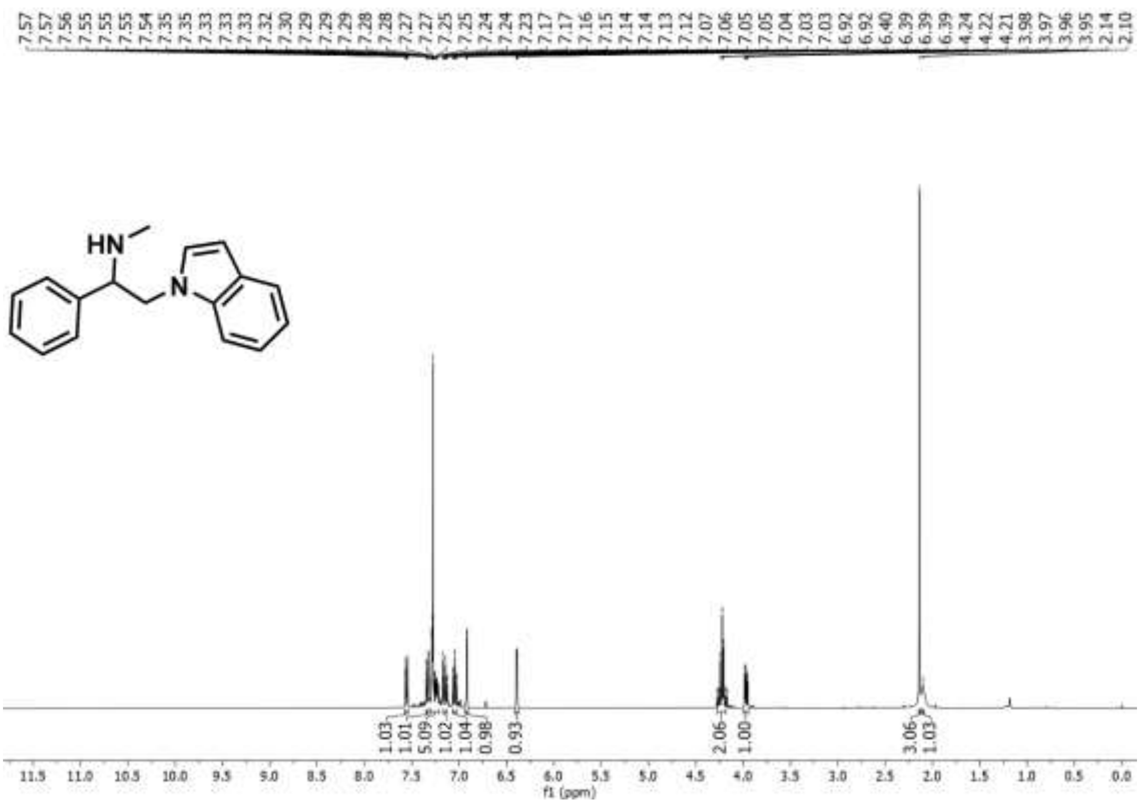

Figure S13. ^{1}H NMR spectrum of **4** (400 MHz, $CDCl_3$)

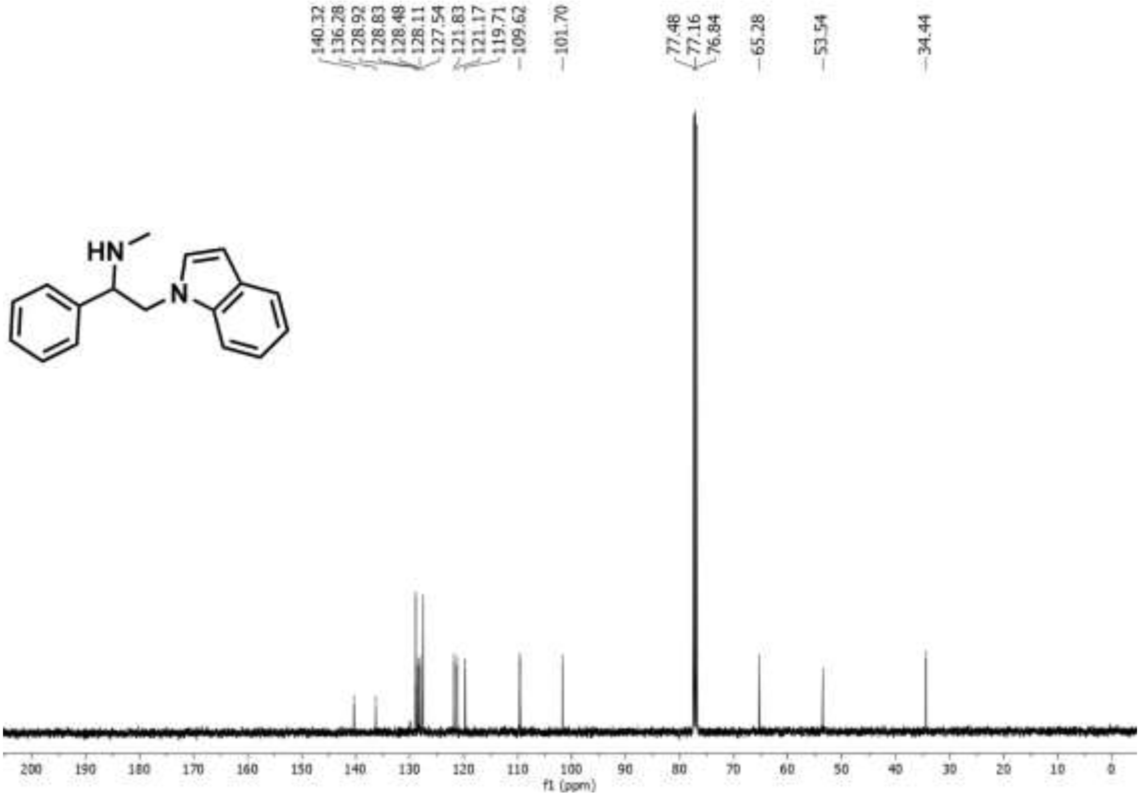

Figure S14. ^{13}C NMR spectrum of **4** (101 MHz, $CDCl_3$)

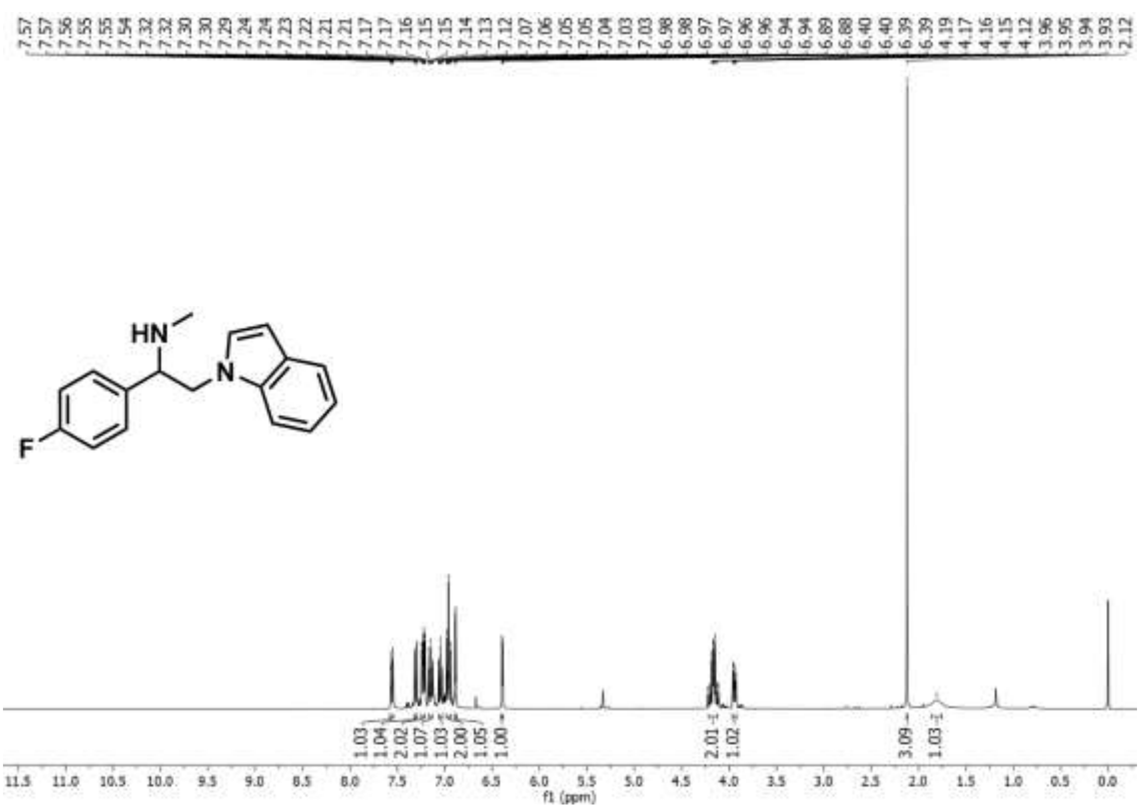

Figure S15. ^{1}H NMR spectrum of **5** (400 MHz, $CDCl_3$)

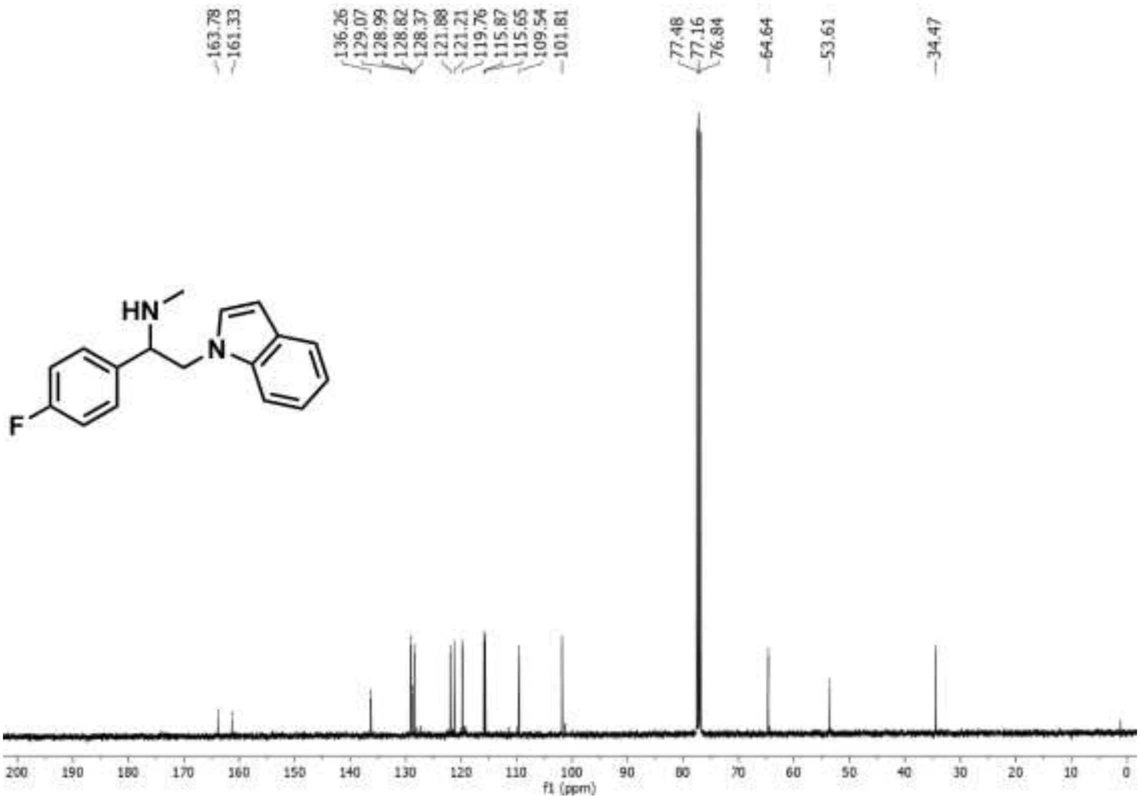

Figure S16. ^{13}C NMR spectrum of **5** (101 MHz, $CDCl_3$)

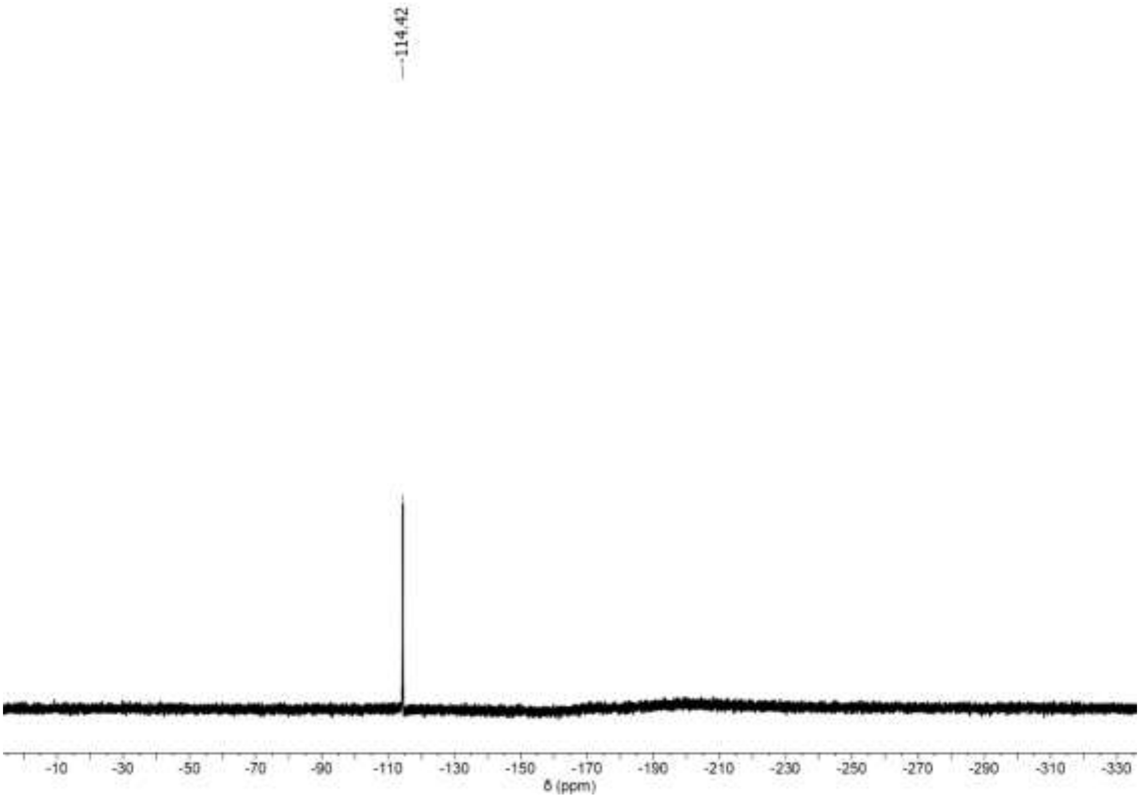

Figure S17. ^{19}F NMR spectrum of **5** (376 MHz, $CDCl_3$)

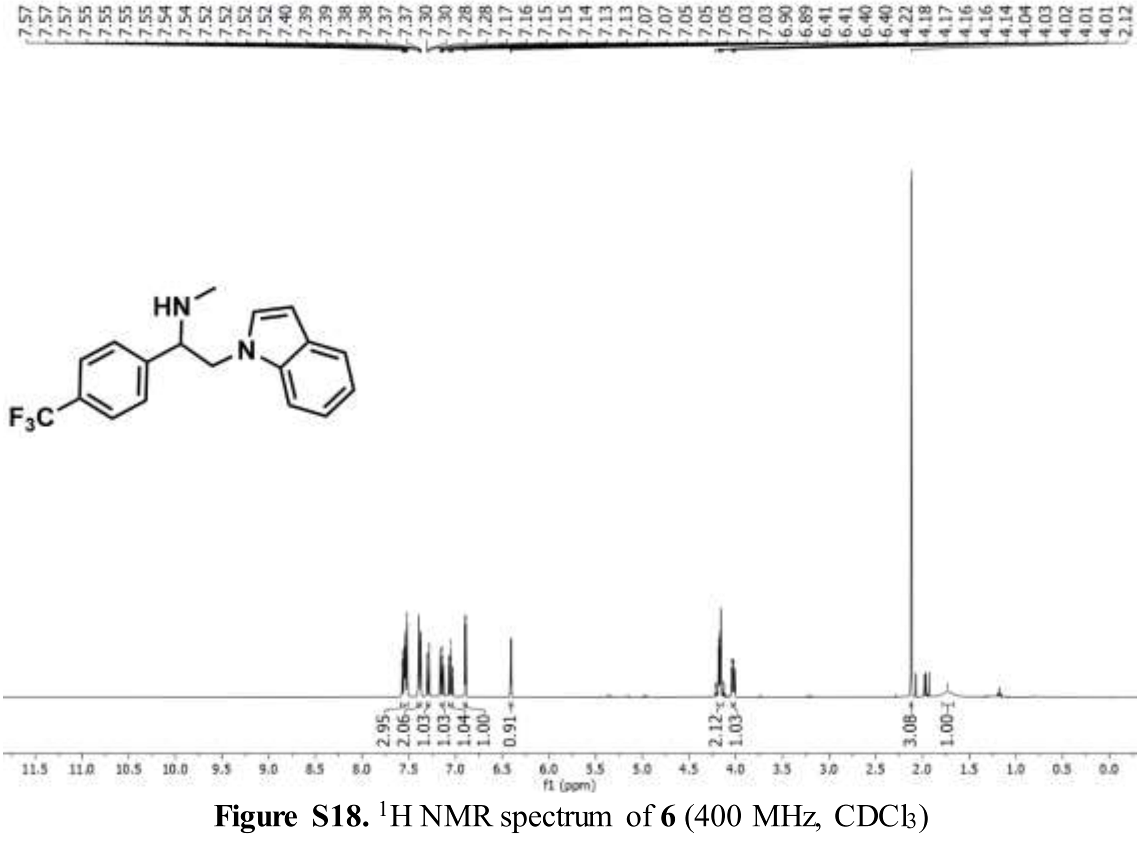

Figure S18. ^{1}H NMR spectrum of **6** (400 MHz, $CDCl_3$)

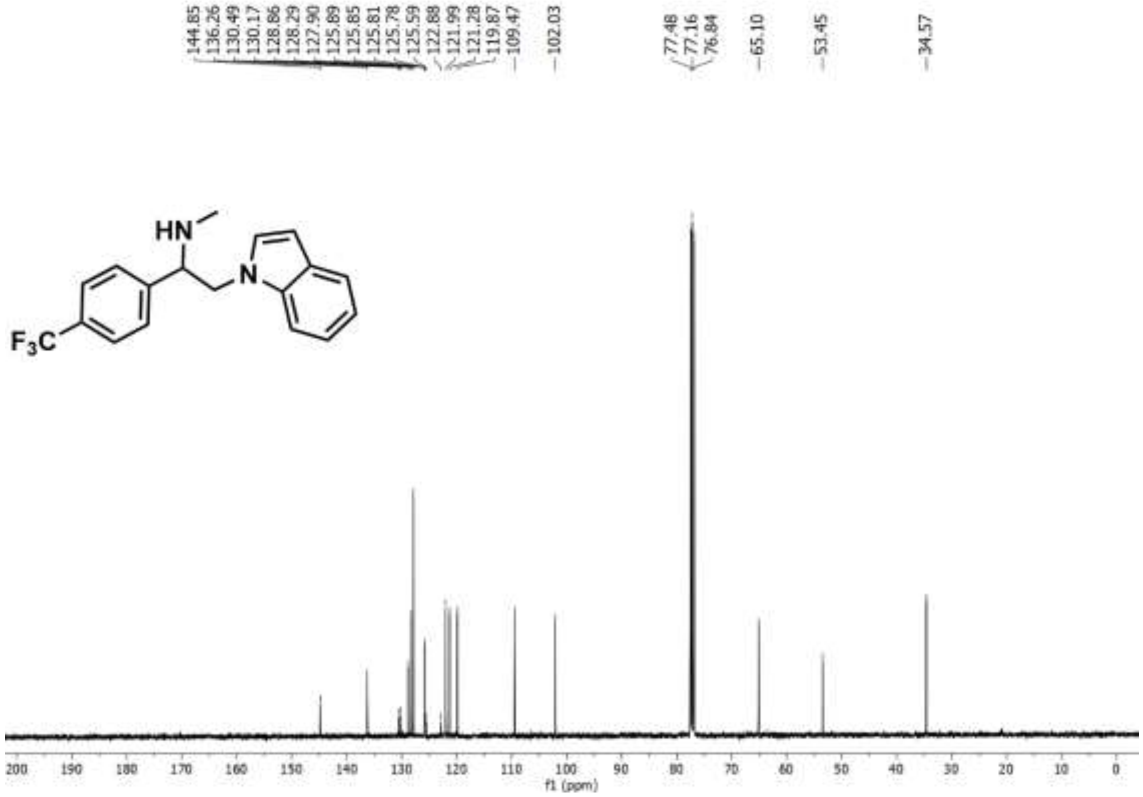

Figure S19. ^{13}C NMR spectrum of **6** (101 MHz, $CDCl_3$)

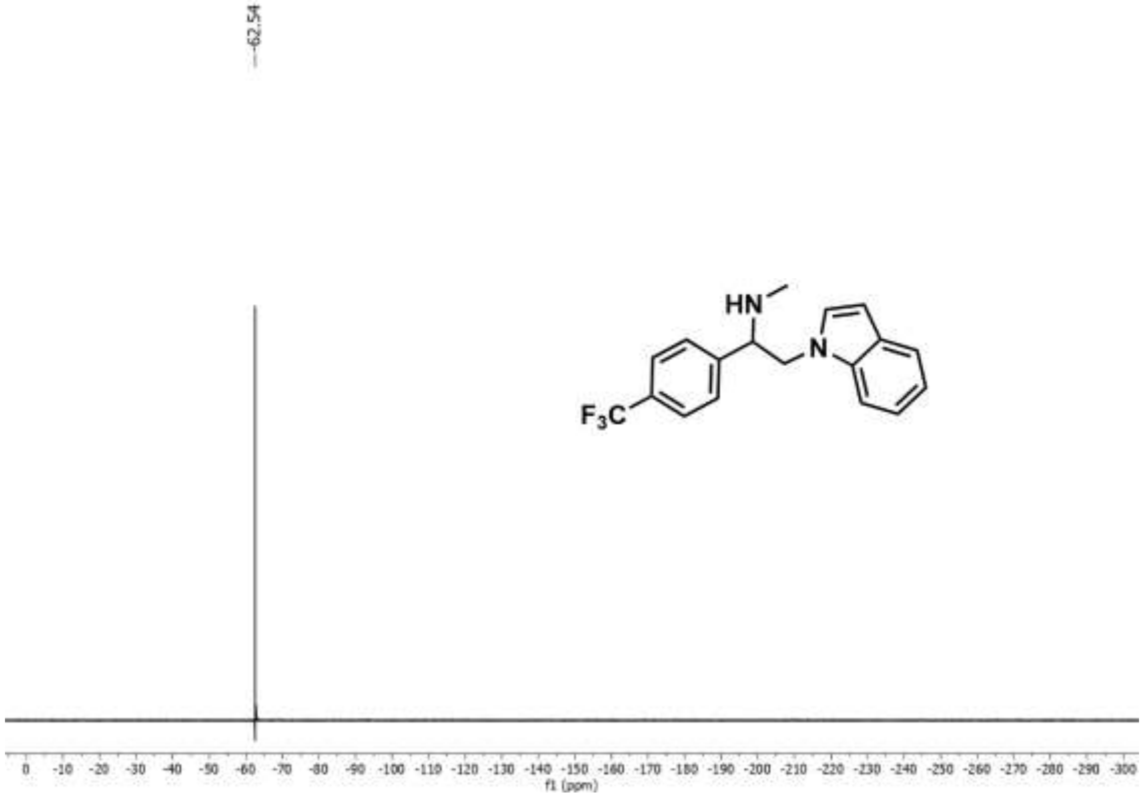

Figure S20. ^{19}F NMR spectrum of **6** (376 MHz, $CDCl_3$)

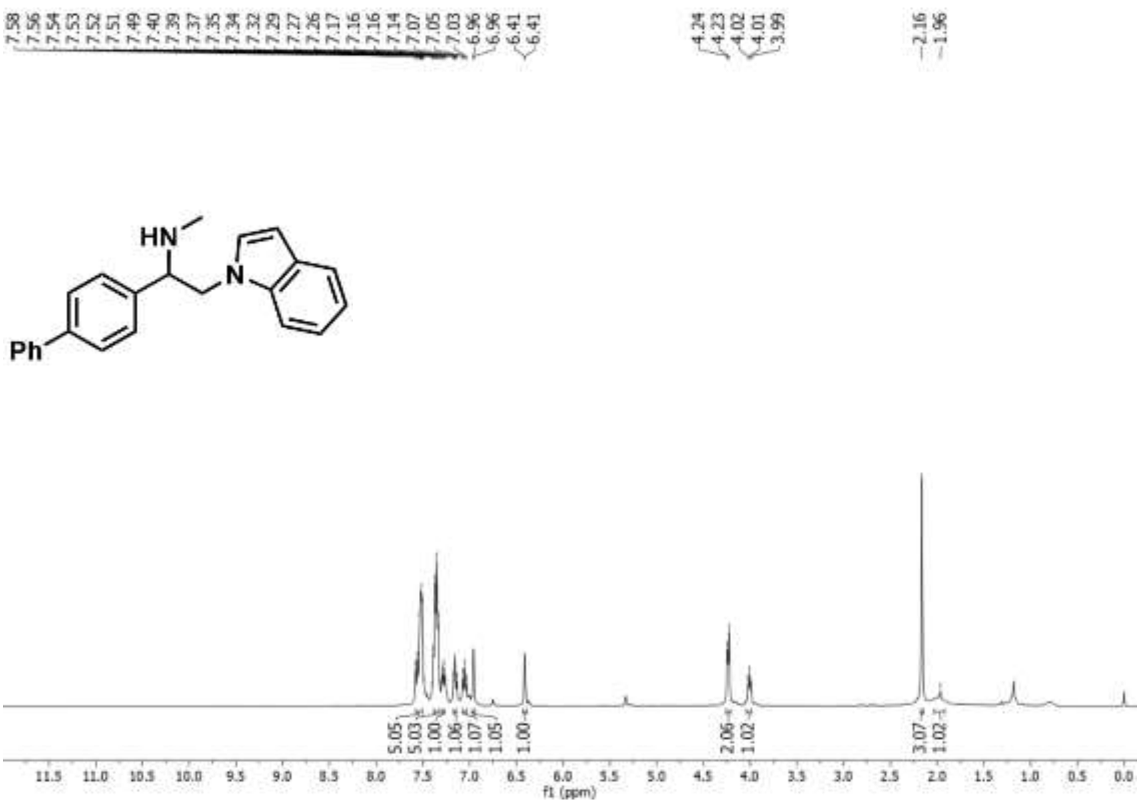

Figure S21. ^{1}H NMR spectrum of **7** (400 MHz, $CDCl_3$)

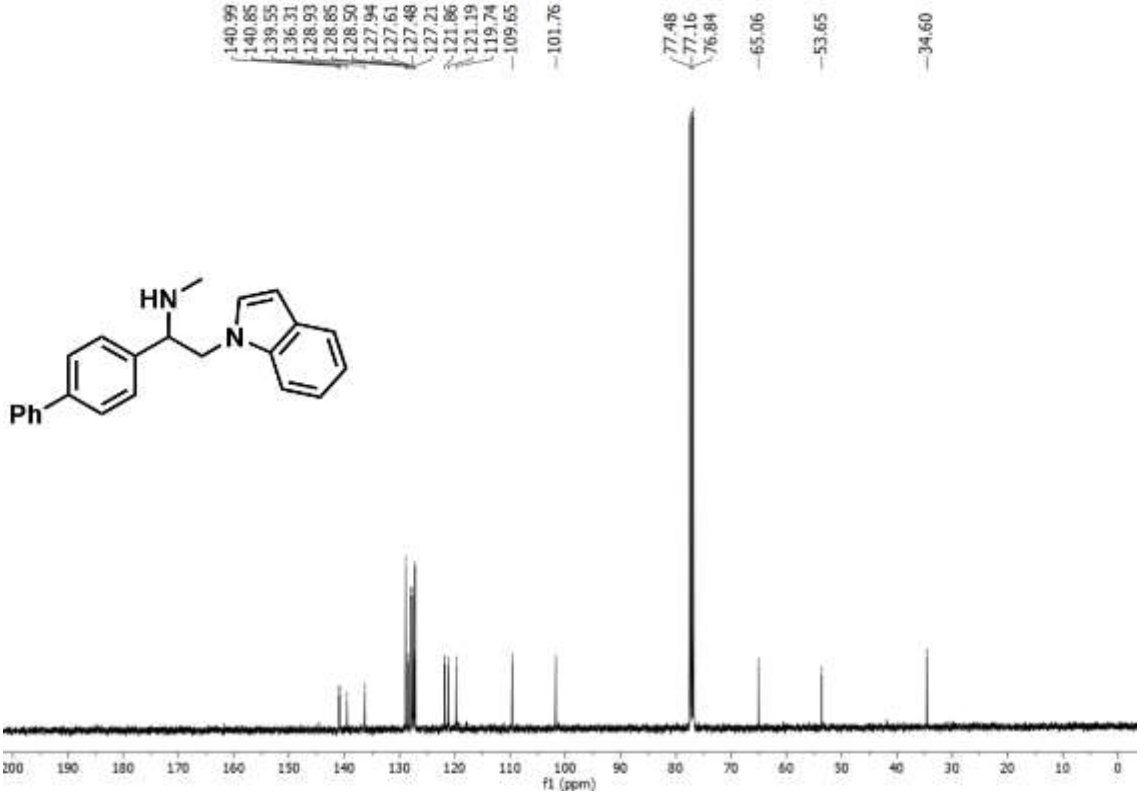

Figure S22. ^{13}C NMR spectrum of **7** (101 MHz, $CDCl_3$)

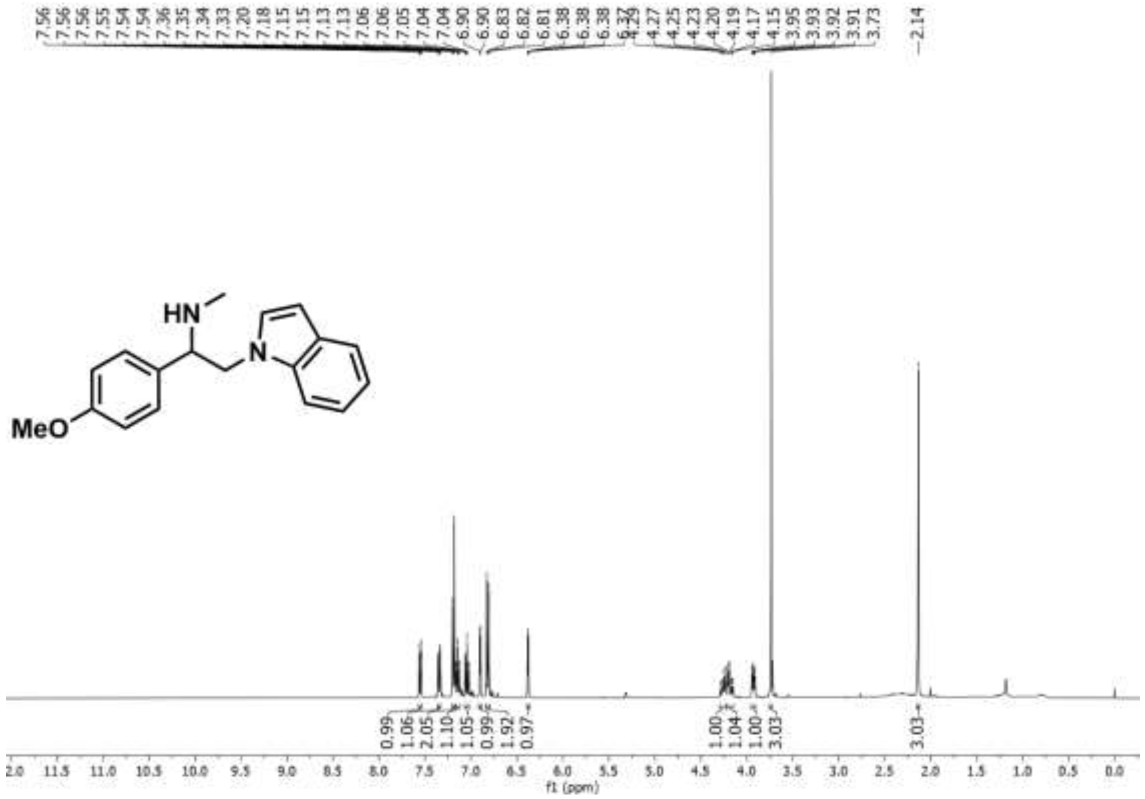

Figure S23. ^{1}H NMR spectrum of **8** (400 MHz, $CDCl_3$)

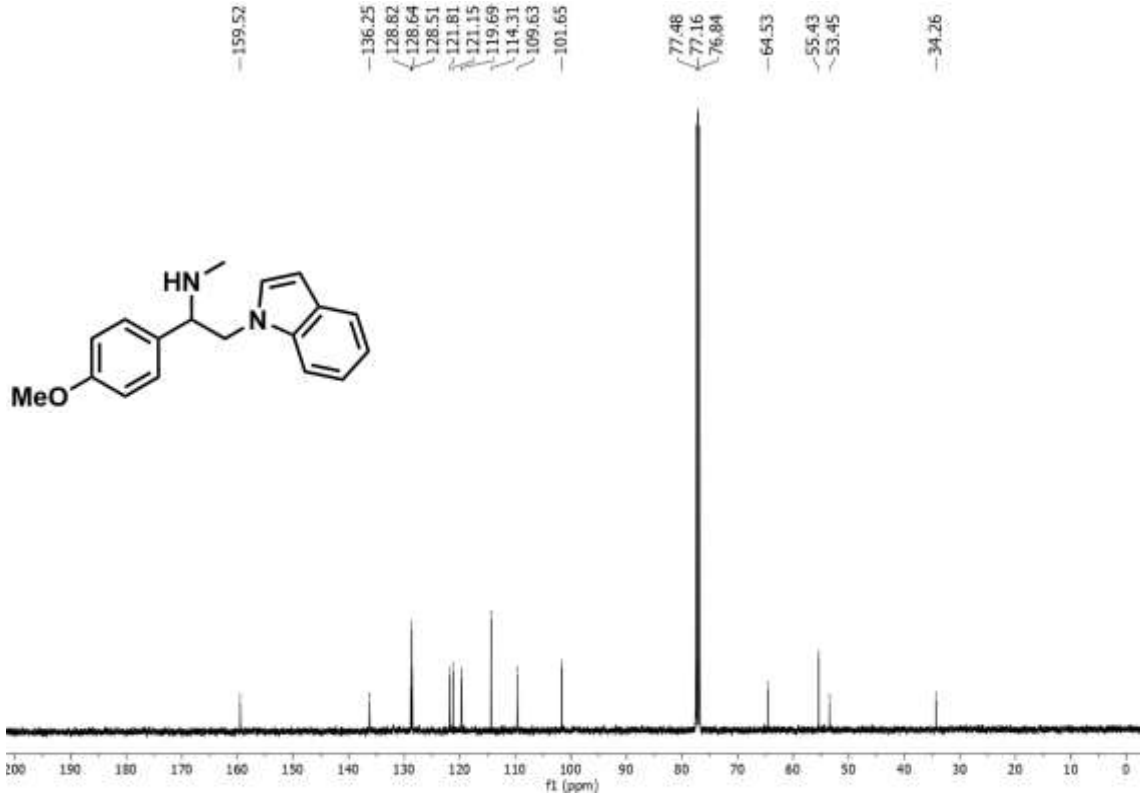

Figure S24. ^{13}C NMR spectrum of **8** (101 MHz, $CDCl_3$)

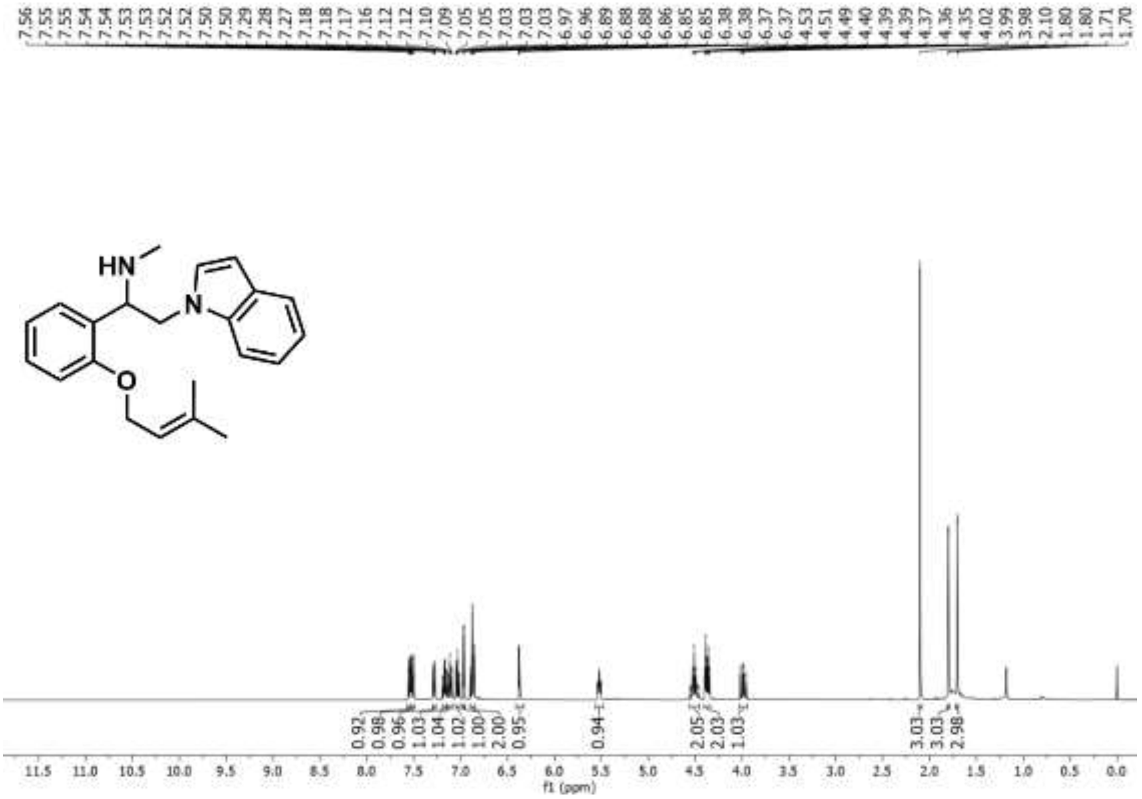

Figure S25. ^{1}H NMR spectrum of **9** (400 MHz, $CDCl_3$)

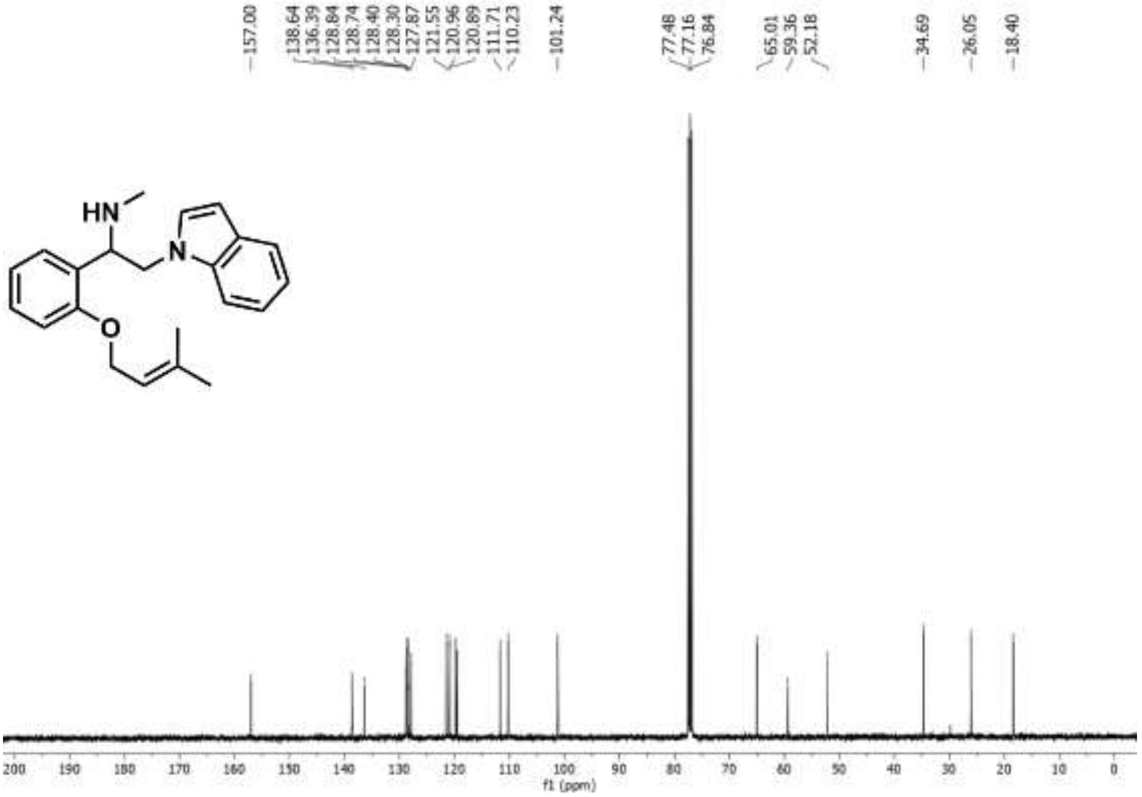

Figure S26. ^{13}C NMR spectrum of **9** (101 MHz, $CDCl_3$)

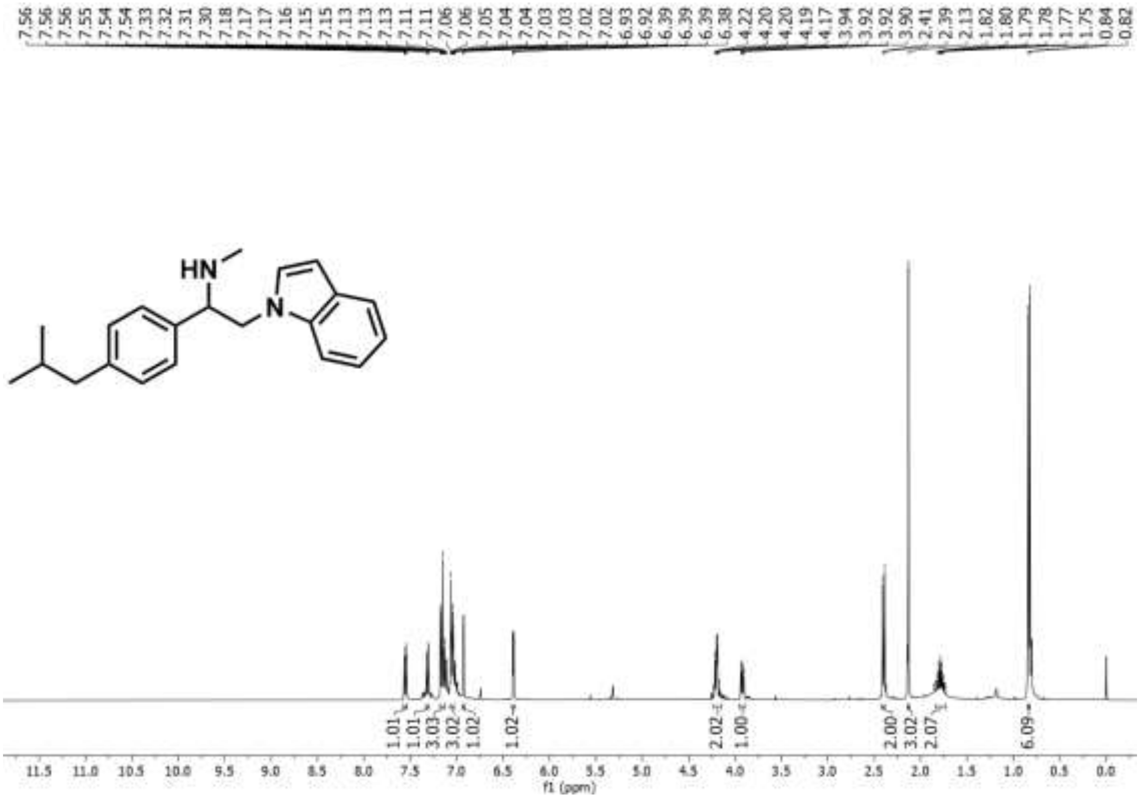

Figure S27. ^{1}H NMR spectrum of **10** (400 MHz, $CDCl_3$)

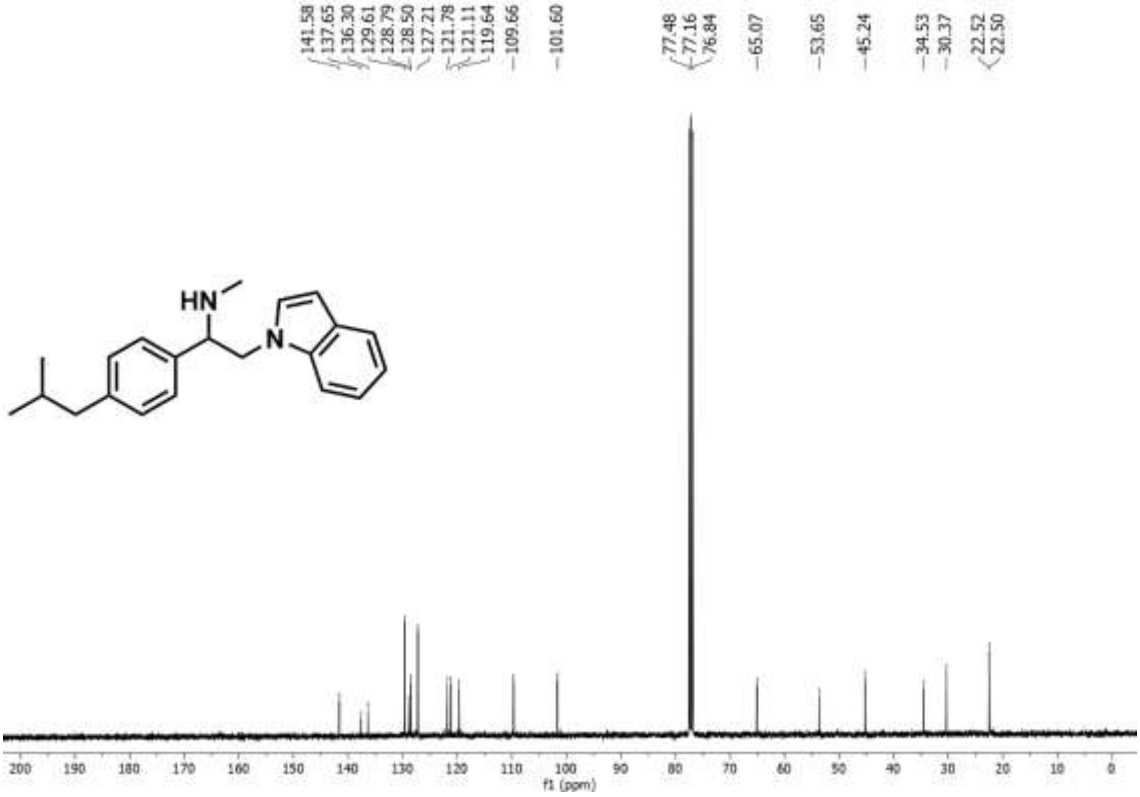

Figure S28. ^{13}C NMR spectrum of **10** (101 MHz, $CDCl_3$)

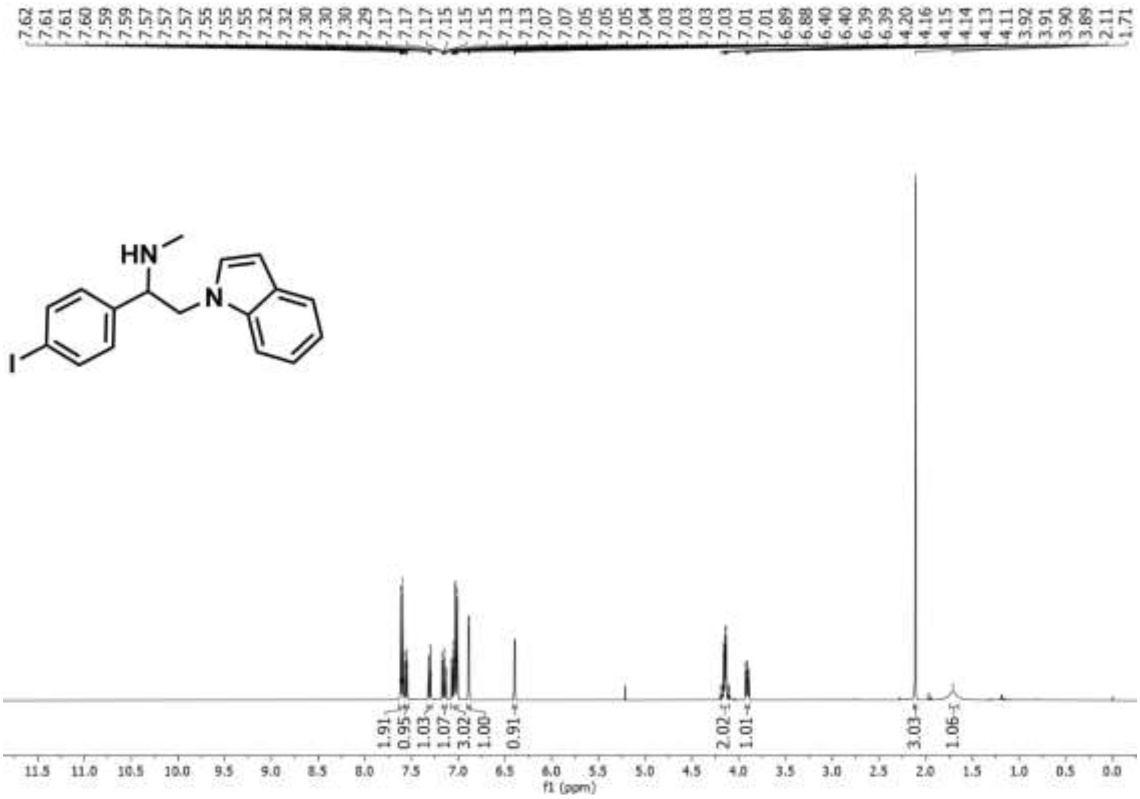

Figure S29. ^{1}H NMR spectrum of **11** (400 MHz, $CDCl_3$)

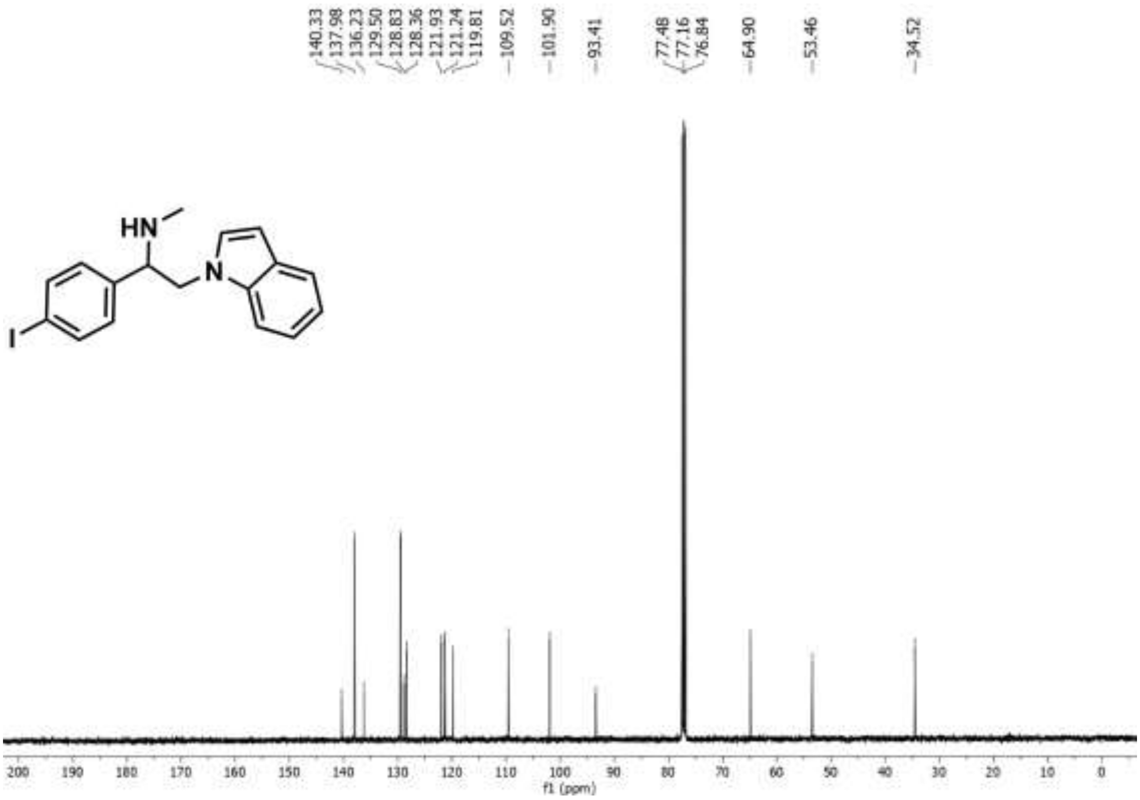

Figure S30. ^{13}C NMR spectrum of **11** (101 MHz, $CDCl_3$)

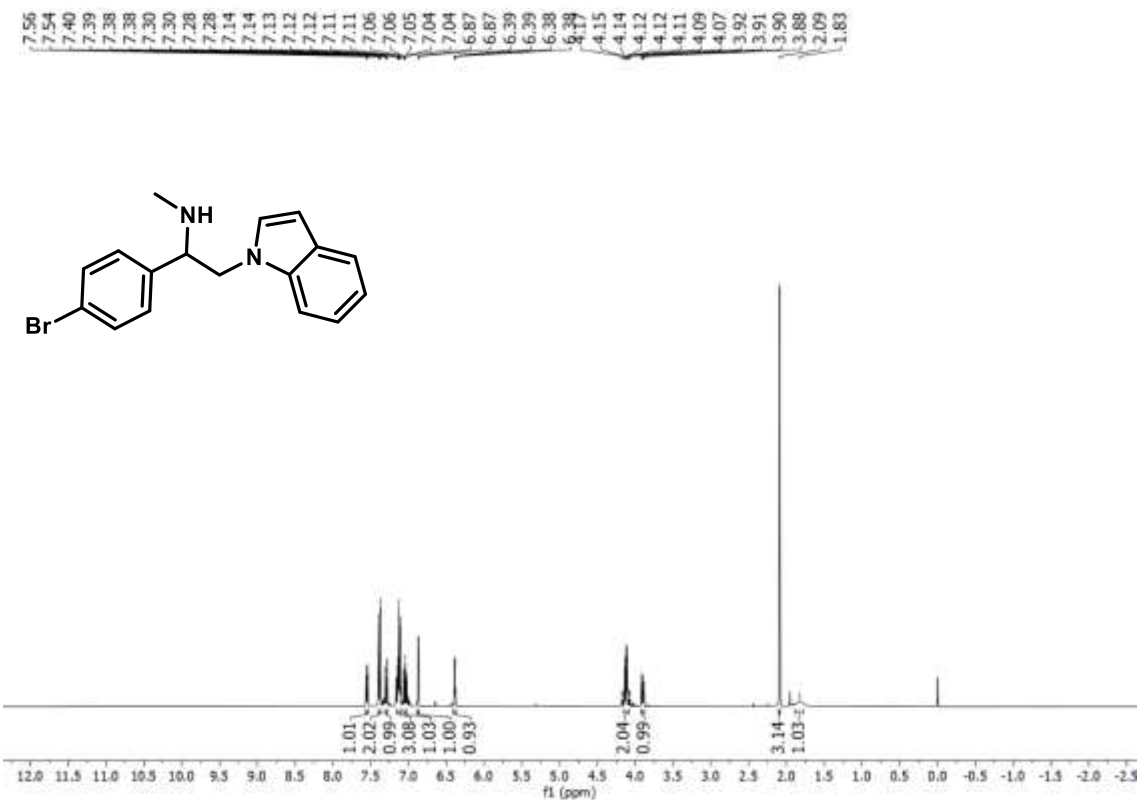

Figure S31. ^{1}H NMR spectrum of **12** (400 MHz, $CDCl_3$)

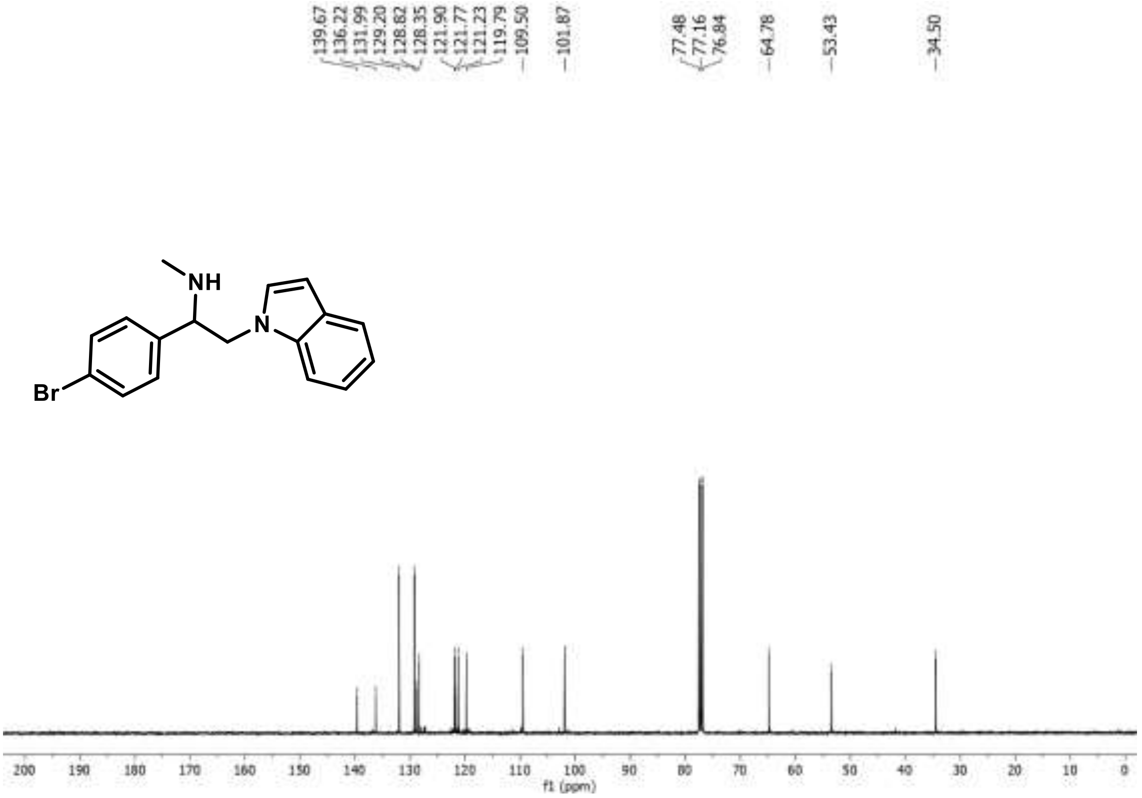

Figure S32. ^{13}C NMR spectrum of **12** (101 MHz, $CDCl_3$)

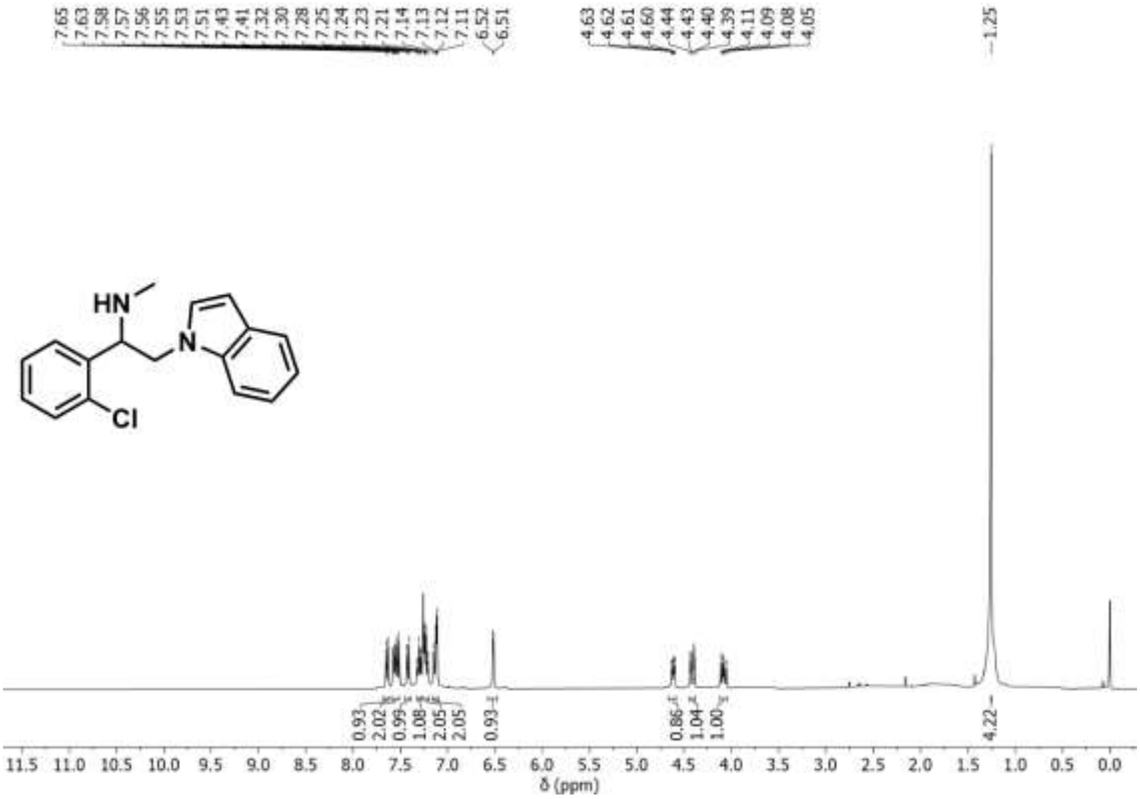

Figure S33. ^{1}H NMR spectrum of **13** (400 MHz, $CDCl_3$)

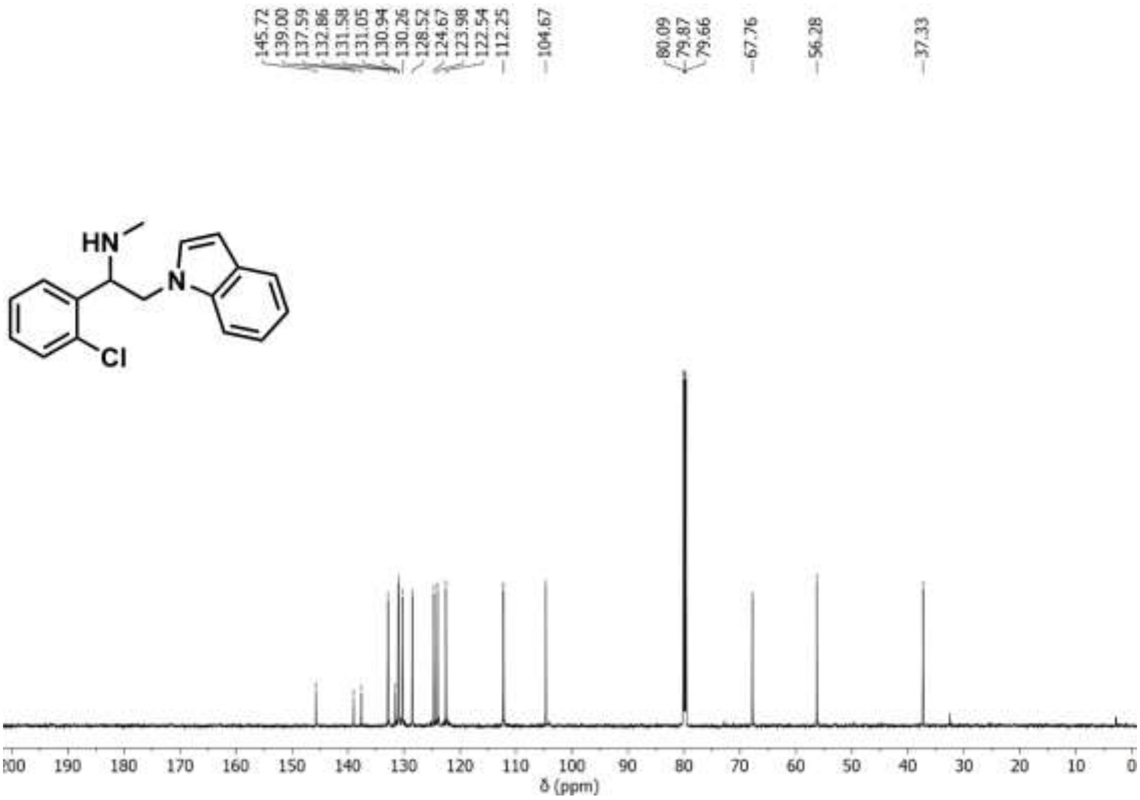

Figure S34. ^{13}C NMR spectrum of **13** (101 MHz, $CDCl_3$)

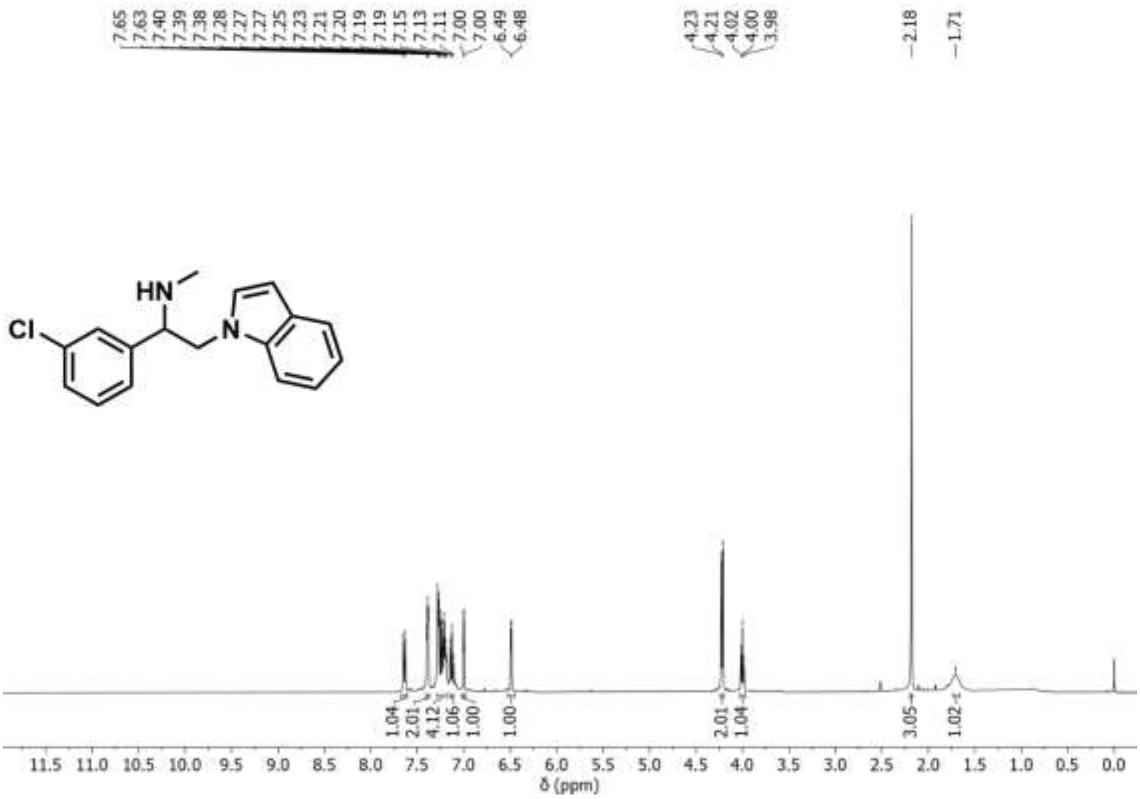

Figure S35. ^{1}H NMR spectrum of **14** (400 MHz, $CDCl_3$)

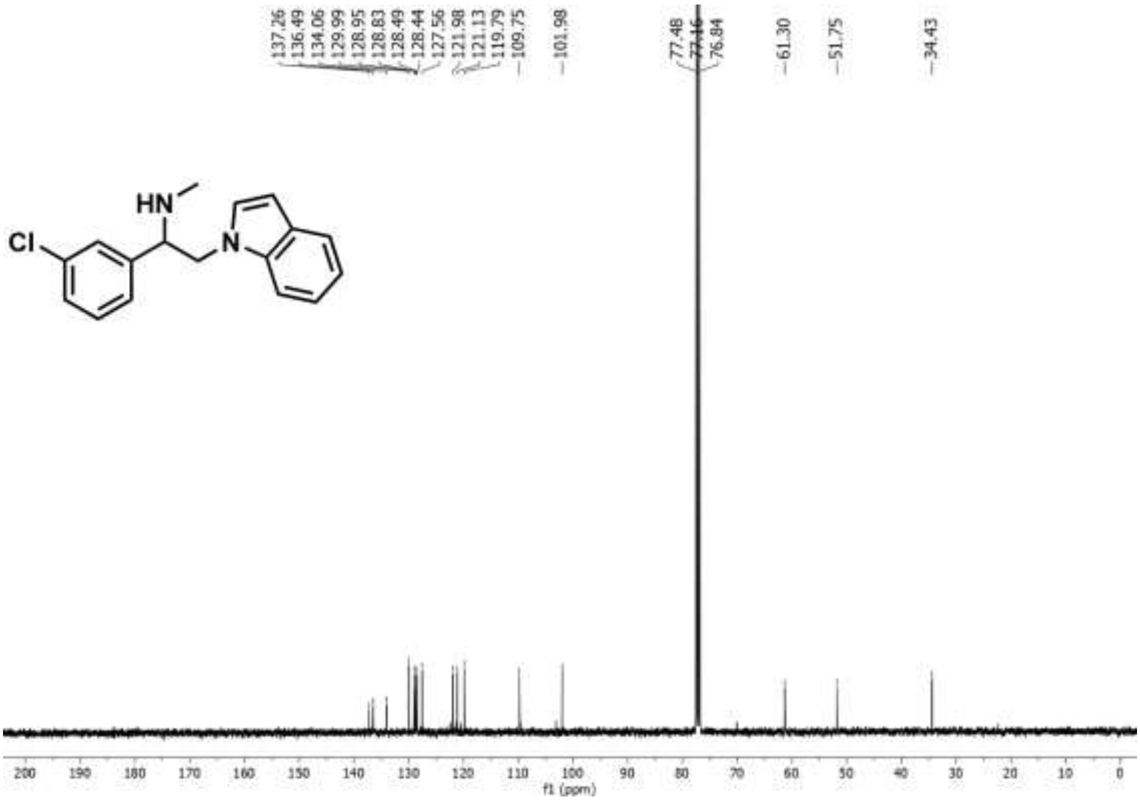

Figure S36. ^{13}C NMR spectrum of **14** (101 MHz, $CDCl_3$)

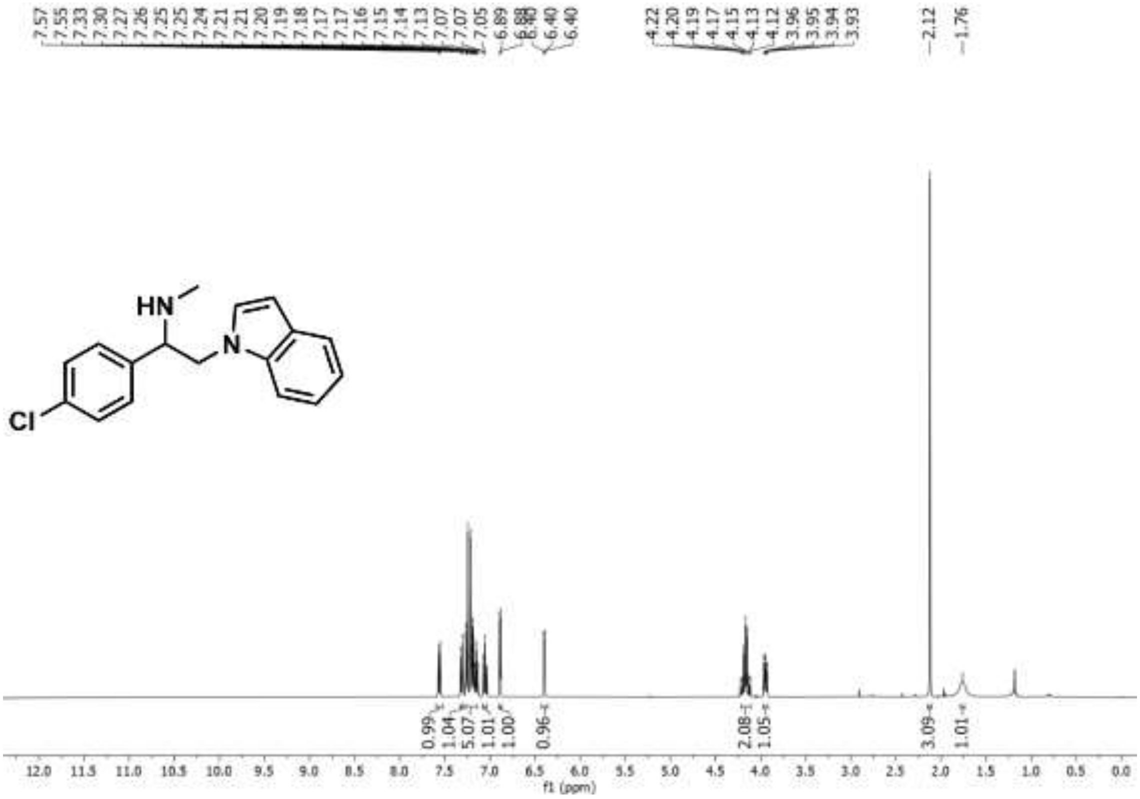

Figure S37. ^{1}H NMR spectrum of **15** (400 MHz, $CDCl_3$)

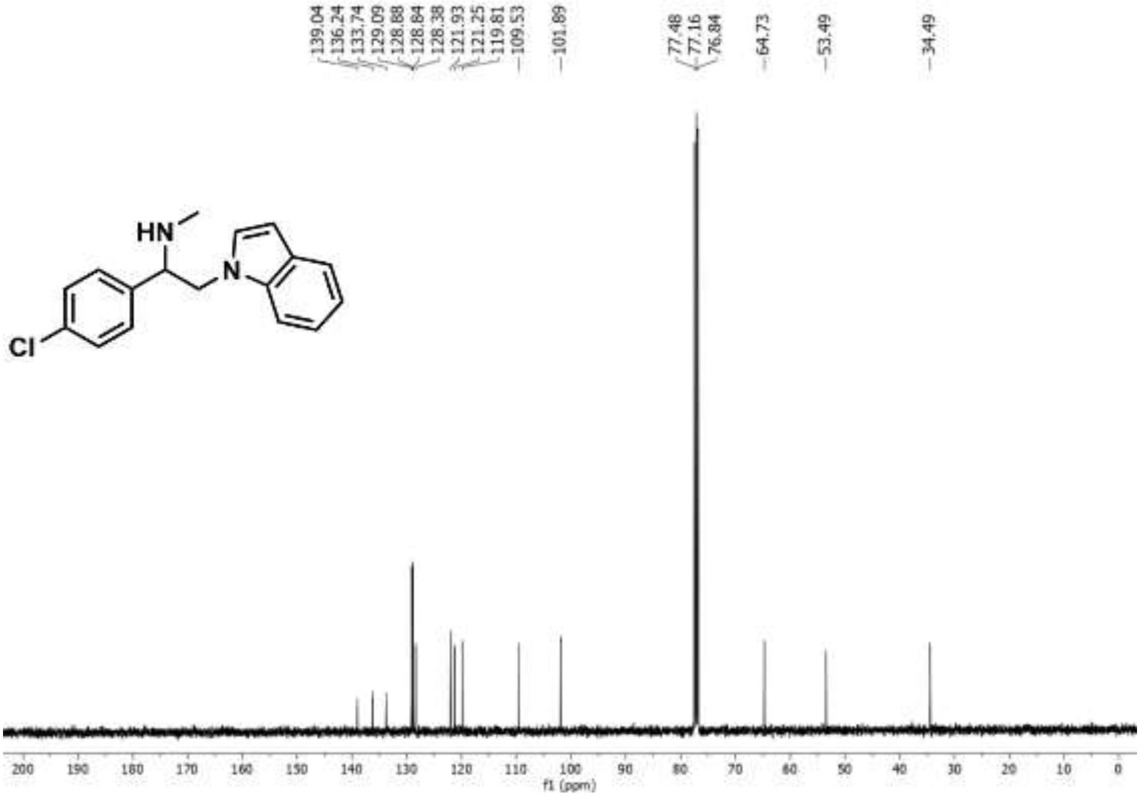

Figure S38. ^{13}C NMR spectrum of **15** (101 MHz, $CDCl_3$)

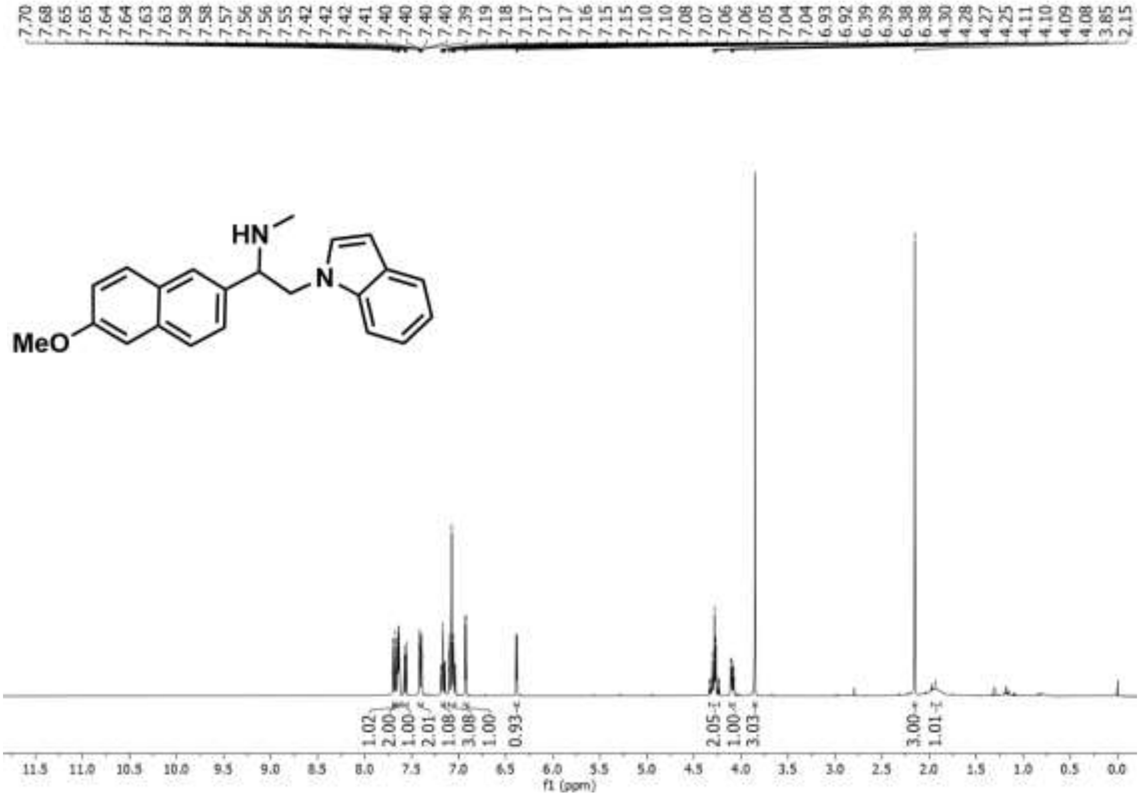

Figure S39. ^{1}H NMR spectrum of **16** (400 MHz, $CDCl_3$)

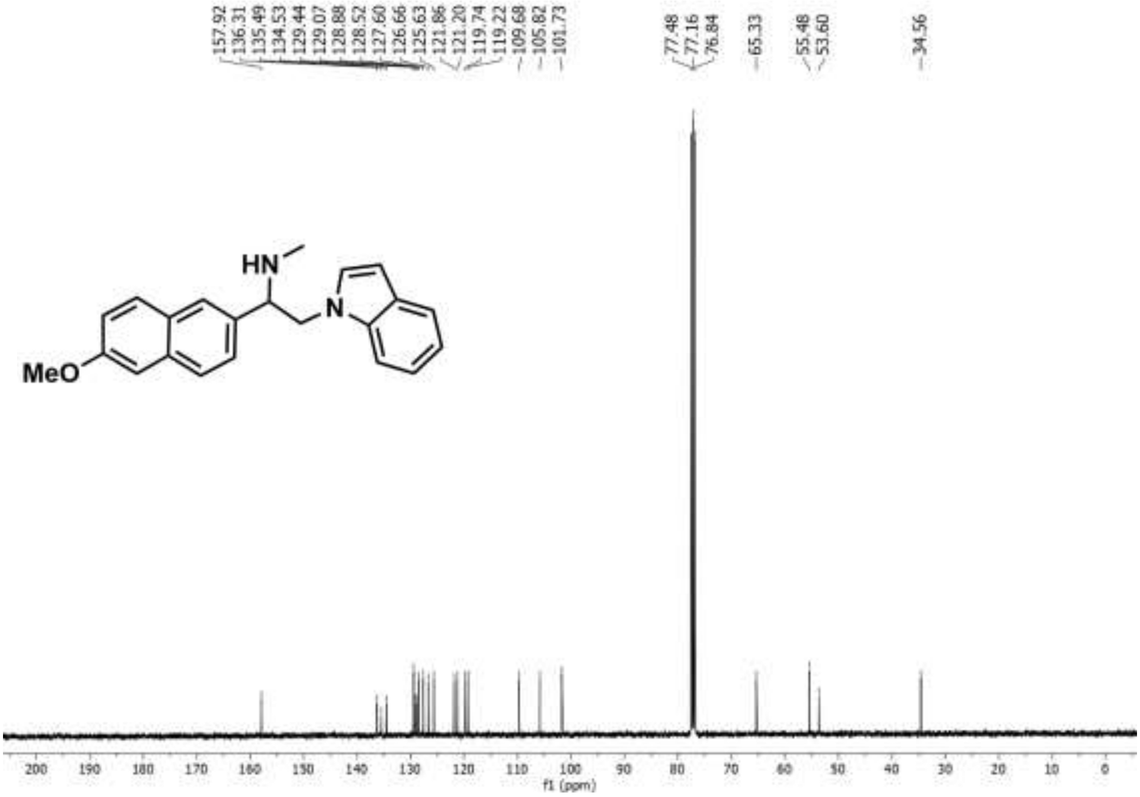

Figure S40. ^{13}C NMR spectrum of **16** (101 MHz, $CDCl_3$)

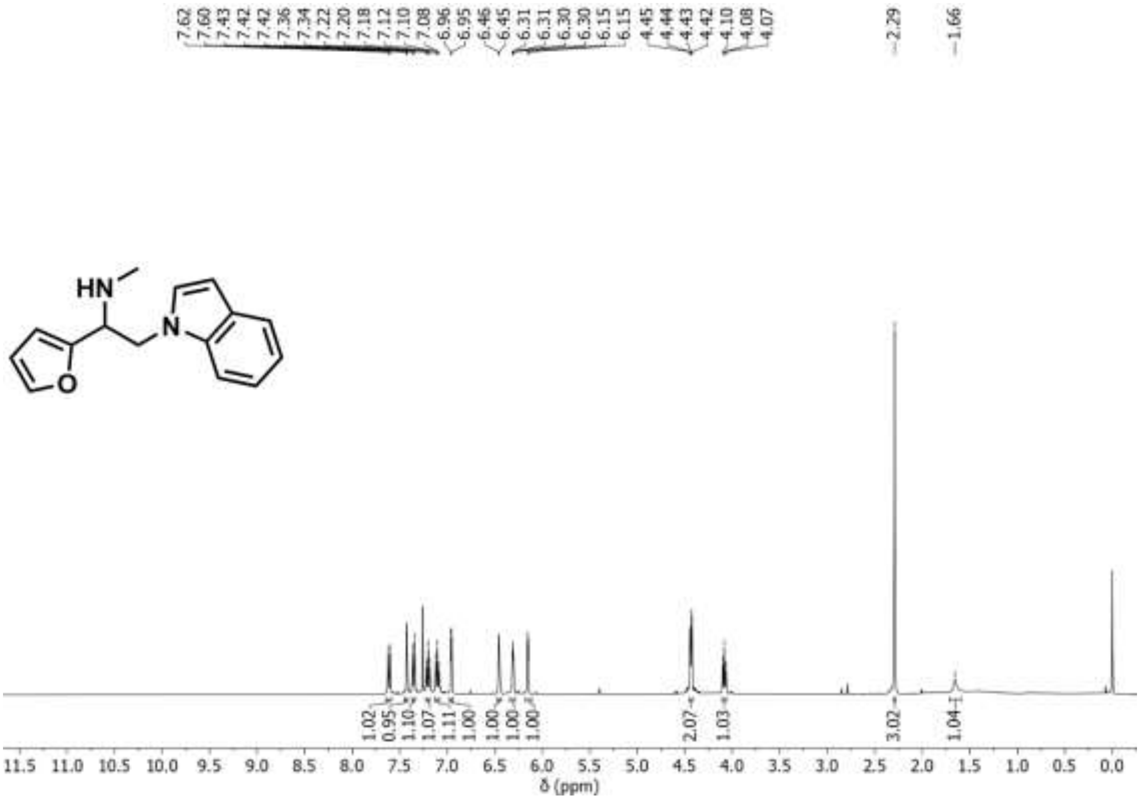

Figure S41. ^{1}H NMR spectrum of **17** (400 MHz, $CDCl_3$)

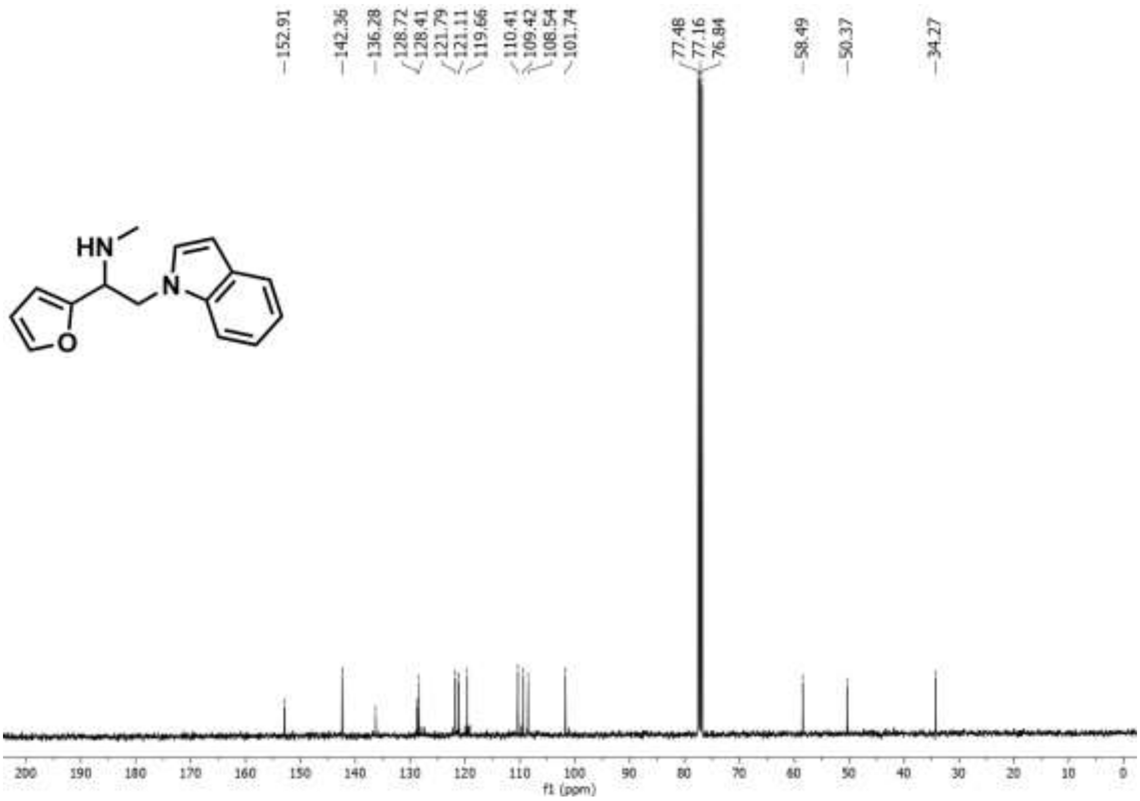

Figure S42. ^{13}C NMR spectrum of **17** (101 MHz, $CDCl_3$)

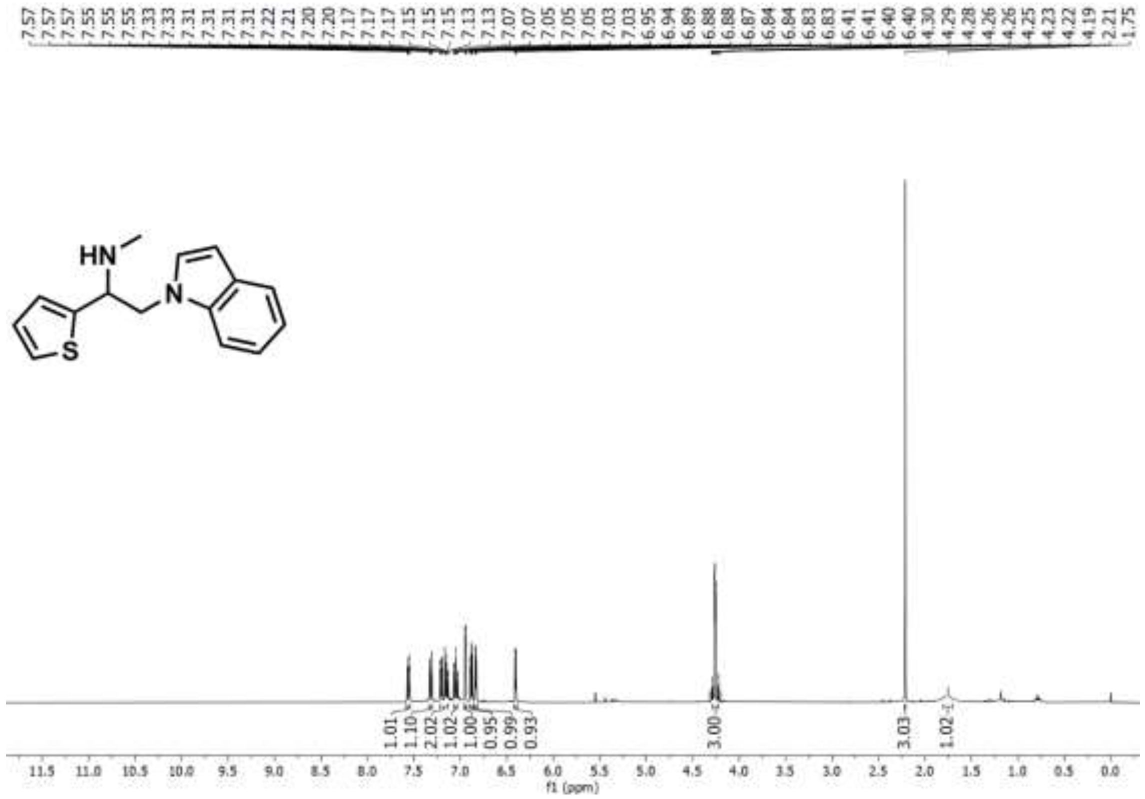

Figure S43. ^{1}H NMR spectrum of **18** (400 MHz, $CDCl_3$)

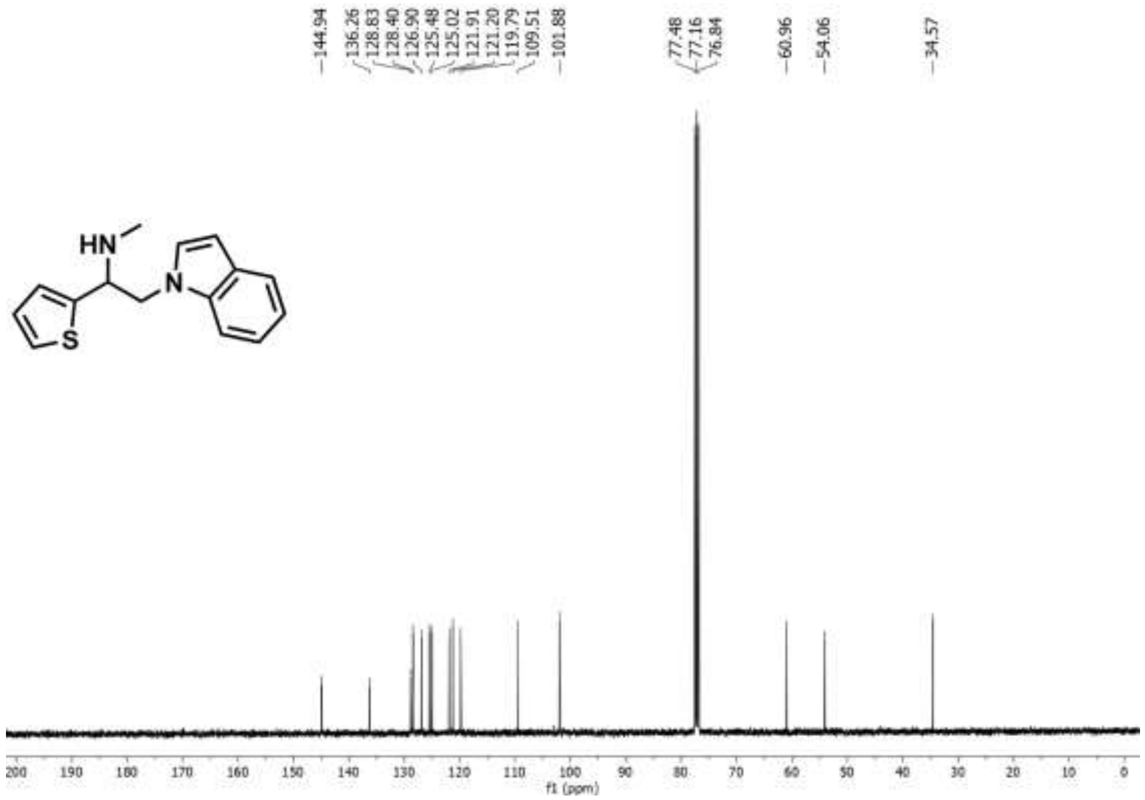

Figure S44. ^{13}C NMR spectrum of **18** (101 MHz, $CDCl_3$)

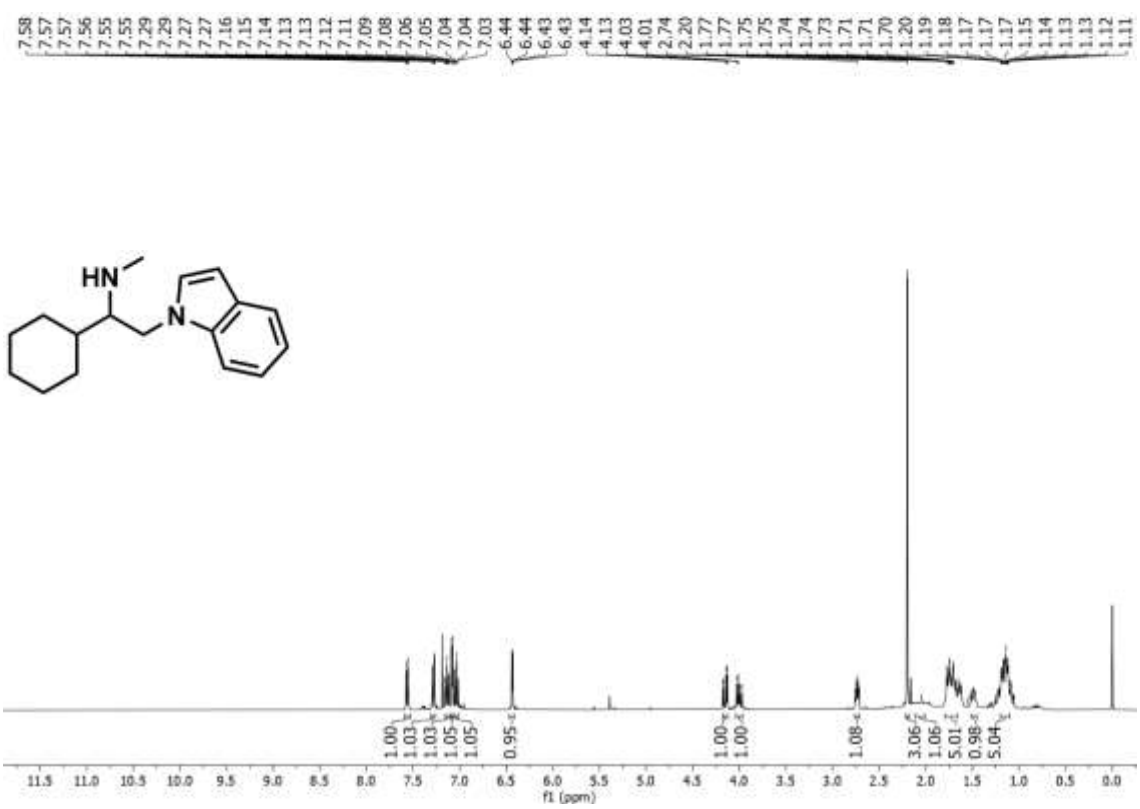

Figure S45. ^{1}H NMR spectrum of **19** (400 MHz, $CDCl_3$)

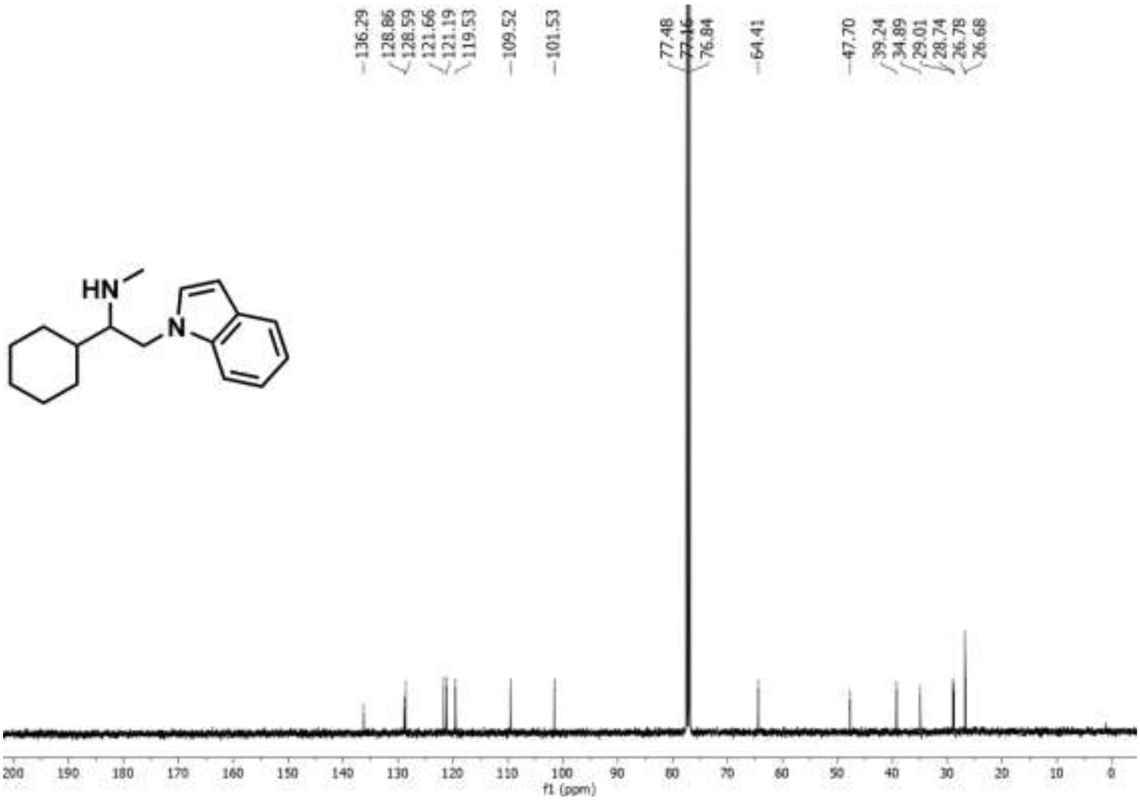

Figure S46. ^{13}C NMR spectrum of **19** (101 MHz, $CDCl_3$)

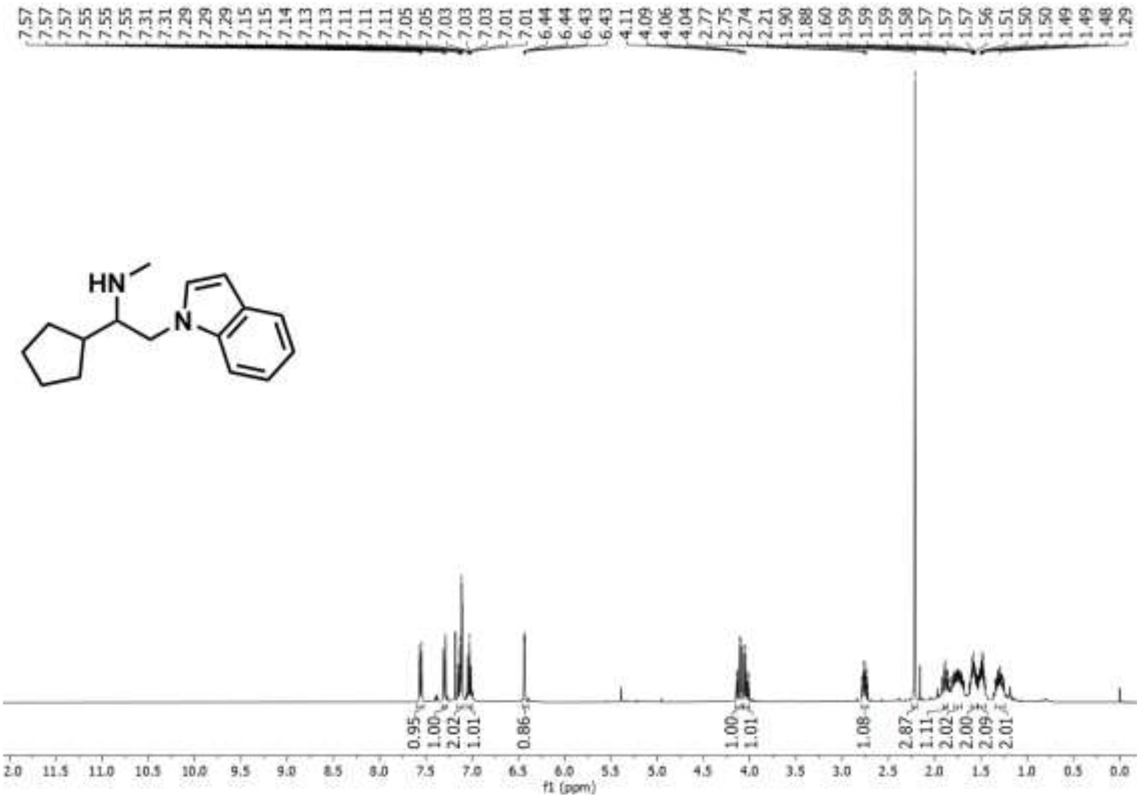

Figure S47. ^{1}H NMR spectrum of **20** (400 MHz, $CDCl_3$)

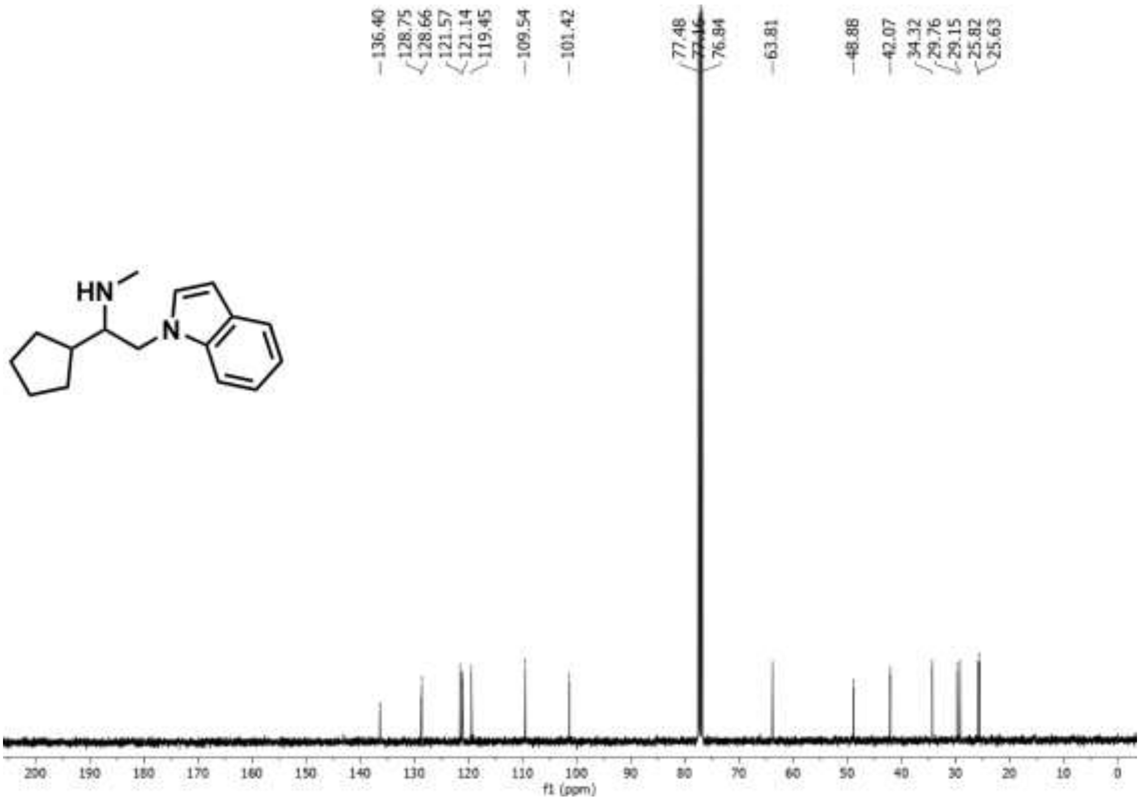

Figure S48. ^{13}C NMR spectrum of **20** (101 MHz, $CDCl_3$)

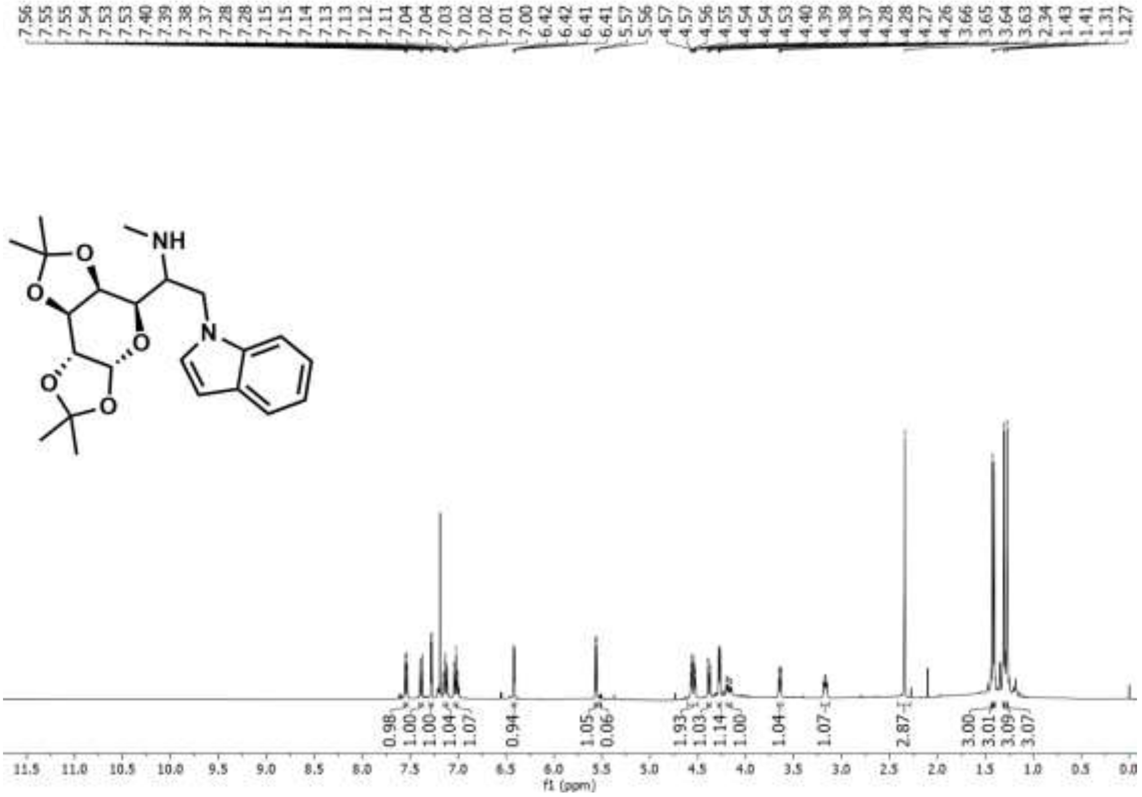

Figure S49. ^{1}H NMR spectrum of **21** (400 MHz, $CDCl_3$)

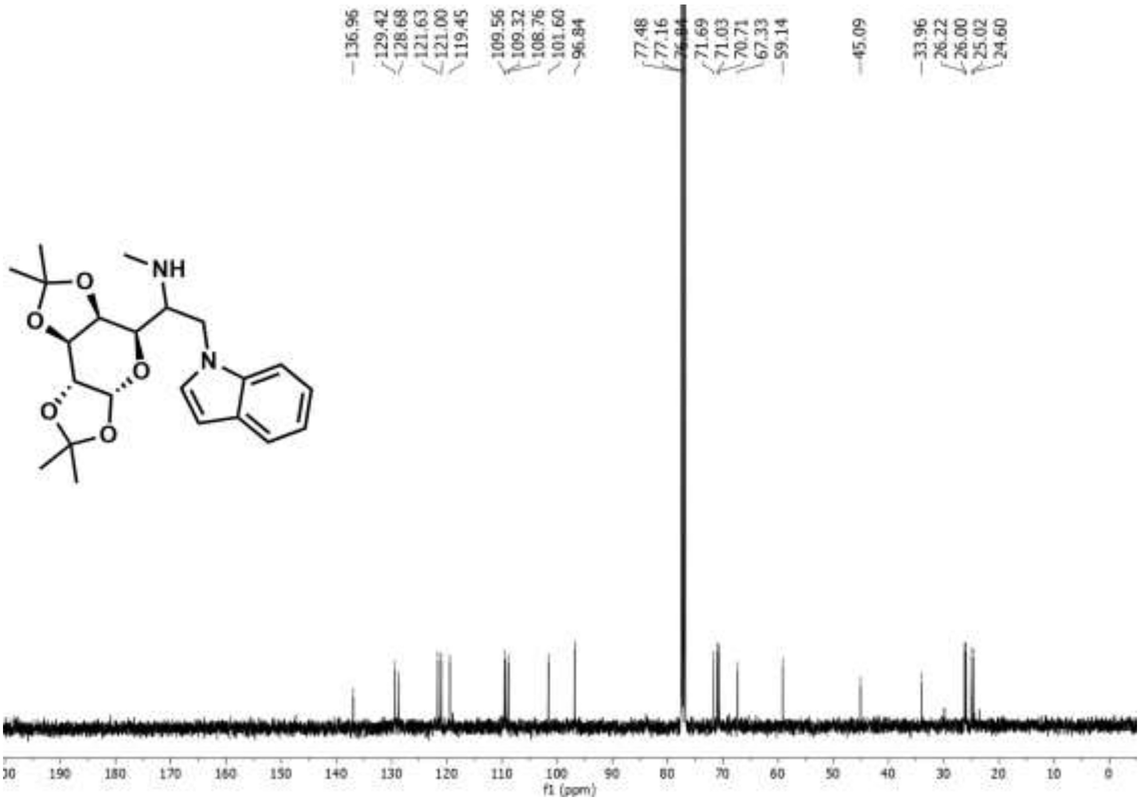

Figure S50. ^{13}C NMR spectrum of **21** (101 MHz, $CDCl_3$)

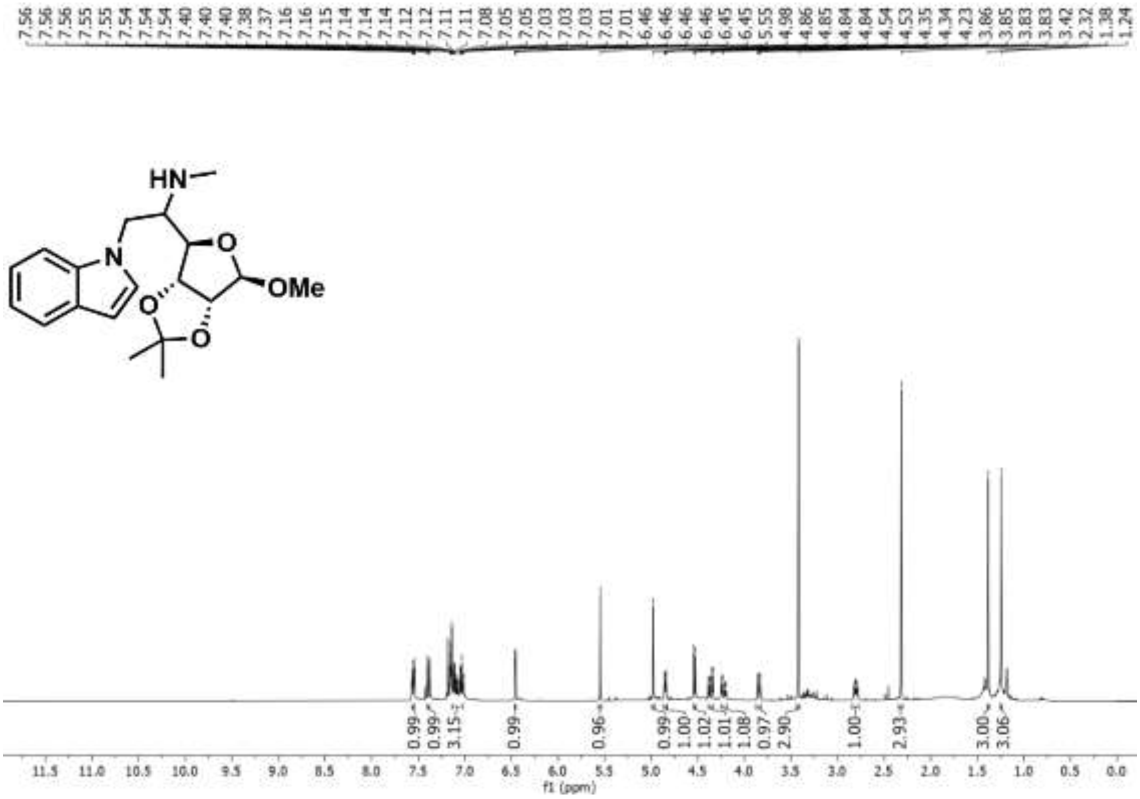

Figure S51. ^{1}H NMR spectrum of **22** (400 MHz, $CDCl_3$)

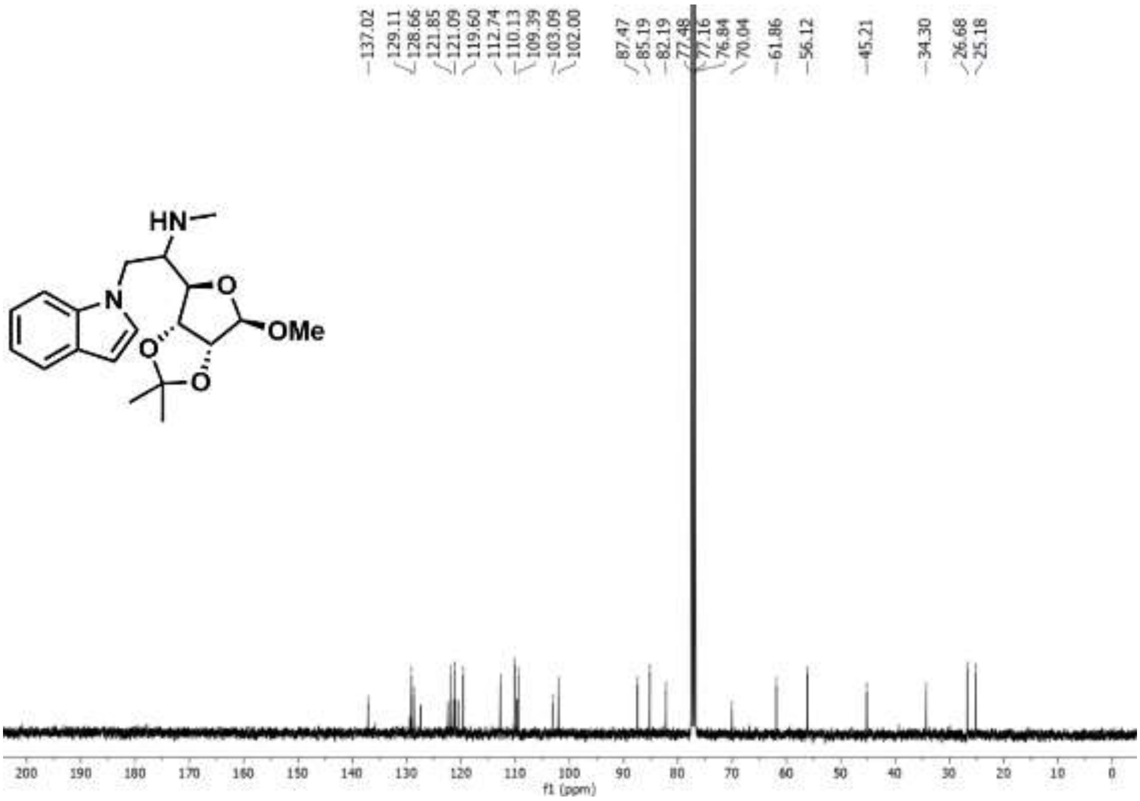

Figure S52. ^{13}C NMR spectrum of **22** (101 MHz, $CDCl_3$)

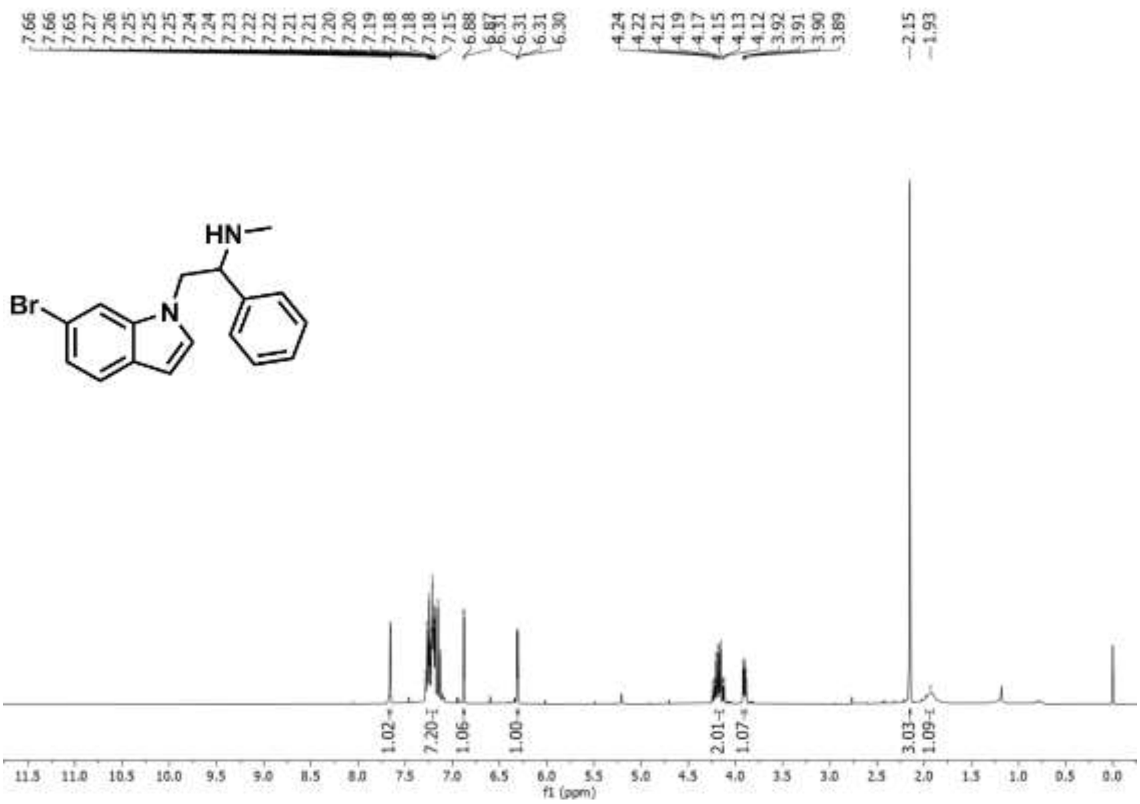

Figure S53. ^{1}H NMR spectrum of **23** (400 MHz, $CDCl_3$)

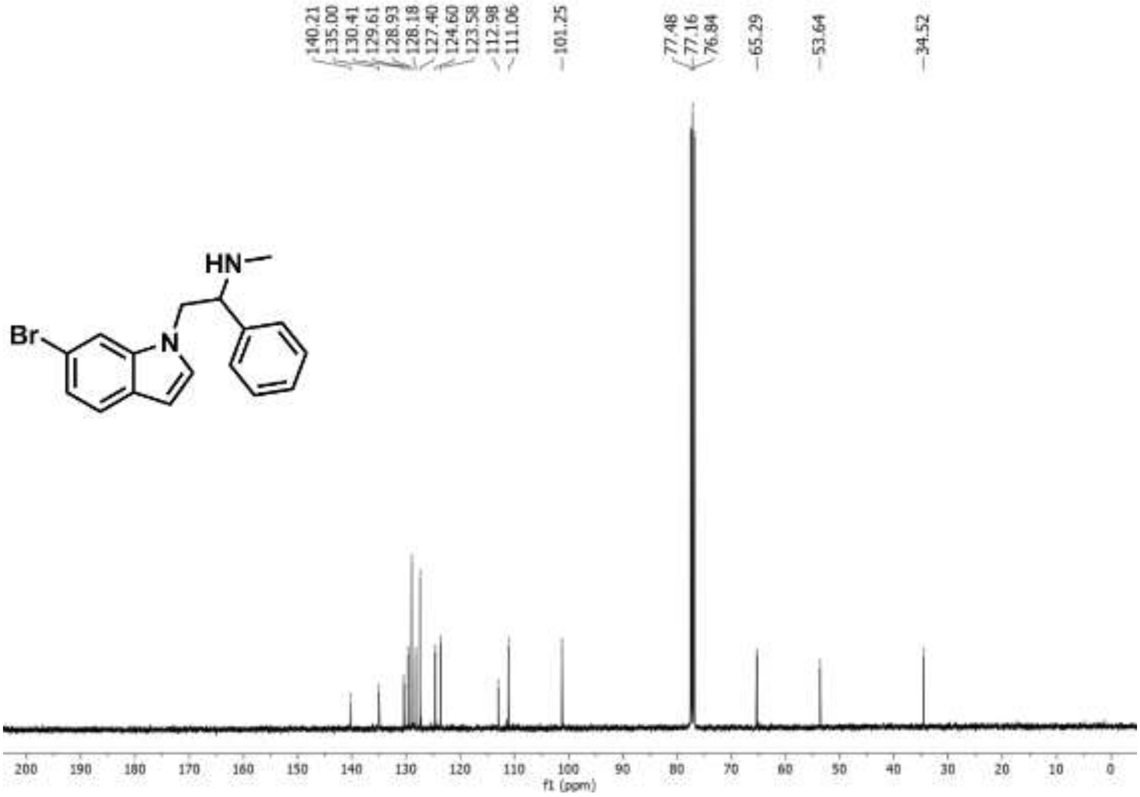

Figure S54. ^{13}C NMR spectrum of **23** (101 MHz, $CDCl_3$)

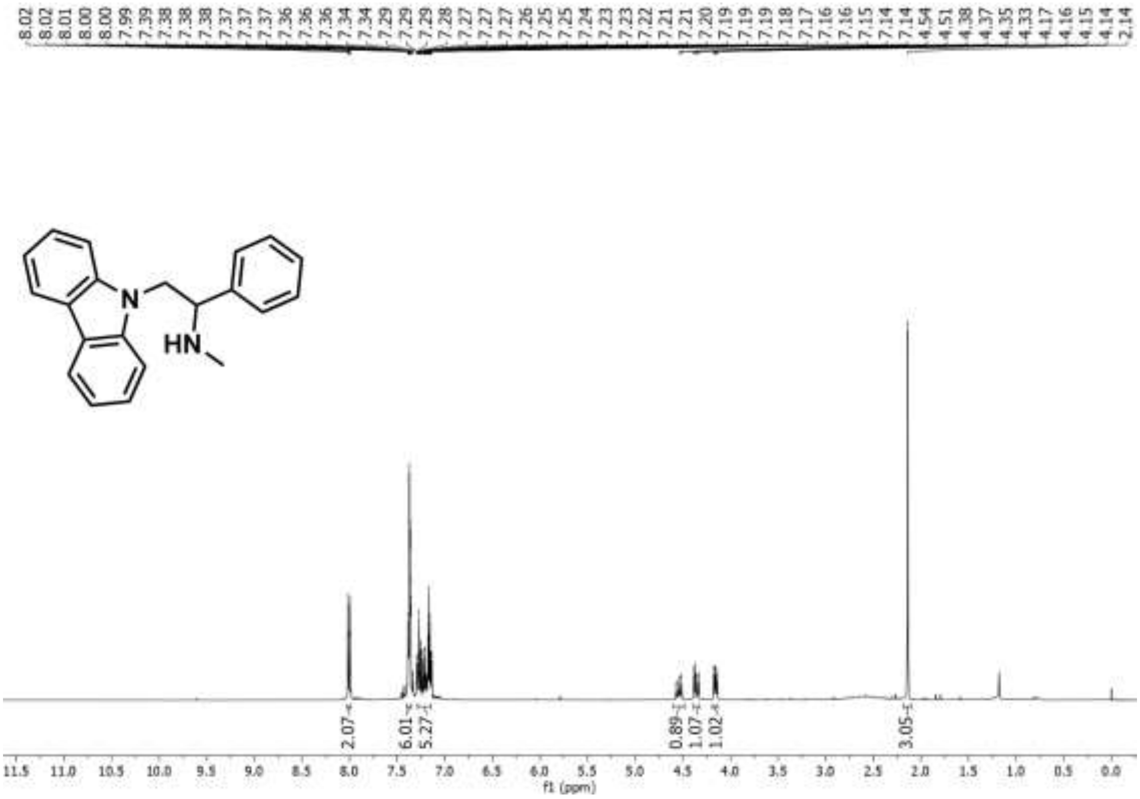

Figure S55. ^{1}H NMR spectrum of **24** (400 MHz, $CDCl_3$)

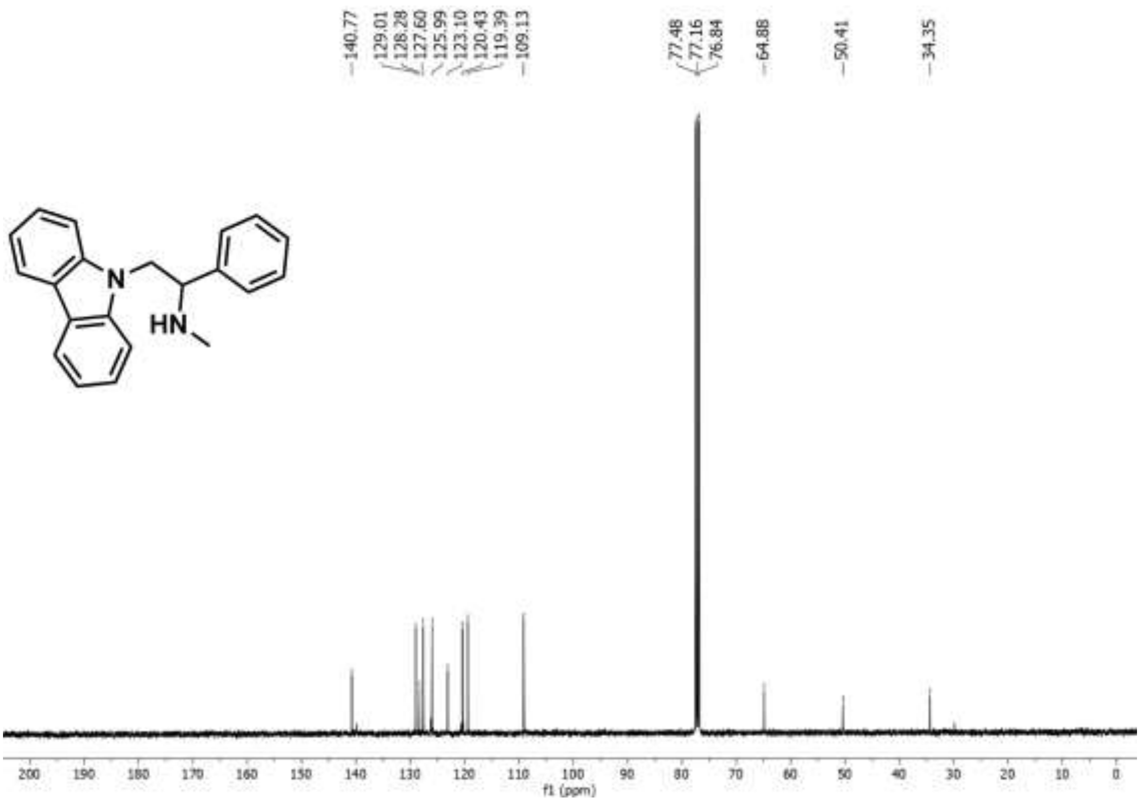

Figure S56. ^{13}C NMR spectrum of **24** (101 MHz, $CDCl_3$)

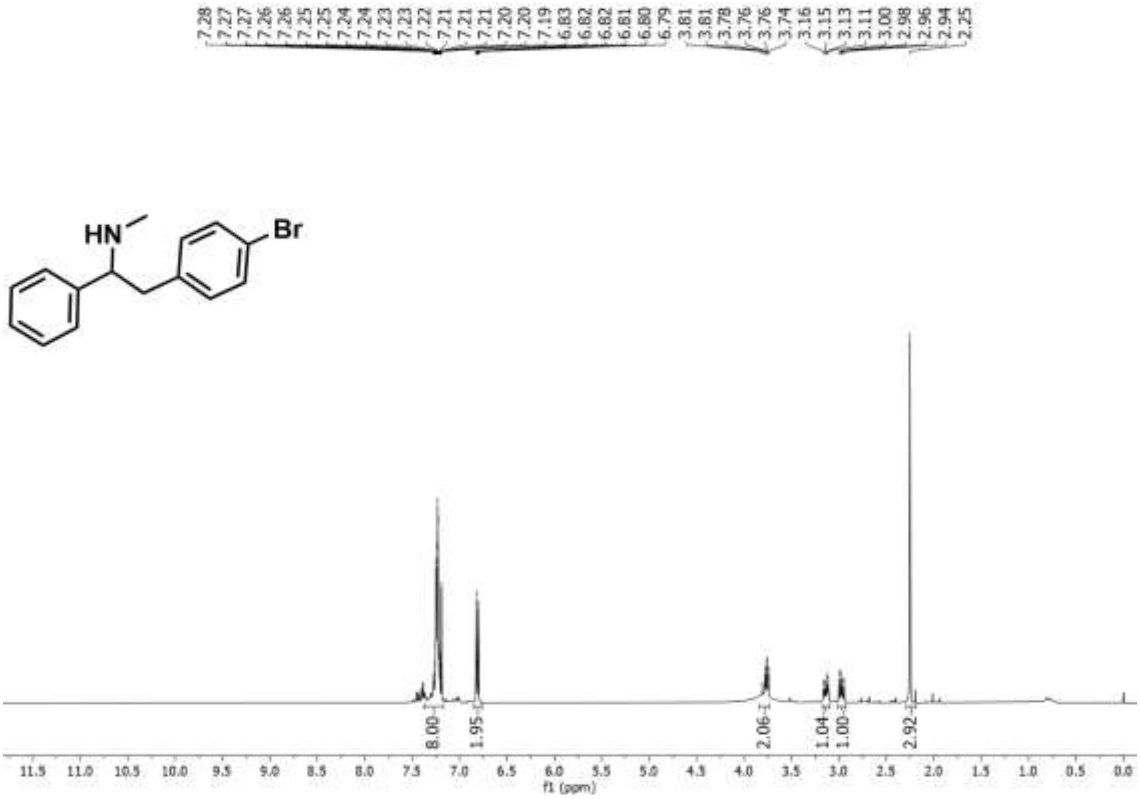

Figure S57. ^{1}H NMR spectrum of **25** (400 MHz, $CDCl_3$)

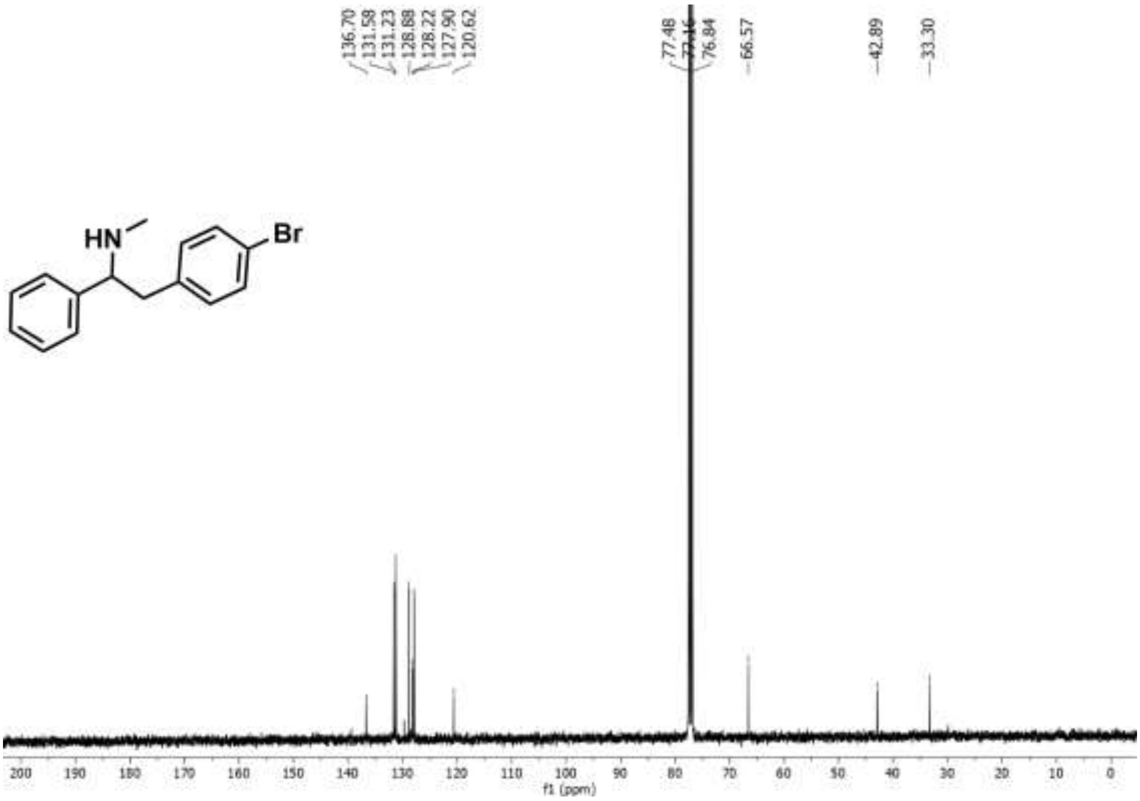

Figure S58. ^{13}C NMR spectrum of **25** (101 MHz, $CDCl_3$)

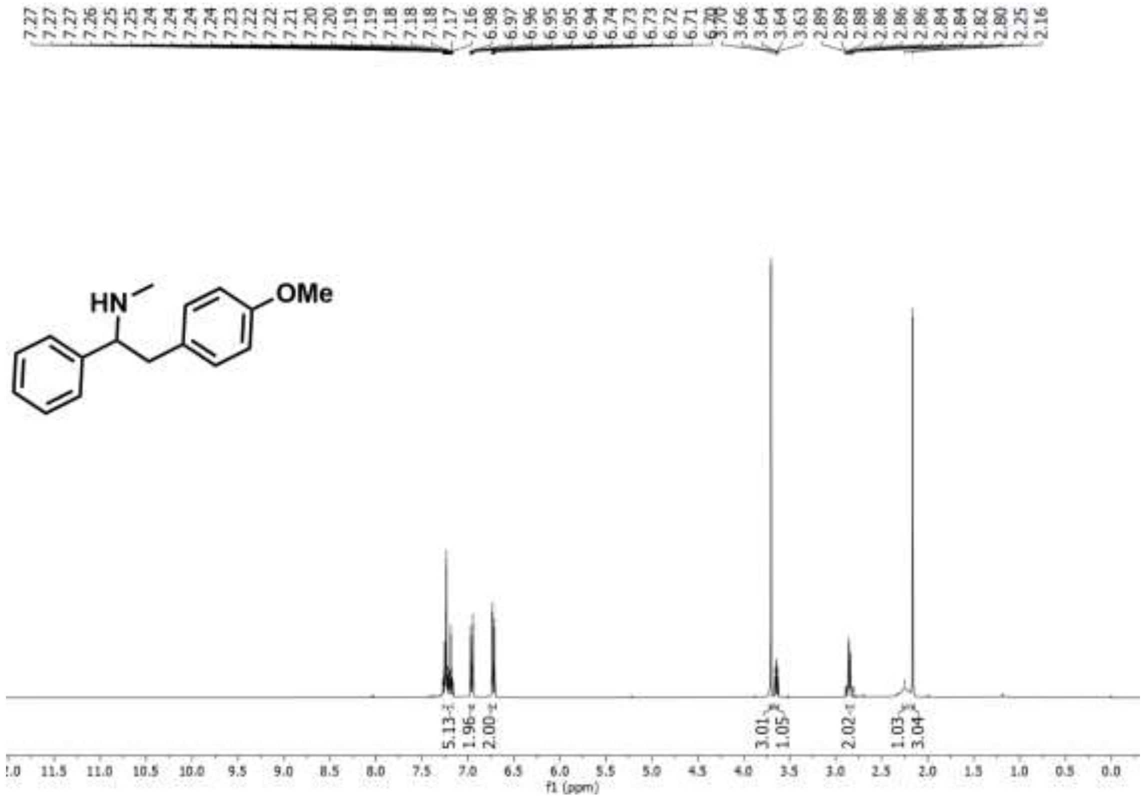

Figure S59. ^{1}H NMR spectrum of **26** (400 MHz, $CDCl_3$)

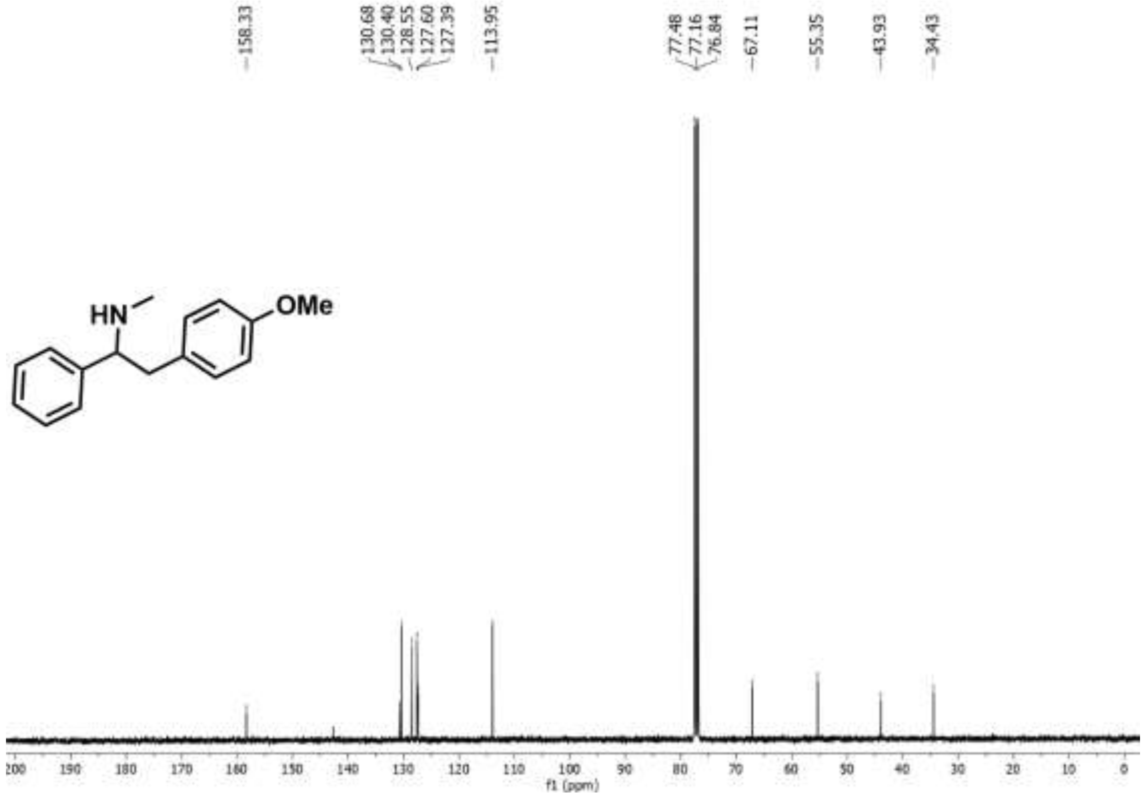

Figure S60. ^{13}C NMR spectrum of **26** (101 MHz, $CDCl_3$)

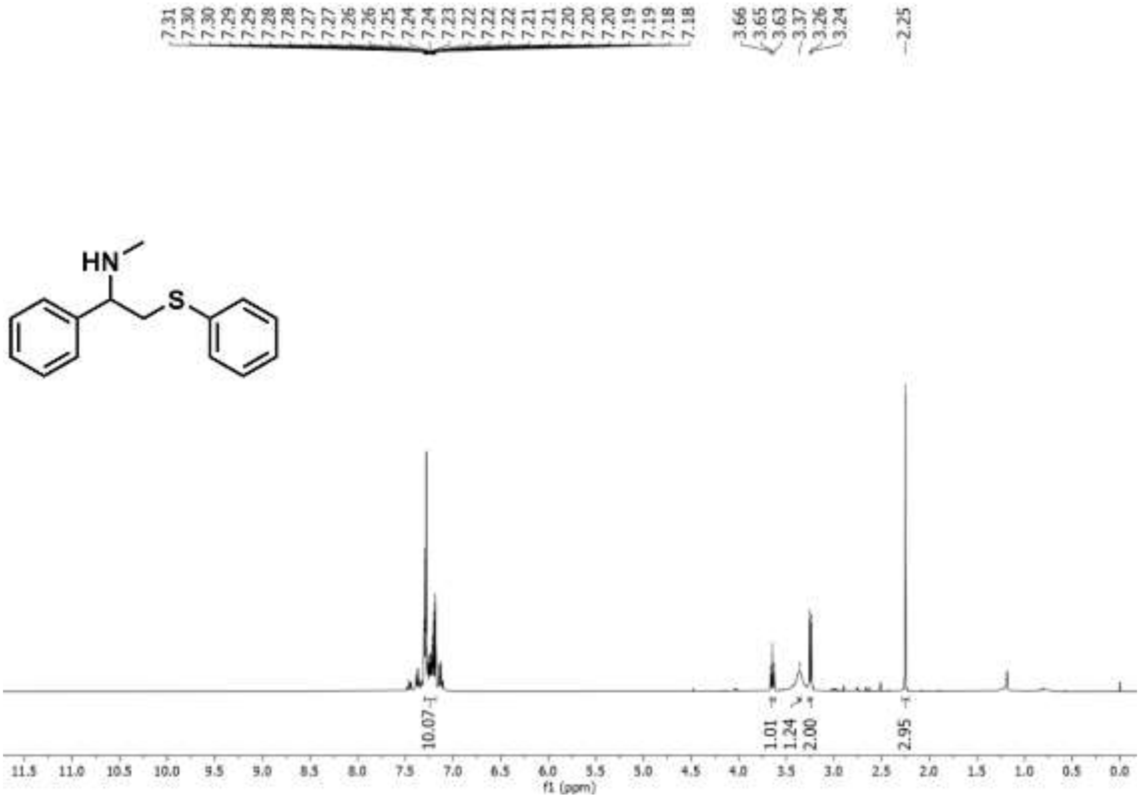

Figure S61. ^{1}H NMR spectrum of **27** (400 MHz, $CDCl_3$)

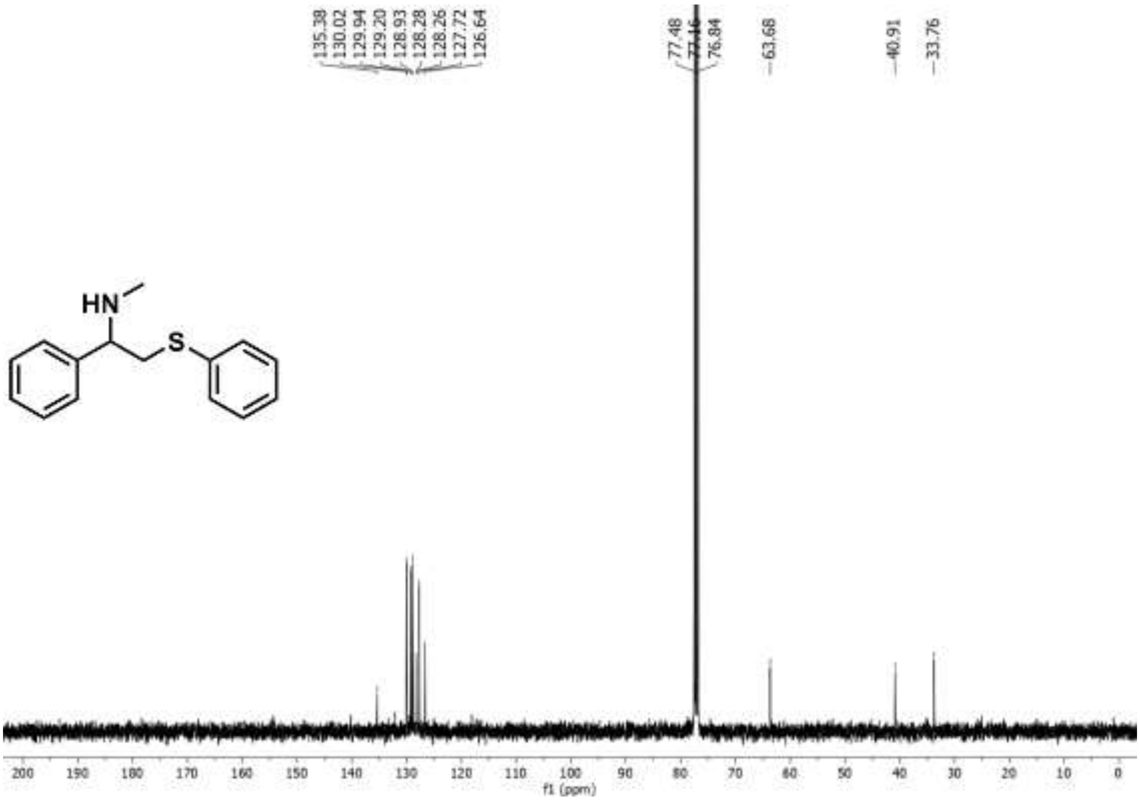

Figure S62. ^{13}C NMR spectrum of **27** (101 MHz, $CDCl_3$)

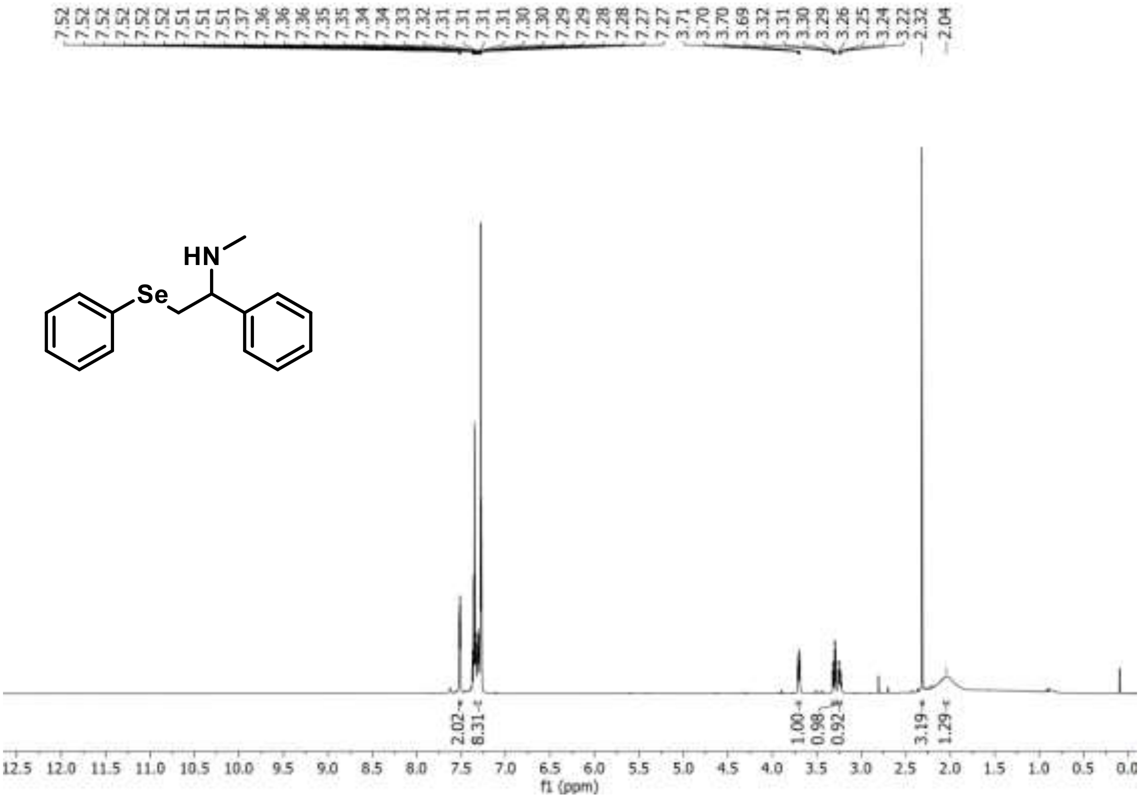

Figure S63. ^{1}H NMR spectrum of **28** (600 MHz, $CDCl_3$)

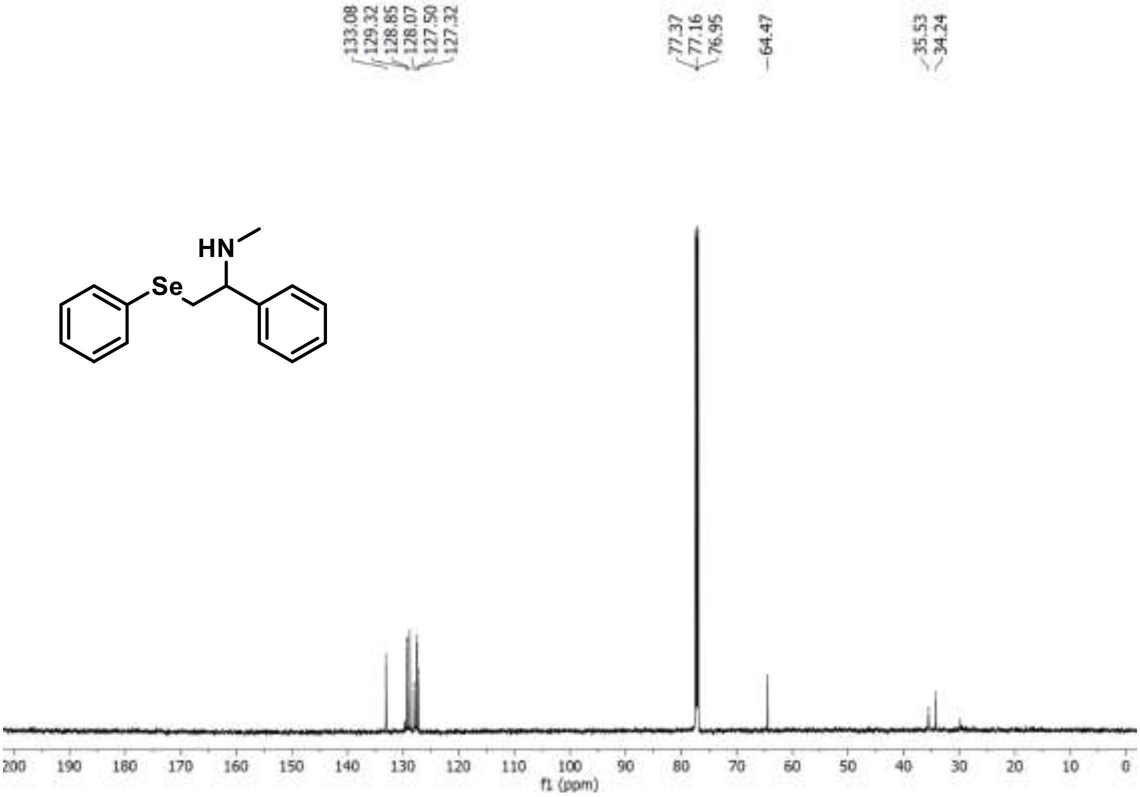

Figure S64. ^{13}C NMR spectrum of **28** (151 MHz, $CDCl_3$)

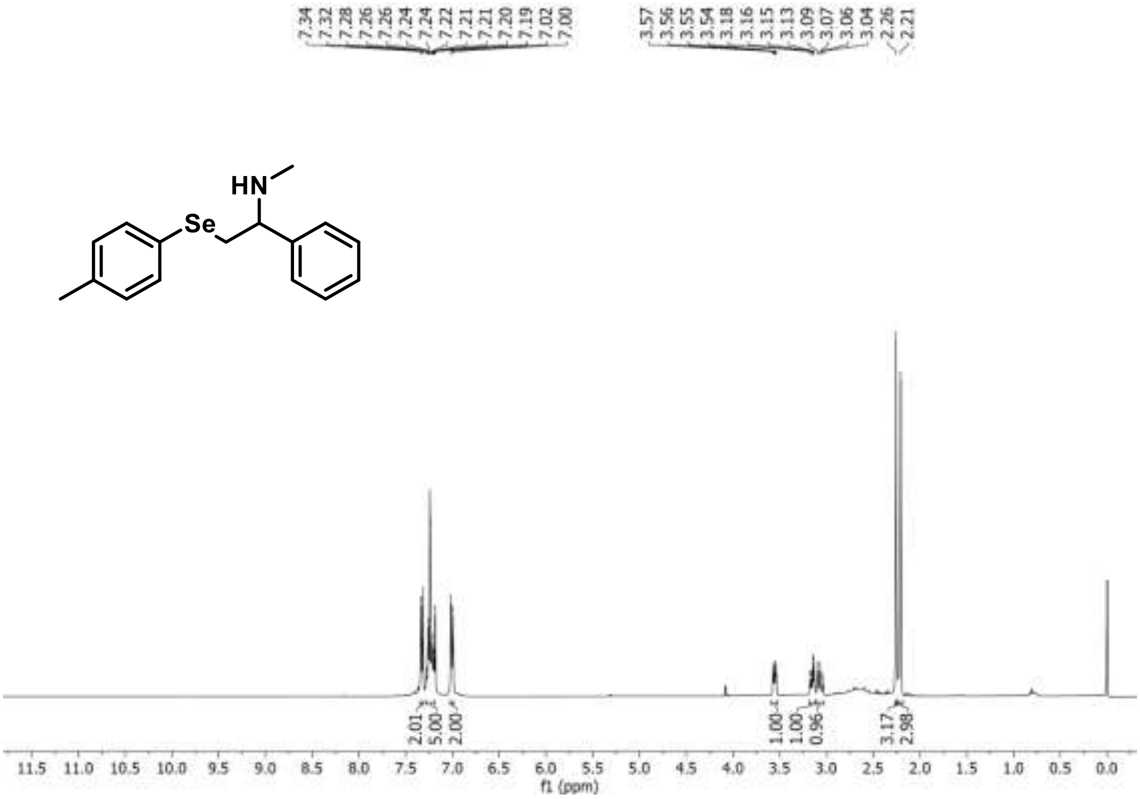

Figure S65. ^{1}H NMR spectrum of **29** (400 MHz, $CDCl_3$)

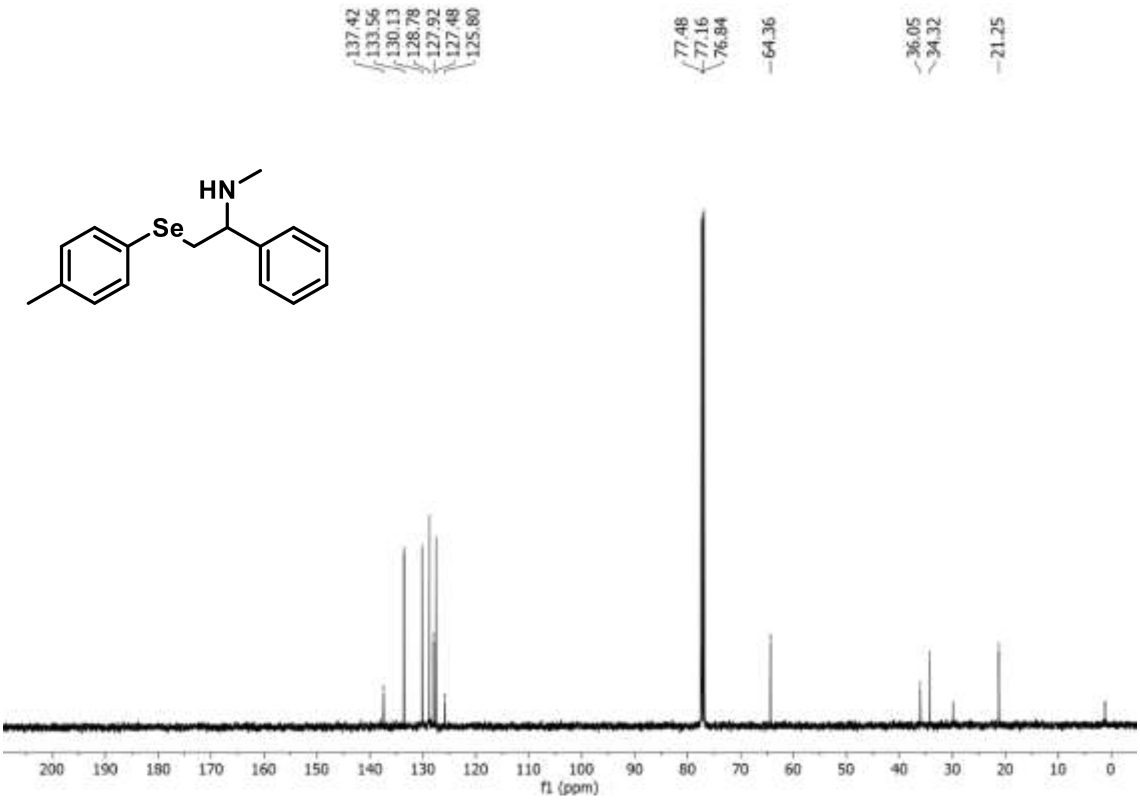

Figure S66. ^{13}C NMR spectrum of **29** (101 MHz, $CDCl_3$)

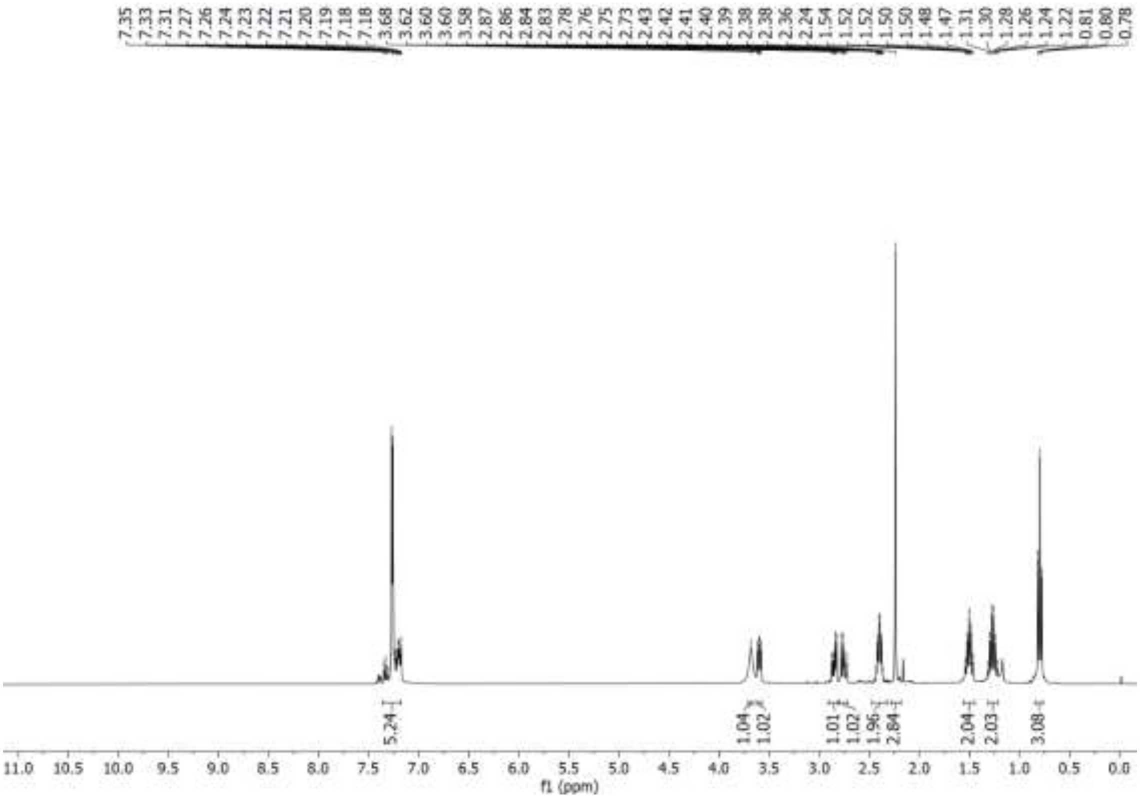

Figure S67. ^{1}H NMR spectrum of **30** (400 MHz, $CDCl_3$)

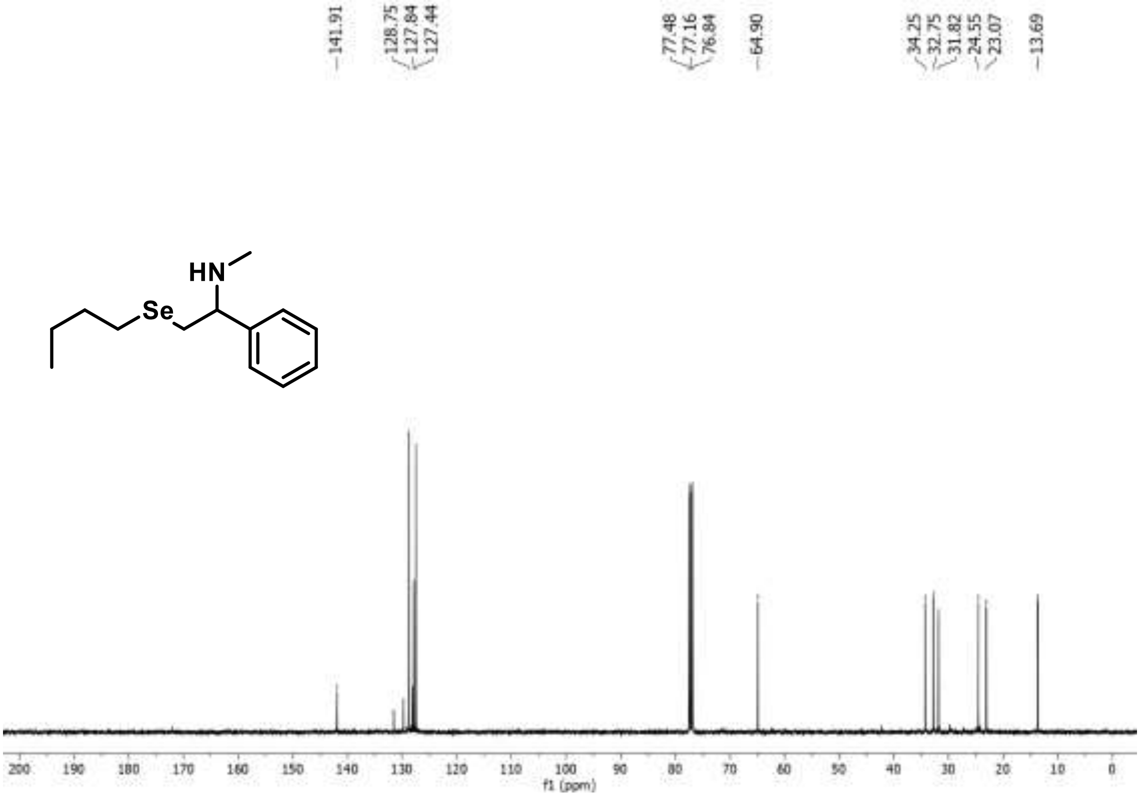

Figure S68. ^{13}C NMR spectrum of **30** (101 MHz, $CDCl_3$)

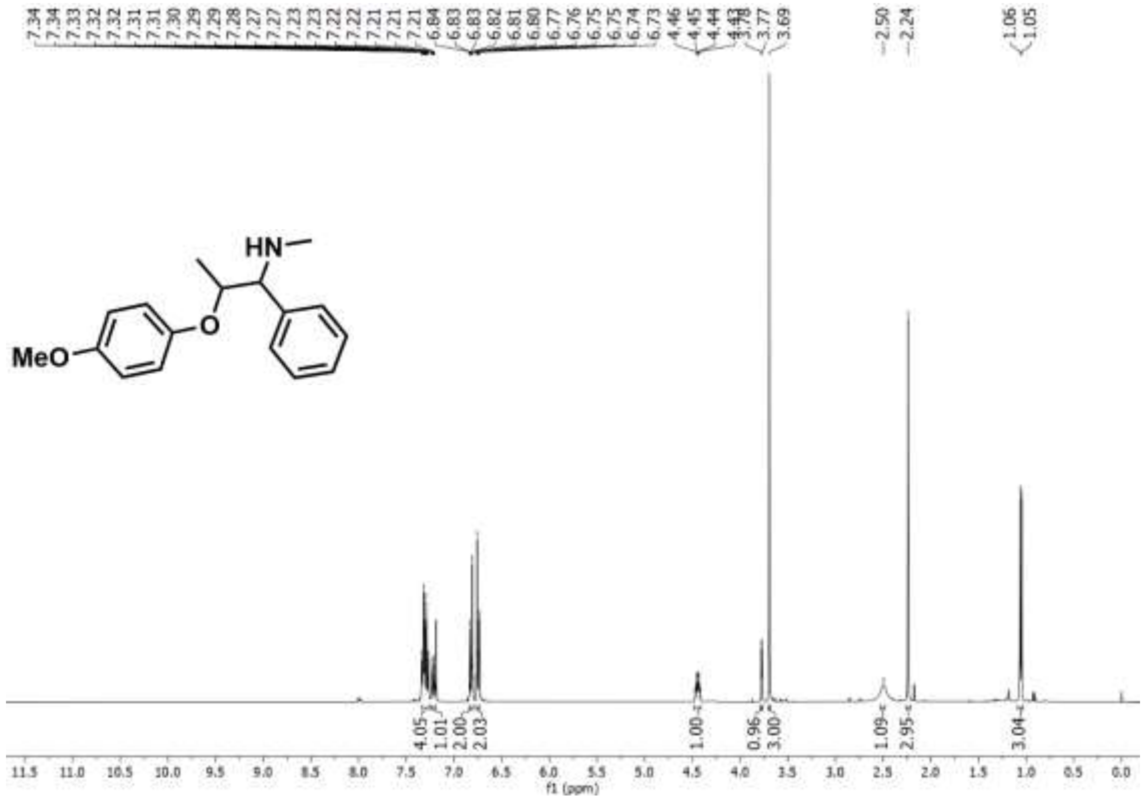

Figure S69. ^{1}H NMR spectrum of **31** (400 MHz, $CDCl_3$)

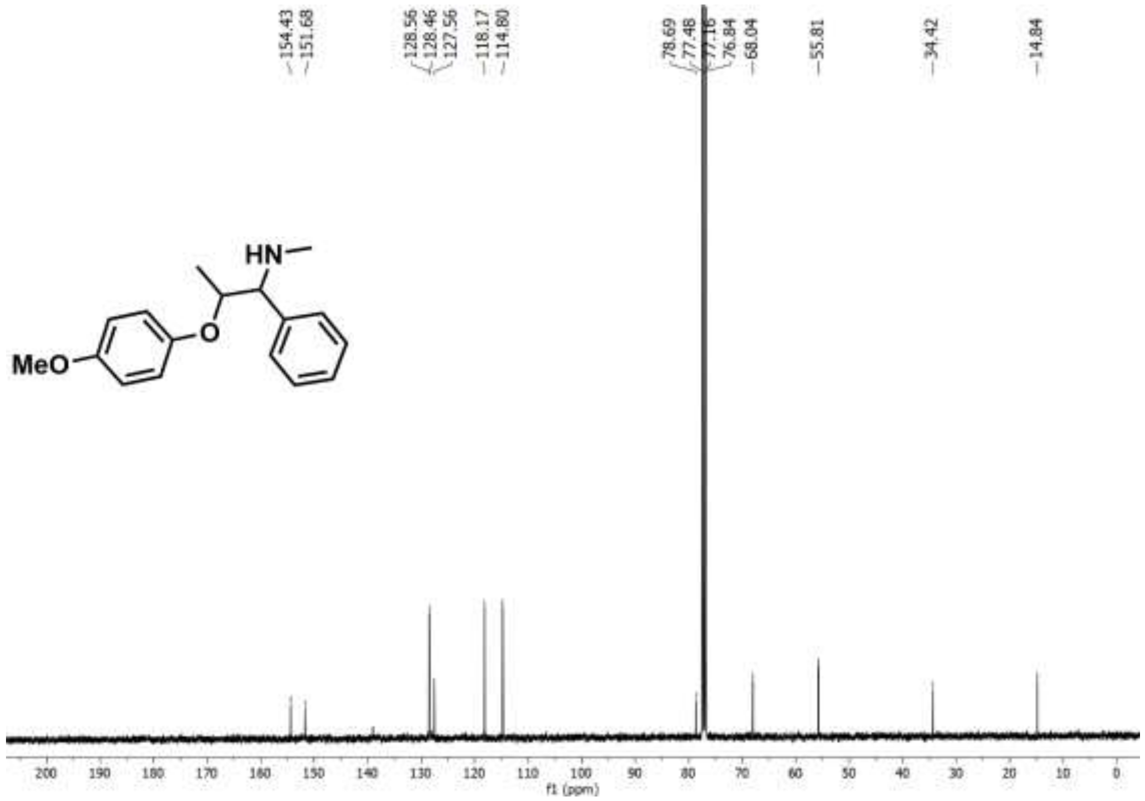

Figure S70. ^{13}C NMR spectrum of **31** (101 MHz, $CDCl_3$)

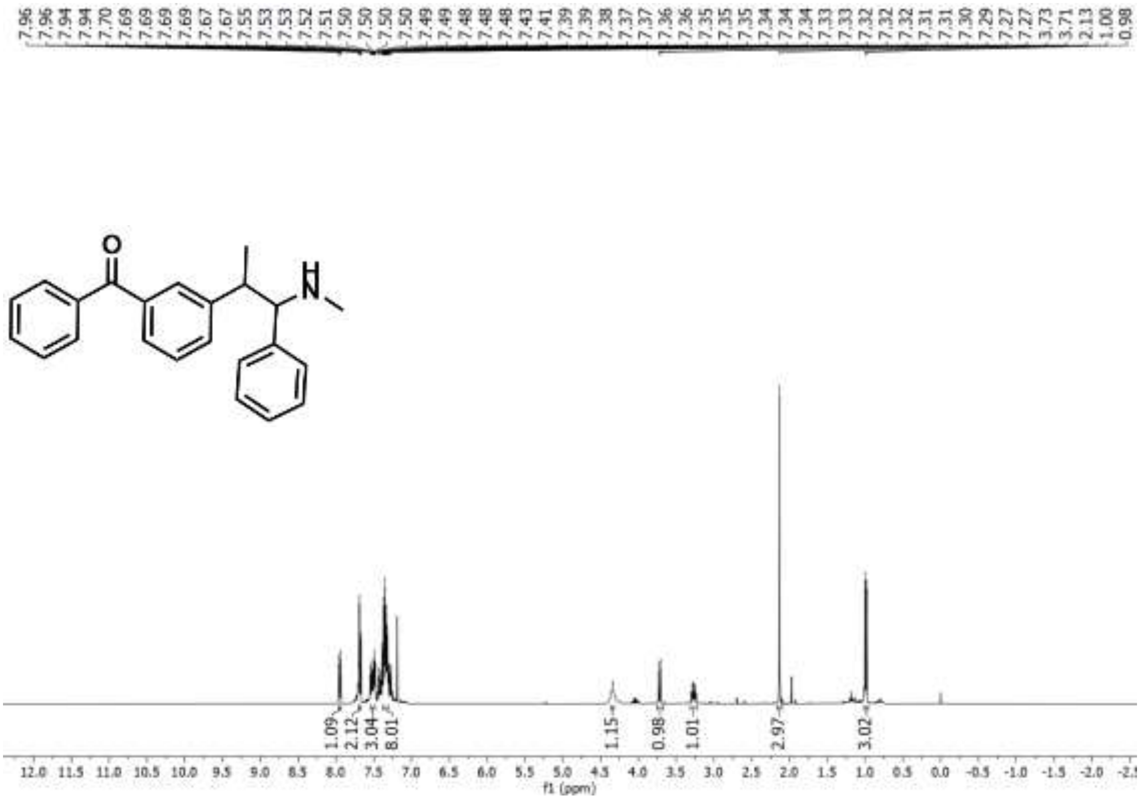

Figure S71. ^{1}H NMR spectrum of **32** (400 MHz, $CDCl_3$)

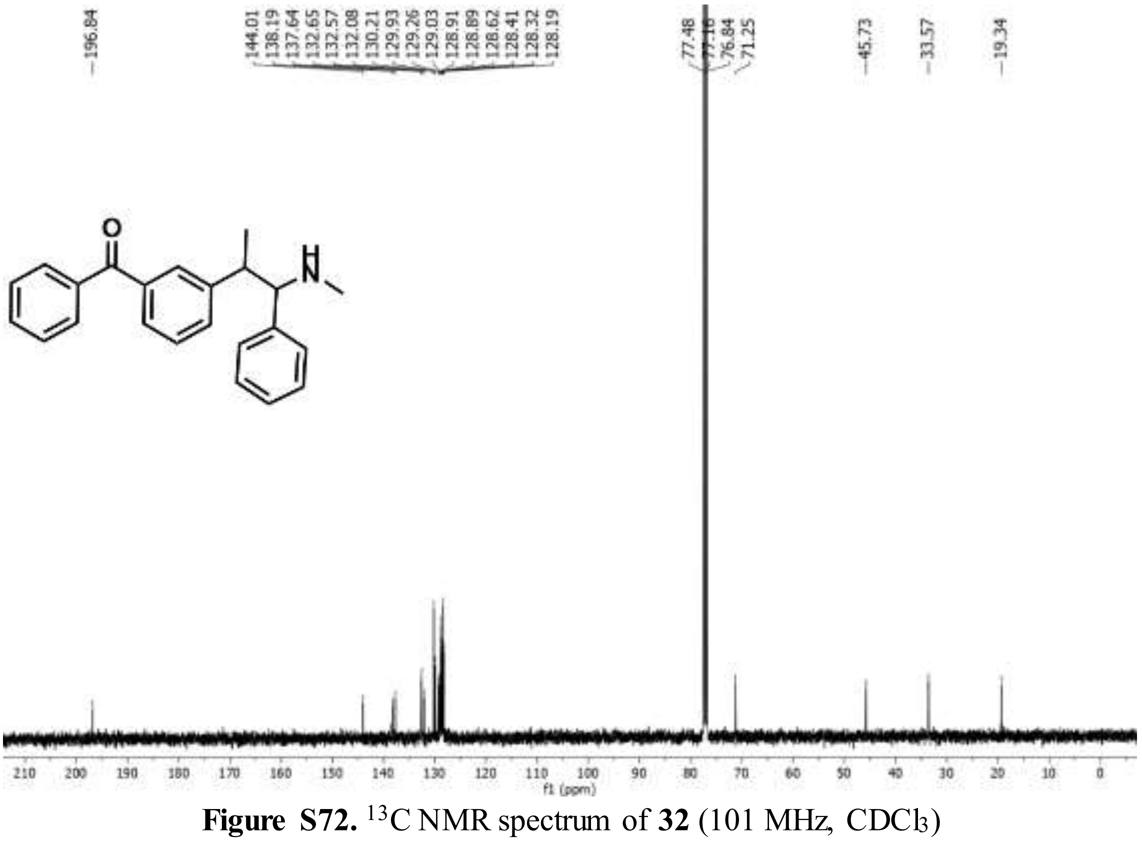

Figure S72. ^{13}C NMR spectrum of **32** (101 MHz, $CDCl_3$)
